# Spatial distribution of the proteome in the human body and in cancers

**DOI:** 10.1038/s41586-026-10660-y

**Published:** 2026-06-17

**Authors:** Liang Yue, Wenhao Jiang, Sainan Li, Meng Luo, Ning Fan, Xiaolu Zhan, Rui Sun, Honghan Cheng, Zhangzhi Xue, Tong Liu, Qianhe Zhou, Kexin Chen, Tian Lu, Fang Guo, Dongwei Li, Weigang Ge, Zongxiang Nie, Mengge Lyu, Jun A, Yingrui Wang, Yingdan Chen, Zhenhai Fu, Nan Xiang, Lu Li, Fengchao Yu, Guo Ci Teo, Alexey I. Nesvizhskii, Meng Wang, Michael P. Snyder, Ben C. Collins, Qi Xiao, Ruedi Aebersold, Fei Xu, Hui Yang, Sijia Zhang, Yi Han, Yi Zhu, Yong Ji, Yan Li, Tiannan Guo

**Affiliations:** 1 Affiliated Hangzhou First People’s Hospital, State Key Laboratory of Medical Proteomics, School of Medicine, Westlake University, Hangzhou, China; 2 Westlake Center for Intelligent Proteomics, Westlake Laboratory of Life Sciences and Biomedicine, Hangzhou, China; 3 Research Center for Industries of the Future, School of Life Sciences, Westlake University, Hangzhou, China; 4 Department of Anatomy, College of Basic Medical Sciences, Dalian Medical University, Dalian, China; 5 Harbin Medical University Cancer Hospital, Harbin, China; 6 Department of Gynecology, Southern Medical University Shenzhen Hospital, Shenzhen, China; 7 Department of Gynecology, Shenzhen Qianhai Taikang Hospital, Shenzhen, China; 8 Key Laboratory of Tropical Translational Medicine of Ministry of Education, School of Basic Medical Sciences, Hainan Academy of Medical Sciences, Hainan Medical University, Haikou, China; 9 Key Laboratory of Biological Targeting Diagnosis, Therapy and Rehabilitation of Guangdong Higher Education Institutes, Fifth Affiliated Hospital of Guangzhou Medical University, Guangzhou, China; 10 Westlake Omics (Hangzhou) Biotechnology, Hangzhou, China; 11 Department of Pathology, University of Michigan, Ann Arbor, MI USA; 12 Gilbert S. Omenn Department of Computational Medicine and Bioinformatics, University of Michigan, Ann Arbor, MI USA; 13 Stanley and Judith Frankel Institute for Heart and Brain Health, University of Michigan, Ann Arbor, MI USA; 14 Department of Genetics, Stanford University School of Medicine, Stanford, CA USA; 15 School of Biological Sciences, Queen’s University Belfast, Belfast, UK; 16 Institute of Molecular Systems Biology, ETH Zurich, Zurich, Switzerland; 17 Faculty of Science, University of Zurich, Zurich, Switzerland; 18 Department of Anatomy, College of Medical Sciences, South China University of Technology, Guangdong, China; 19 Department of Neurosurgery, Huashan Hospital, Fudan University, Shanghai, China; 20 State Key Laboratory of Frigid Zone Cardiovascular Diseases, Laboratory of Cardiovascular Disease and Molecular Intervention, Harbin Medical University, Harbin, China; 21 Department of Critical Care Medicine, Second Affiliated Hospital, Harbin Medical University, Harbin, China; 22 Department of Emergency and Critical Disease, Songjiang Research Institute, Shanghai Key Laboratory of Emotion and Affective Disorders, Songjiang Hospital Affiliated to Shanghai Jiao Tong University School of Medicine, Shanghai, China; 23 Department of Anatomy and Physiology of College of Basic Medical Science, Shanghai Jiao Tong University, Shanghai, China

**Keywords:** Proteomics, Tumour biomarkers, Systems analysis, Target identification

## Abstract

A detailed, spatially resolved quantitative map of the human proteome is essential for a deeper understanding of human biology and disease^1–4^. Here we present a comprehensive human proteomic landscape, generated by profiling more than 13,000 proteins across 2,856 samples using data-independent acquisition mass spectrometry. The dataset spans 58 major tissue types, 251 specific tissue subtypes and 25 distinct carcinomas. This resource enables the depiction of spatially resolved proteome trajectories across tissue types and physiological states, including fetal, tumour, adjacent non-tumour and healthy adult tissue, thereby providing insight into both developmental processes and oncogenic progression. Furthermore, quantitative proteomics comparisons across diverse tissue types and states facilitate the indication of organ-specific toxicity, the identification of repurposable anticancer drug candidates and the prioritization of therapeutic targets for cancers. This study establishes a quantitative resource for navigating the proteome in the human body and in common cancers.

## Abstract (web summary)

A spatially resolved map of the human proteome across a variety of healthy tissues and cancers provides wide-ranging insights in developmental biology and oncology, and could aid the identification of therapeutic targets and development of treatments for cancer.

## Main

Grounded in a genetic blueprint, diverse human tissues and organs with distinct functions arise during development. These functions can become dysregulated in pathological conditions, such as tumours. Characterizing proteomic variation across tissue types in both developmental and pathological contexts is essential for enhancing our understanding of human biology and advancing therapeutic development. Although transcriptomic repositories, such as ArrayExpress^5^, RNA-Seq Atlas^6^ and the BioGPS portal^7^, have provided initial annotations for tissue expression, and the Adult GTEx project has further expanded this using genomic and transcriptomic data from tissue sites that are not affected by disease^8–10^, mRNA abundance correlates only moderately with the expression of proteins, which are the main functional and druggable molecules.

The Human Protein Atlas (HPA), which was launched in 2005, began by incorporating immunohistochemistry-based proteomic data from healthy and cancerous tissues, and has since expanded continuously^11^. By 2015, the HPA had integrated transcriptomic data from 32 healthy tissue types and proteomic data, based on 20,456 antibodies, from 44 healthy tissue types^3^. Although antibody-based protein measurement has an advantage in that it provides localized protein information, its semi-quantitative nature limits the reliable quantification of thousands of proteins, particularly those for which effective antibodies are lacking. By contrast, mass spectrometry (MS) offers a comprehensive, multiplexed and unbiased alternative for quantitative proteomic measurement^12,13^.

In 2014, two MS-based human proteome drafts reported the identification of approximately 85% of proteins encoded by human genes across around 30 tissue types and cell lines^1,2^. A few years later, another study^14^ characterized 15,210 protein groups across 29 tissues using label-free data-dependent acquisition MS, and, more recently^4^, researchers quantified 12,027 proteins across 32 tissue types using tandem mass tag-based MS. Although these studies advanced human tissue proteomics, they focused on a limited set of approximately 30 major tissues, leaving many uncharted, and lacked comprehensive comparisons between healthy and cancerous tissue.

**Fig. 1:**
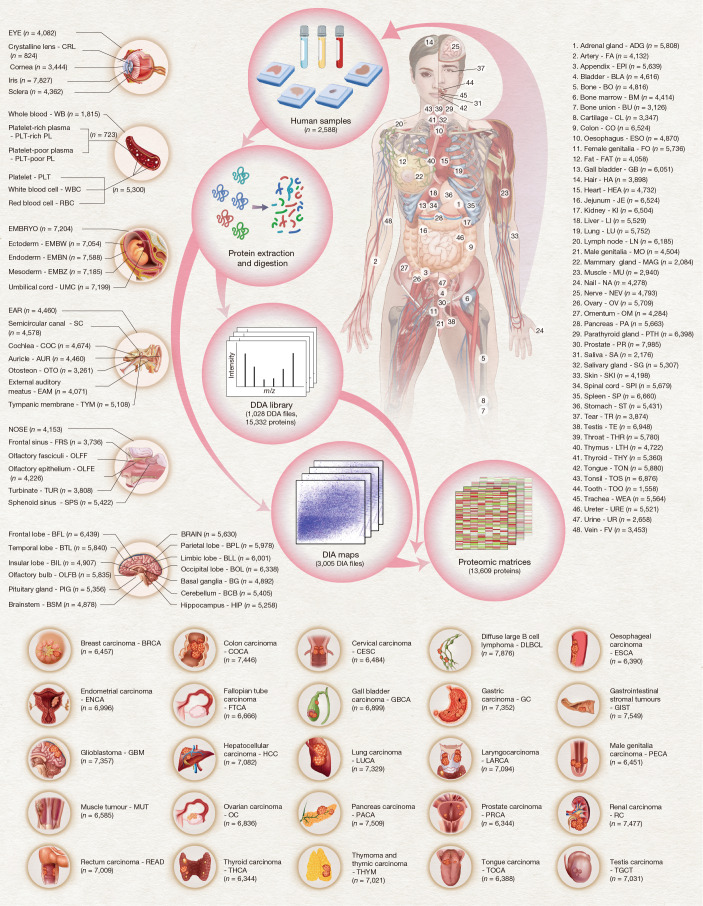
Overview of the draft human proteome. Summary of the sample types in our data. Each sample type is marked with a corresponding number. The specific sampling positions of the eye, blood, human embryo, ear, nose and brain are listed on the left. Twenty-five types of cancer are shown at the bottom. All samples are labelled with the full and abbreviated names of the organ, tissue or cancer, and the numbers in parentheses indicate the number of proteins retained in each submatrix that were quantified in more than 50% of the samples in the specific tissue types. In this figure, ‘female genitalia’ is used as a category label for the fallopian tubes, uterus and vagina, and ‘male genitalia’ is used as a category label for the epididymis, seminal vesicle, seminiferous duct and Cowper’s gland. Components of this figure were created in BioRender; Guomics Lab https://BioRender.com/hwyxuac and https://BioRender.com/96s0zl0 (2026); other components were created using Magnific. Source data

Consortia such as The Cancer Genome Atlas (TCGA), the International Cancer Genome Consortium and the Clinical Proteomic Tumor Analysis Consortium (CPTAC) have generated extensive multi-omics datasets for specific tumours^15,16^; however, challenges in cross-tumour type comparisons limit the insights into differences between cancers that can be obtained from these resources^17^. Comprehensive proteome profiling across diverse human tissues and states requires broad tissue coverage and in-depth, high-throughput proteomics, and this need is addressed effectively by data-independent acquisition mass spectrometry (DIA-MS)^18–20^. Here, using DIA-MS, we present a rich data resource detailing the spatial distribution of 13,609 proteins. This coverage includes 58 healthy adult tissues, paired tumour and non-tumour samples from 25 cancer types, and 22 fetal tissue types, encompassing nearly all solid human tissues, body fluids and major cancer types (Fig. 1 and Supplementary Information). This resource provides a foundation for navigating the human body proteome^21^, and will facilitate cancer drug discovery.

## Samples and proteomic profiling

**Fig. 2:**
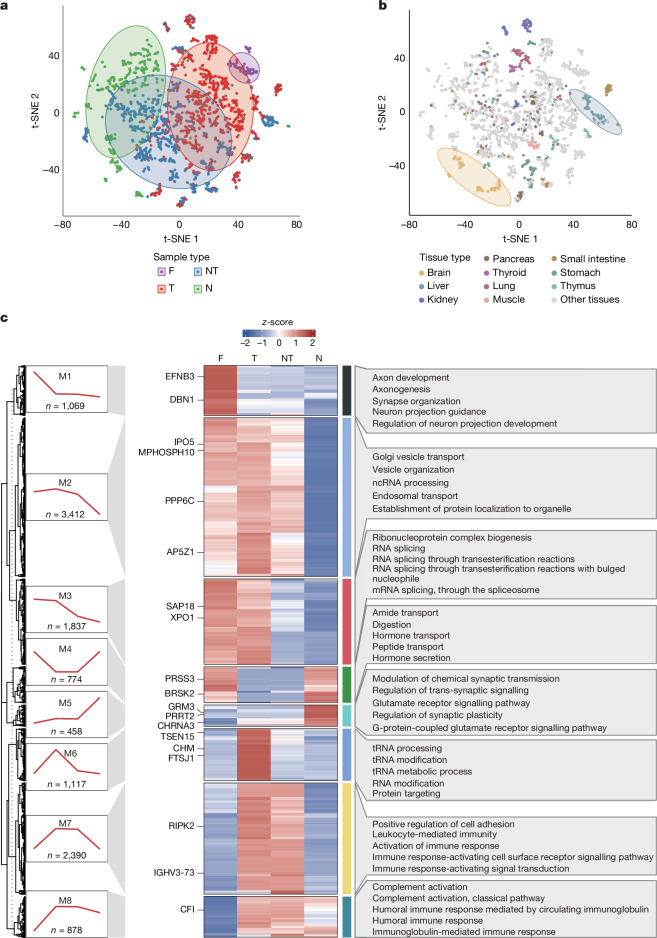
Proteome transformation in tissue development and oncogenesis. **a**,**b**, t-SNE plots of tumour (T), paired non-tumour (NT), normal (healthy) adult (N) and fetal (F) samples with sample type (**a**) and tissue type (**b**) classification highlighted. Shaded areas represent 80% (**a**) and 90% (**b**) confidence ellipses (multivariate *t*-distribution) for each group. **c**, Heat map highlighting trajectory-associated proteins across biological states. The protein matrix was pre-filtered to exclude contaminant proteins, and this was followed by differential expression analysis using a one-way ANOVA across sample types. Significant proteins (B-H adjusted *P* < 0.05) were clustered into eight co-expression modules (M1–M8) on the basis of their expression in F, T, NT and N, with *n* indicating the number of proteins in each module. The *z*-scores reflect relative protein abundance across the columns (F, T, NT and N). On the right, each module is annotated with its top five enriched Gene Ontology: Biological Process (GOBP) terms, ranked by −log_10_ (B-H adjusted *P*) for pathway enrichment. ncRNA, noncoding RNA; tRNA, transfer RNA. Representative proteins belonging to the top-ranked GOBP term of each module are labelled on the left. Source data

**Extended Data Fig. 1:**
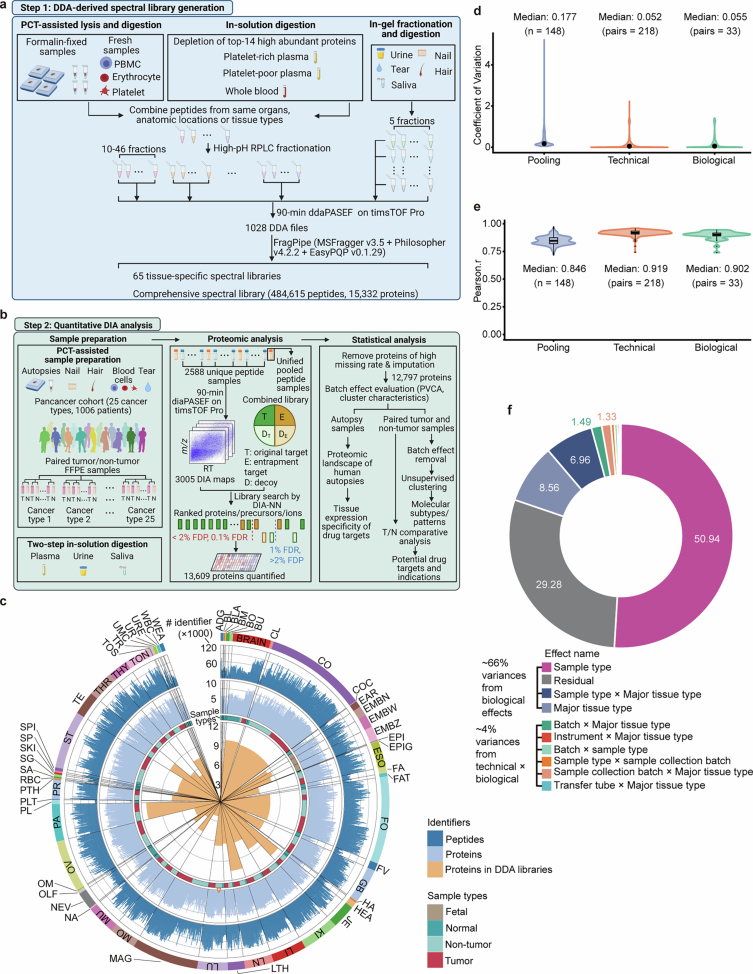
Analysis workflow and data quality. **a**, Workflow of construction of DDA-based spectral library. **b**, Workflow of DIA quantitative data analysis. **c**, Number of identified proteins in ddaPASEF data and quantified proteins in diaPASEF data. Each radius in the figure represents one sample. The radar plot in the centre shows the number of proteins identified in the relative tissue-specific library for each sample. From the centre of the circle, the 1^st^ circle shows the sample type, whether it is a tumour (T), tumour paired non-tumour sample (NT), normal (N), or fetal sample (F). The 2^nd^ circle represents the number of proteins identified in the sample, and the 3^rd^ circle represents the number of peptides identified in the sample. The 4^th^ circle presents the sample-type abbreviations matching those used in Fig. 1. **d**,**e**, CV (**d**) and Pearson’s correlation (**e**) of pooled QC samples and replicates. Box plots display median (centre line), interquartile range (box), and 1.5× IQR (whiskers). In **d**, the centre point indicates the median. **f**, PVCA results generated from the corrected proteomic matrix. The donut chart displays the proportion of variance attributed to major experimental, biological, and interaction effects included in the model. Illustrations in **a**,**b** created in BioRender; Guomics Lab https://BioRender.com/zirfv3r (**a**) and https://BioRender.com/g3kl2ru (**b**) (2026). Source data

**Extended Data Fig. 2:**
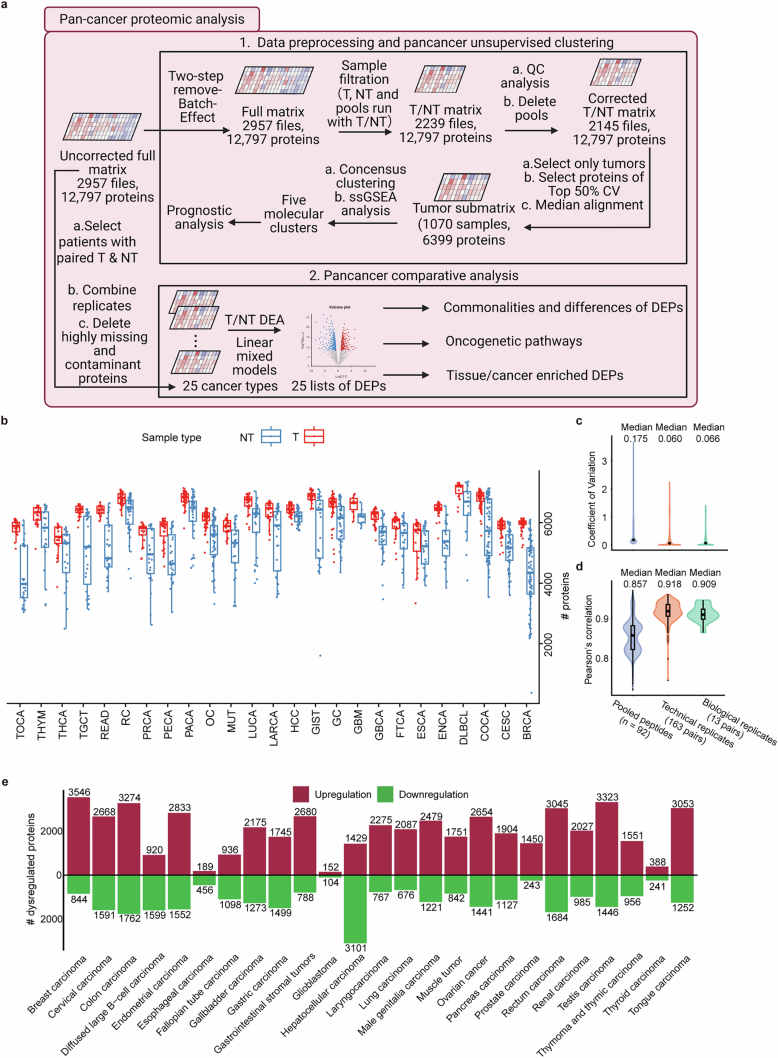
Overview of the pan-cancer quantitative proteomic data. **a**, Workflow of pan-cancer analysis. **b**, The numbers of identified proteins in the tumour and non-tumour samples in 25 cancers. Box plots display median (centre line), interquartile range (box), and 1.5× IQR (whiskers). **c**,**d**, CV (**c**) and Pearson’s correlation (**d**) of pool and replicates. **e**, Numbers of significantly up- and downregulated proteins between paired tumour and non-tumour for each tumour type. Statistical significance determined by linear mixed-effects model with FDR correction with detailed *P* values listed in Supplementary Table 6. Workflow diagram in **a** created in BioRender; Guomics Lab https://BioRender.com/42fsadi (2026). Source data

**Extended Data Fig. 3:**
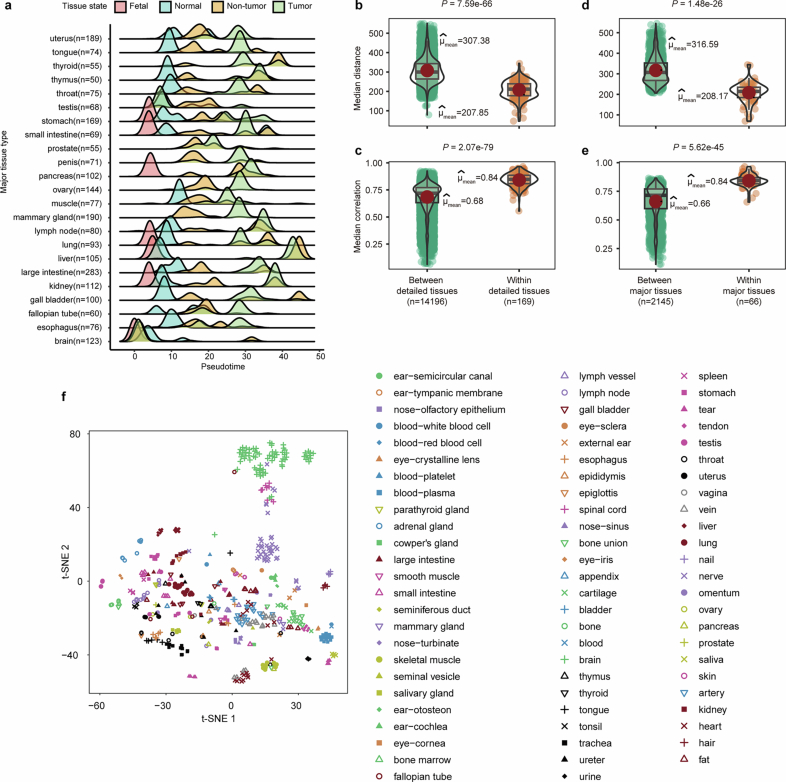
Global proteome-based inter- and intra-tissue heterogeneity. **a**, Ridge plots showing pseudotime distributions across 23 tissue types with multiple tissue states, stratified by sample type (colour-coded). Pseudotime values were calculated through trajectory analysis conducted by Monocle3 based on their proteomic profiles. **b**,**c**, Box plots showing intra- and inter-tissue median Euclidean distances (**b**) and median Pearson correlation coefficients (**c**) calculated for detailed tissue subtypes. **d**,**e**, Box plots showing intra- versus inter-tissue type median Euclidean distances (**d**) and median Pearson correlations (**e**) for major tissue subtypes. Box plots display median (centre line), interquartile range (box), and 1.5× IQR (whiskers). The red circles denote the arithmetic mean for each group. Individual data points representing each tissue pair comparison are overlaid with n denoting the number of tissue pairs. Statistical significance was determined using a two-tailed Welch’s *t*-test. **f**, t-SNE visualization of all normal tissue samples coloured and shaped by major tissue type based on global proteome data. Source data

We collected 2,856 samples from 9 post-mortem adult donors, 8 healthy participants, 9 post-mortem fetal donors and 1,015 patients with cancer (Supplementary Table 1). We first constructed a comprehensive human spectral library from these samples, containing 15,332 protein groups (Extended Data Fig. 1a and Supplementary Information). To ensure the quality of large-scale proteomic data, we searched the DIA-MS raw data against a combined spectral library that integrated the human spectra with entrapment spectra from non-human species (Methods and Supplementary Information). A total of 13,609 proteins were quantified across 3,005 MS files, with a global false discovery rate (FDR) of 0.1% at protein level (Fig. 1, Extended Data Fig. 1b and Supplementary Information). The proteomes showed substantial heterogeneity across tissues and sample types (Fig. 2a, Extended Data Figs. 1c, 2b and 3b–e and Supplementary Information), high reproducibility among replicates and minimal batch effects, indicating high overall data quality (Extended Data Figs. 1d–f and 2c,d and Supplementary Information).

A t-distributed stochastic neighbour embedding (t-SNE) analysis of all the samples showed an orderly arrangement of fetal (F), tumour (T), paired non-tumour (NT) and normal (healthy) adult (N) samples along the opposite direction of the t-SNE 1 axis (F–T–NT–N; Fig. 2a and Supplementary Table 3), mirroring the degree of tissue differentiation. Notably, brain and liver tumours and their paired non-tumour tissues deviated from this F–T–NT–N pattern, clustering together rather than with their respective sample types (Fig. 2b). Next, we applied trajectory analysis to quantify these observations by assigning a pseudotime value to each sample on the basis of its relative position along the developmental trajectory from fetal samples, thereby highlighting tissue-specific F–T–NT–N transitions. Brain tissues exhibited exceptional proteomic stability during malignant transformation and development, characterized by low pseudotime values and minimal variance across all four states (Extended Data Fig. 3a)—consistent with functionally constrained gene expression during brain development^22^. Conversely, liver tumour and non-tumour samples clustered at high pseudotime values, distant from fetal liver, which could be due to the adaptive plasticity of the liver in response to variable environmental factors^22^.

To elucidate the specific proteins and biological processes that underpin this trend, we conducted unsupervised clustering analysis on all of the samples, and identified eight distinct protein modules characterized by coherent expression patterns (Fig. 2c and Supplementary Table 3). Notably, module 3 showed a descending F–T–NT–N trend and was highly enriched for RNA splicing, reflecting its crucial role in both organ development and oncogenesis^23^. By contrast, module 8 exhibited a progressive upregulation along the F–T–NT–N axis and was significantly enriched for the humoral immune response (Fig. 2c), possibly reflecting suppressed or incomplete humoral immunity in prenatal and tumour samples^24,25^. Unsupervised clustering analysis of liver and brain samples separately showed that although decreased RNA splicing and increased immune activation were also observed in the F–T–NT–N trend, tissue-specific functions—such as synaptic transmission pathways in brain samples and metabolic activities in liver samples—were also enriched (Supplementary Information).

## Tissue-specific protein expression

To assess whether subtypes were appropriately grouped into major tissue categories in the normal samples, we compared Euclidean distances and correlation coefficients within and across tissue categories. Tissues exhibiting high within-group heterogeneity, such as eye and cartilage, were further divided into specific subtypes, resulting in 74 refined tissue types (Supplementary Information). All of the refined tissue types showed significantly smaller distances and higher correlations within their respective categories than across categories (Extended Data Fig. 3b–e and Supplementary Information). Global t-SNE embedding showed pronounced inter-tissue differences, with discrete clusters formed by special tissue types such as body fluids, testis, cochlea and semicircular canal (Extended Data Fig. 3f). Physiologically related tissues, such as peripheral nerves, brain and spinal cord, clustered closely, distinct from other tissue types (Extended Data Fig. 3f).

**Fig. 3:**
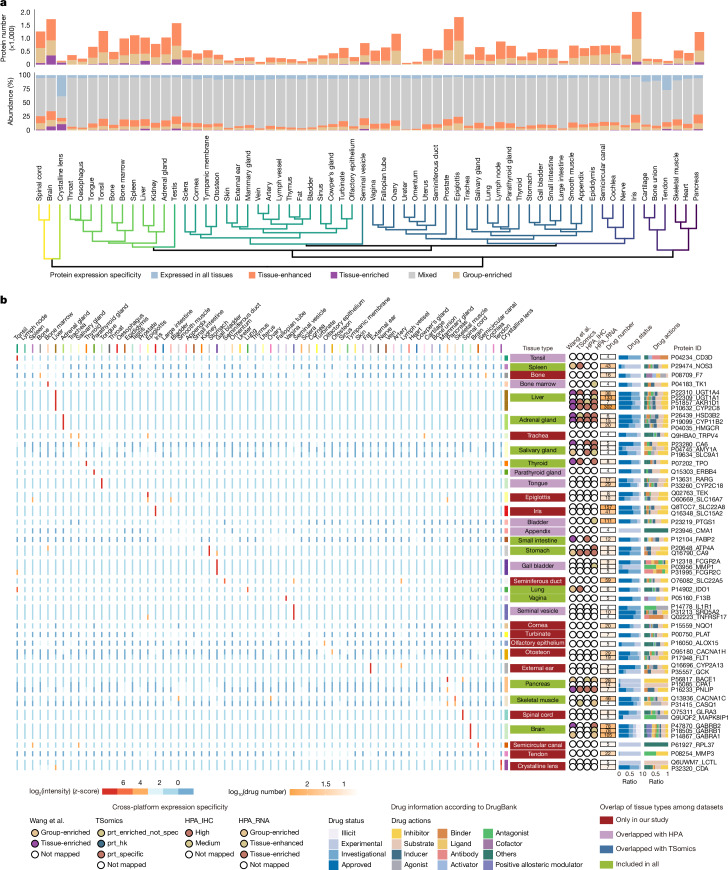
Tissue specificity of protein and drug targets. **a**, Bar plot showing the protein count and summed abundance ratio of each specificity category relative to all quantified proteins in each tissue type. The bottom dendrogram was generated by hierarchical clustering of tissues based on the median abundance of all characterized tissue-enriched proteins in each tissue type. **b**, Heat map showing tissue-specific drug targets. Only the top five most enriched proteins are shown for each kind of anatomical classification. Tissue specificities of the targets are compared with previous drafts of the human proteome: Wang et al.^14^, TSomics^4^, HPA immunohistochemistry data (HPA_IHC)^3^ and HPA RNA-sequencing data (HPA_RNA)^3^. For each drug target, the corresponding drug number and information are shown in coloured bars. prt_enriched_not_spec, tissue-enriched (but not tissue-specific) proteins; prt_hk, housekeeping proteins; prt_specific, tissue-specific proteins. Source data

Next we classified proteins into six groups according to the HPA criteria^3^: not detected, tissue-enriched, group-enriched, expressed in all tissues, tissue-enhanced and mixed (Fig. 3a, Supplementary Table 4 and Supplementary Information). The brain contained the most tissue-enriched proteins, and the crystalline lens showed the highest summed abundance ratio of tissue-enriched proteins to all identified proteins (Fig. 3a). Hierarchical clustering based on tissue- and group-enriched proteins grouped samples that were physiologically related, such as the brain and spinal cord (Fig. 3a). There were some notable exceptions; for example, the clustering of the mammary gland with connective tissue-rich tissues, such as bone, tendon and cartilage; this might reflect age-related involution, which is consistent with the paucity of mammary alveoli observed in haematoxylin and eosin (H&E) staining (Supplementary Information). Similarly, the trachea co-clustered with the salivary gland, probably owing to the prominent presence of glandular epithelial cells in the sampled region of the trachea.

**Extended Data Fig. 4:**
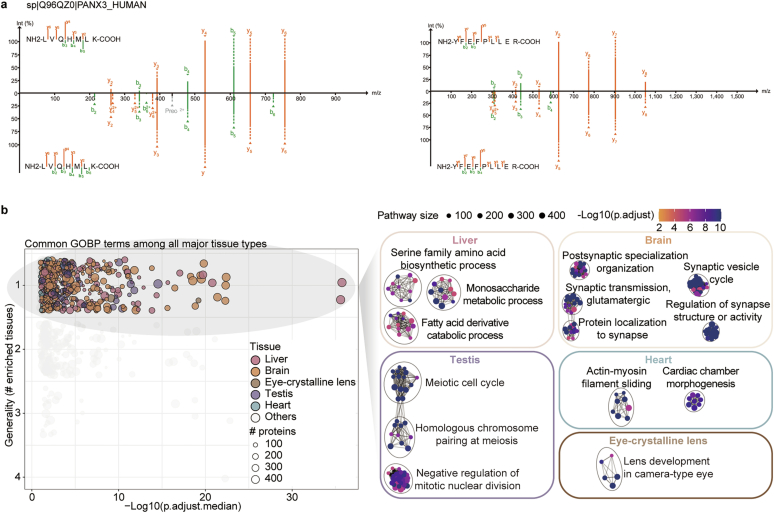
Tissue-enriched proteins and pathways. **a**, Annotated fragment ion spectra of proteotypic PANX3 peptides detected in human tissue samples (upper) and the pooled synthetic peptide sample (bottom). **b**, Functional enrichment of tissue-enriched proteins for each tissue. The *x* axis stands for median -log_10_(B-H adjusted *P*) of each enriched term. The *y* axis stands for the number of tissues enriched in this term. Network analysis of unique pathways enriched only in liver, brain, testis, heart or crystalline lens. Source data

Of the 1,717 tissue-enriched proteins identified, 749 were previously reported as enriched in the corresponding tissue types in published human proteome^4,14^ or transcriptome datasets^3^ (Supplementary Table 5). Of these, 666 were supported at the protein level and 426 showed concordant enrichment in HPA RNA-sequencing data. Across 36 tissues overlapping with the HPA, we identified 832 proteins uniquely enriched in our dataset and 122 exclusively enriched in the HPA RNA data. Notably, 480 tissue-enriched proteins were identified in 24 tissue types that were underrepresented in previous studies, underscoring the expanded tissue coverage. Among these, we identified PANX3, which is at present documented as ‘not detected’ in the HPA dataset^3^, as the top cochlea-enriched protein (Supplementary Table 5). We further synthesized two unique peptides (LVQHMLK and YFEFPLLER) from PANX3 to confirm its presence and its cochlear-specific expression (Extended Data Fig. 4a and Supplementary Table 2). Functional enrichment of tissue-enriched proteins aligned with specialized tissue functions. Proteins associated with metabolism, synaptic function, meiotic cell cycle, cardiac chamber morphogenesis and lens development were uniquely enriched in the liver, brain, testis, heart and crystalline lens, respectively. Hormone metabolic processes were co-enriched in proteins from exocrine organs, including the thyroid and adrenal glands (Extended Data Fig. 4b and Supplementary Table 5).

## Tissue distribution of drug targets

Because tissue-specific drug target expression might contribute to off-target toxicity^26^, we mapped tissue-enriched proteins to DrugBank^27^ targets, identifying 402 proteins corresponding to 2,598 drugs across 34 tissue types (Supplementary Table 5). We found that the liver contained the most tissue-enriched drug targets (Supplementary Table 5), potentially explaining the high incidence of drug-induced liver injury. The liver’s unique exposure through portal circulation, in which absorbed drugs reach the liver directly before systemic distribution, further increases its susceptibility to drug toxicity. Cytochrome P450 2C8 (CYP2C8), which was highly enriched in the liver both in this study and in published MS-based proteome drafts^3,4^, is targeted by 302 drugs, including antivirals, antidiabetic agents and anticancer agents (Fig. 3b). Most of these drugs act as inhibitors and substrates—including gemfibrozil (Supplementary Table 5), which functions as an irreversible inhibitor of CYP2C8 (ref. ^28^). Consequently, co-administration of gemfibrozil with CYP2C8-metabolized drugs can induce severe toxicity by increasing plasma concentrations of the drugs by eight- to tenfold^28–30^. Clinically, this manifests as rhabdomyolysis and acute kidney injury when gemfibrozil is combined with statins^29^, or severe hypoglycaemia when it is combined with antidiabetic agents^30^. Furthermore, the sustained metabolic burden on hepatocytes aggravates the risk of drug-induced liver injury, creating a detrimental cycle of impaired metabolism and increasing toxicity^31^.

We investigated potential off-target organ effects by comparing tissue-enriched drug targets with adverse effects reported in epidemiological studies and clinical cases, focusing on tissues other than the primary target or liver. We identified two epidemiological studies associating thyroid dysfunction with exposure to triclosan, a broad-spectrum antimicrobial that is applied topically. One study showed that exposure to triclosan affected thyroid autoimmunity and homeostasis^32^, and the other study reported inverse associations between maternal triclosan exposure and maternal levels of free thyroxine and neonatal levels of triiodothyronine in a birth cohort^33^. Triclosan targets thyroid peroxidase, an enzyme found to be enriched in the thyroid, both in this study and in the two published MS-based proteome drafts^3,4^ (Fig. 3b). Thyroid peroxidase catalyses thyroid hormone synthesis and is crucial for thyroid homeostasis, whereas its perturbation can affect thyroid autoimmunity and hormone levels. Collectively, these findings underscore the utility of mapping the tissue-specific distribution of drug targets to elucidate the molecular aetiology of off-target adverse effects.

## Pan-cancer protein changes

Paired tumour and non-tumour samples were used to generate a pan-cancer proteomic landscape (Extended Data Fig. 2a), providing a pan-cancer proteomic dataset with a unified pipeline and platform, and with a batch design that minimizes multi-batch effects^34^. Quality control demonstrated high reproducibility (Extended Data Fig. 2c,d) and minimal batch effects (Extended Data Fig. 1f). Consensus clustering of the pan-cancer proteome identified the grouping of various squamous carcinomas in cluster 1 and the unique metabolic profile of cluster 3 shared by hepatocellular carcinoma (HCC) and gastrointestinal stromal tumours (GIST; Extended Data Fig. 6a).

**Extended Data Fig. 5:**
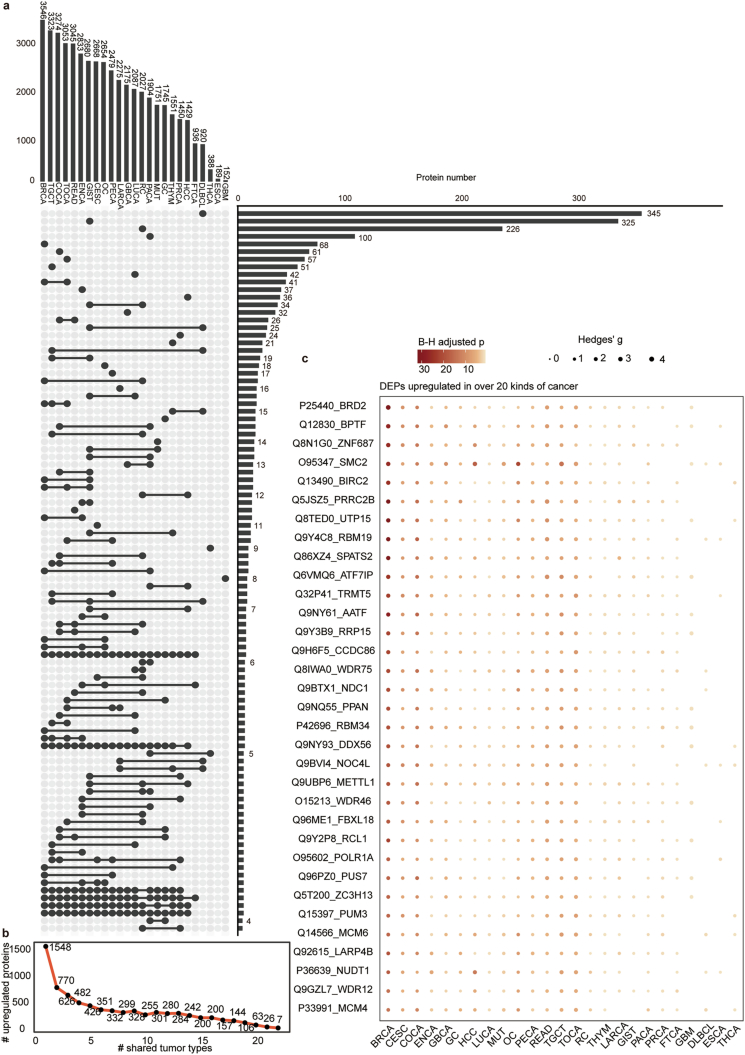
Most common upregulated proteins among tumours. **a**, Upset plot showing the intersections of upregulated proteins shared across different tumor types. Sets are arranged in descending order according to the number of proteins they contain. Sets with the same number are only marked once. Only sets with at least four proteins are shown. **b**, Number of upregulated proteins in each group, with groups defined by the number of organs showing protein upregulation. **c**, Common differential expression illustrated by analysing proteins upregulated in over 20 organs. Statistical significance determined by linear mixed-effects model with FDR correction with detailed *P* values listed in Supplementary Table 6. Source data

Comparative analysis of paired tumour and non-tumour proteomic profiles using a linear mixed model identified 8,940 differentially expressed proteins (DEPs) across 25 tumour types. Colon carcinoma, rectum carcinoma and testis carcinoma had the highest numbers of DEPs, whereas glioblastoma had the fewest (Extended Data Fig. 2e and Supplementary Table 6), consistent with the clustering of T and NT brain samples in the t-SNE map based on the global proteome (Fig. 2b). Most upregulated DEPs were cancer-specific or shared between two cancers (Extended Data Fig. 5a,b). A total of 33 DEPs were upregulated in more than 20 cancer types (Extended Data Fig. 5b,c), including reported drivers of tumorigenesis, such as MCM4 (ref. ^35^) and NUDT1 (also known as MTH1)^36^, indicating that conserved pan-cancer proteomic remodelling can underlie tumorigenesis.

**Fig. 4:**
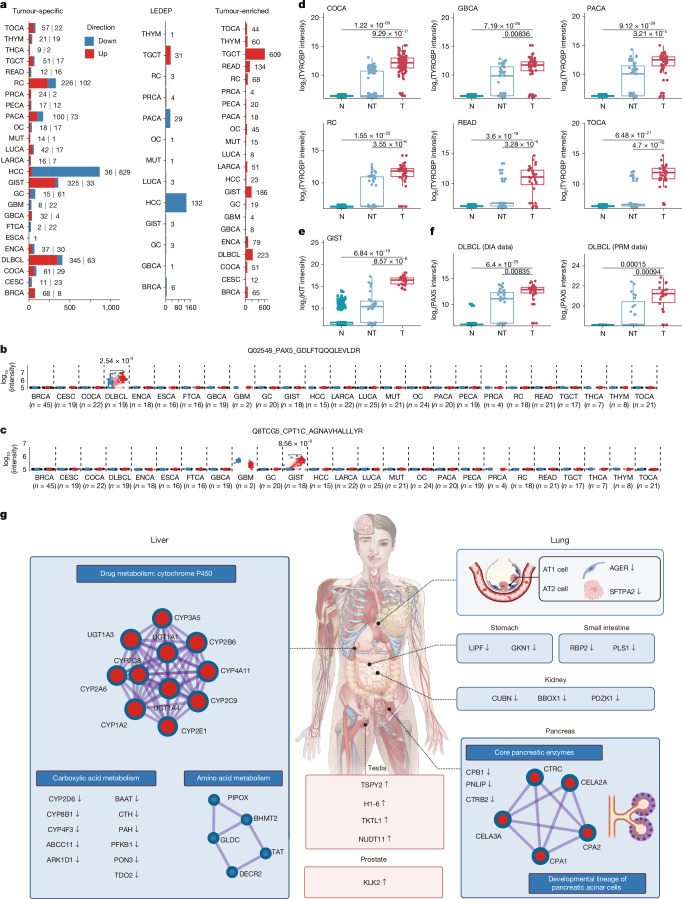
Tumour-specific protein signatures. **a**, Number of tumour-specific DEPs, LEDEPs and tumour-enriched proteins across cancer types. **b**,**c**, Parallel reaction monitoring (PRM) validation of cancer-specific protein expression (PAX5 (**b**) and CPT1C (**c**)). Two-tailed paired *t-*tests; B-H adjusted *P* values for multiple comparisons are shown only if less than 0.05. Paired carcinoma-adjacent tissue samples are connected by lines (red, upregulation; green, downregulation). **d**,**e**, Expression profiles of the membrane-localized tumour-enriched proteins TYROBP (**d**) and KIT (**e**), identified from DIA data. **f**, Concordant expression of the tumour-enriched protein PAX5, validated by both DIA and PRM methodologies. Statistical significance was determined by linear mixed-effects model (DIA) and paired *t*-test (PRM), with FDR correction. In **b**–**f**, box plots show protein abundance distributions: centre line, median; centre point, mean; box limits, 25th and 75th percentiles; whiskers, 1.5 × interquartile range; individual data points overlaid. *n* denotes the number of patients. For the tumour-enriched statistical analysis, tumour and normal samples were analysed without merging technical replicates, as detailed in Supplementary Tables 3 and 6. **g**, Hypothetical systems view of the cancer-specific LEDEPs in several organs. Up and down arrows represent significant upregulation or downregulation in tumour compared with paired non-tumour samples. Blue and red blocks represent activated or suppressed biological activities, respectively, inferred by cancer-specific LEDEPs. Colours of circles represent different module networks derived from cancer-specific LEDEPs in the same tissue type. AT1 cells, alveolar type 1 cells; AT2 cells, alveolar type 2 cells. The protein–protein interaction network was constructed using Metascape-based enrichment analysis of cancer-specific LEDEPs, with functional annotations of modules labelled in blocks adjacent to the corresponding network clusters. The schematic representation of the human body was manually drafted and integrated into BioRender. All other graphical elements and the final layout of this panel were created in BioRender; Guomics Lab https://BioRender.com/hkqg5rr (2026). Source data

**Extended Data Fig. 6:**
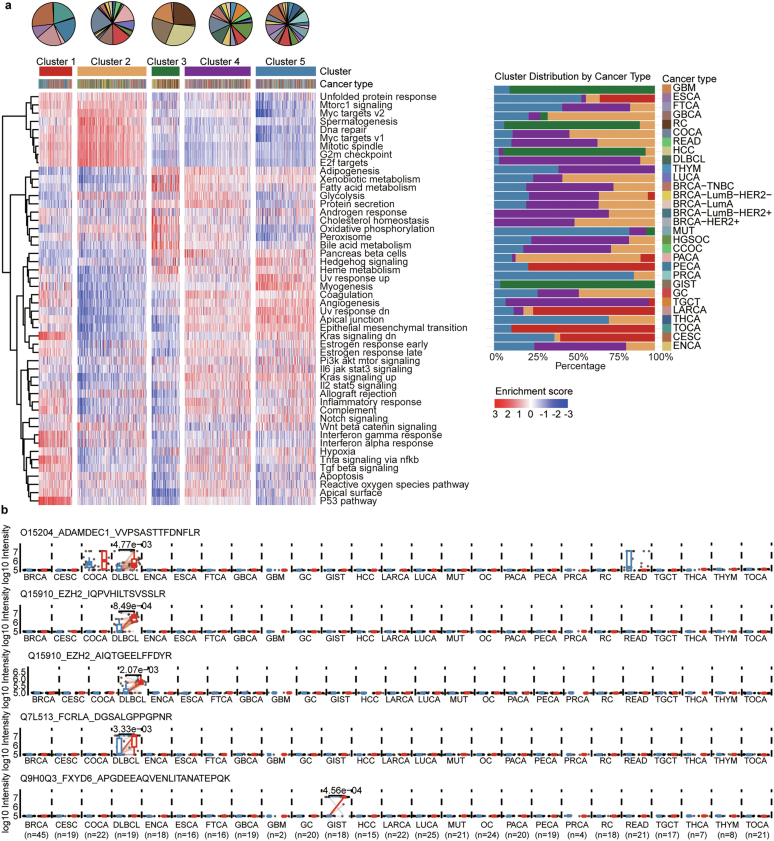
ssGSEA enrichment and PRM validation. **a**, Heat map displaying ssGSEA enrichment scores for hallmark gene sets across five identified clusters. Pie charts indicate the proportion of tumours per cluster. Stacked bar plots show the distribution of each cancer type across clusters. Colour scale represents enrichment scores (−3 to 3). **b**, PRM validation of cancer-specific protein expression. Box plots display protein abundance distributions: centre line, median; centre point, mean; box limits, 25th and 75th percentiles; whiskers, 1.5× interquartile range; individual data points overlaid. Paired carcinoma-adjacent tissue samples are connected by lines (red, upregulation; blue, downregulation). Two-tailed paired *t*-tests were used, with B-H adjustment for multiple comparisons. The notation n denotes the number of patients. Source data

Out of the DEPs across 25 tumour types, 2,878 were tumour-specific, meaning that they were significantly up- or downregulated in only one specific tumour type (Fig. 4a and Supplementary Table 8). HCC had the most tumour-specific DEPs, followed by diffuse large B-cell lymphoma (DLBCL) and GIST, whereas oesophageal carcinoma had the fewest. Most of the HCC-specific DEPs were downregulated and enriched in metabolic pathways (Supplementary Table 8). Of note, synaptic signalling (GO: 0099536, *q* = 1.10 × 10^−4^) was enriched among the GIST-specific upregulated DEPs, consistent with the origin of GIST from the interstitial cells of Cajal (ICCs)—gut pacemaker cells that have neuronal properties. Furthermore, KIT and ANO1, established markers for ICCs^37,38^, were identified as GIST-specific upregulated DEPs (Supplementary Table 8), reflecting the specific cell of origin. Targeted MS-based quantification validated the cancer-specific upregulation of CPT1C and FXYD6 in GIST; both were expressed predominantly in neurons (Fig. 4c and Extended Data Fig. 6b), confirming the enhanced neuronal properties seen in GIST. Among these tumour-specific DEPs, 131 were tissue-enriched in the corresponding normal tissue, representing locally enriched DEPs (LEDEPs). Downregulated tumour-specific LEDEPs reflected the loss of specialized tissue functions, consistent with the dedifferentiation in T inferred from the F–T–NT–N trend (Fig. 2a). For example, RBP2 and PLS1 were suppressed only in small-intestine tumours (GIST); LIPF and GKN1 were reduced specifically in gastric carcinoma; and exocrine markers (for example, CELA2A, CPA1 and PNLIP) were downregulated uniquely in pancreatic carcinoma (Fig. 4g). Conversely, upregulated LEDEPs were observed mostly in testis carcinoma (Fig. 4a), and included TSPY2 (ref. ^39^), suggesting the exploitation of intrinsic germ cell proliferative programs.

**Extended Data Fig. 7:**
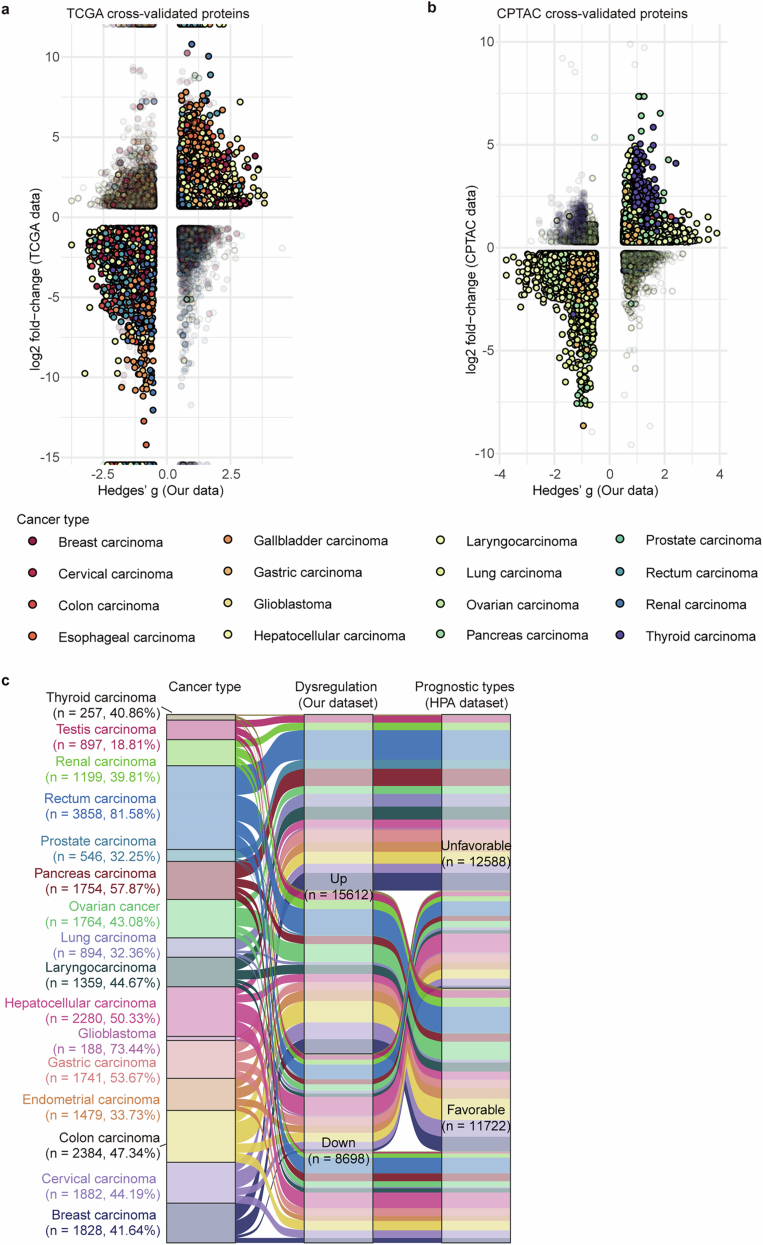
Dysregulated proteins in our data, TCGA and CPTAC. **a**,**b**, Cross-validation in TCGA (**a**) and CPTAC (**b**) datasets. **c**, Overlap with HPA pathology dataset, with Sankey diagram colours indicating different cancer types and black used for text when the corresponding colour lacks sufficient contrast. Source data

We cross-validated the DEPs with TCGA transcriptomic and CPTAC proteomic datasets (Extended Data Fig. 7a,b). Despite differences in platforms and cohorts, we identified 4,263 upregulated and 1,716 downregulated DEPs that were consistent across our data, CPTAC and TCGA. We further integrated our DEP list with prognostic data from the Human Pathology Atlas^40^, and identified 7,336 proteins associated with clinical outcomes across 16 cancer types (Extended Data Fig. 7c and Supplementary Table 7). Notably, PLOD2 was upregulated in 13 of 25 tumours (Supplementary Table 7). A previous pan-cancer study also found that PLOD2 was often upregulated in T compared with adjacent NT tissue, and associated this upregulation with poor prognosis in renal carcinoma, lung adenocarcinoma and pancreatic ductal adenocarcinoma^17^, suggesting that PLOD2 represents a high-confidence candidate for biomarker development and therapeutic targeting.

## Drug repurposing and target discovery

**Extended Data Fig. 8:**
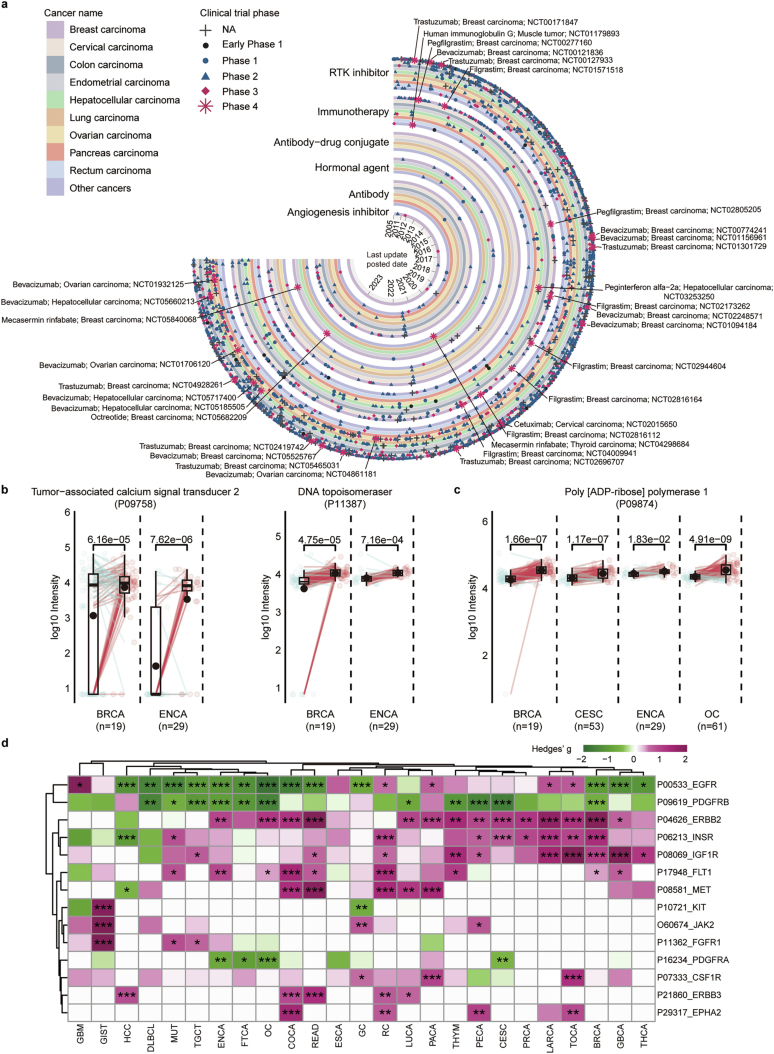
Pan-cancer analysis of dysregulated proteins between paired tumour and non-tumour samples. **a**, DEPs from all cancer types mapped to targets and clinical trials. The clockwise direction represents the start time of clinical trials, circular layers indicate drug types, track colours correspond to cancer types, and symbols denote trial phases. **b**,**c**, Scatter plots showing the expressions of the target of sacituzumab govitecan (**b**) and olaparib (**c**). Points in red and green represent carcinoma and adjacent samples, respectively. Box plots show protein expression; centre lines indicate the medians and centre points indicate the means; box limits represent the first and third quartiles; whiskers extend 1.5 times the interquartile range from the quartiles to outliers; translucent scatters show all sample points. Coloured lines connect the carcinoma-adjacent pairs, while red lines represent up-regulations and blue lines represent down-regulations. The notation n indicates the number of patients. **d**, RTK dysregulations. The colour of a square represents Hedges’ g and non-detected cases are coloured in grey. The B-H adjusted *P* values are indicated by stars, * *P* < 0.05, ** *P* < 0.01, *** *P* < 0.001. Exact *P* values are provided in Supplementary Table 6. The coefficient for sample type in each linear mixed-effects model was tested using a two-sided *t*-test with Satterthwaite-approximated degrees of freedom, and the resulting *P* values were B-H adjusted. Source data

Next, we sought to demonstrate the application of this data resource in drug target prioritization. First, we examined the therapeutic relevance of upregulated DEPs shared among tumours. We screened 77 such DEPs targeted by 36 biomolecular drugs, mostly receptor tyrosine kinase (RTK) inhibitors, which were evaluated in 2,084 clinical trials (Extended Data Fig. 8a and Supplementary Table 7). Notably, many fewer drugs are approved or under investigation for endometrial carcinoma (ENCA) than for breast carcinoma (BRCA) (Extended Data Fig. 8a), highlighting an unmet therapeutic need. Two examples illustrate the repurposing potential. First, Trodelvy (sacituzumab govitecan), an antibody–drug conjugate that combines a humanized anti-TROP2 antibody with the TOP1 inhibitor SN-38, is approved by the US Food and Drug Administration for triple-negative breast cancer. Notably, TROP2 and TOP1 are co-upregulated in both ENCA and BRCA (Extended Data Fig. 8b), suggesting that Trodelvy could be efficacious in ENCA. This hypothesis is supported by a phase II clinical trial (NCT04251416)^41^ and is being further evaluated in a phase III clinical study (NCT06486441). Similarly, olaparib, a PARP1 and PARP2 inhibitor approved for ovarian cancer and BRCA, might have therapeutic potential in ENCA, given the upregulation of PARP1 across multiple gynaecological cancers, including BRCA, ovarian carcinoma, ENCA and cervical carcinoma (Extended Data Fig. 8c). This inference is further supported by the promising responses seen in phase I and II clinical trials^42,43^.

**Fig. 5:**
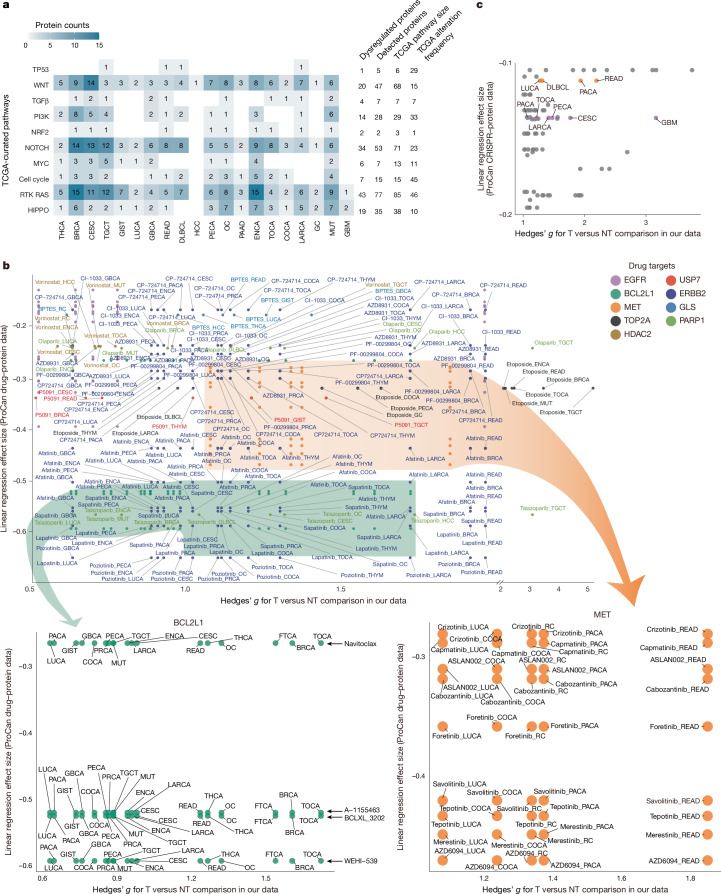
Potential drug targets and drug repurposing. **a**, Number of dysregulated proteins in TCGA-curated pathways of each kind of cancer. **b**,**c**, Druggable carcinoma-dysregulated targets mapped to ProCan-DepMapSanger drug–protein (**b**) and CRISPR–protein (**c**) association data. The colour of a point represents its gene name. The drug–protein association results for BCL2L1 and MET are shown at the bottom. In **b**,**c**, the *x* axis shows Hedges’ *g*, estimated from a fitted linear mixed-effects model as the effect of sample type (T versus NT), standardized by the residual s.d. and corrected for small-sample bias based on the model degrees of freedom. The *y* axis shows *β*, calculated as the ordinary least-squares regression coefficient for the protein abundance term in the linear model relating protein abundance to drug response or CRISPR–Cas9 gene essentiality. Source data

Second, we integrated our dataset with drug sensitivity data and CRISPR gene essentiality data to prioritize candidate protein targets and drugs. Specifically, we mapped upregulated DEPs to the ProCan-DepMapSanger proteomic dataset^44^, which links protein abundance with both half-maximal inhibitory concentration (IC_50_) values (drug sensitivity) and CRISPR–Cas9 gene essentiality in pan-cancer cell lines. This integration identified a total of 35 drugs targeting 9 DEPs, predominantly RTKs (Fig. 5b and Supplementary Table 9). Given the frequent occurrence of RTK alterations at both the gene and the protein level (Fig. 5a and Extended Data Fig. 8d), we narrowed our focus to RTKs. We prioritized those RTKs that were simultaneously (1) significantly upregulated in tumours (Hedges’ *g* ≥ 0.5, Benjamini–Hochberg (B-H) adjusted *P* < 0.05) and (2) predictive of a higher drug potency at a higher protein intensity (*β* < −0.1, FDR < 0.01; Fig. 5b). We found that high expression of MET and BCL2L1 correlated with increased potency of savolitinib, tepotinib and merestinib (MET inhibitors) and navitoclax (a BCL2 family inhibitor) in colorectal carcinoma cell lines. In rectum carcinoma, the therapeutic viability of targeting MET was further supported by the correlation between MET protein levels and CRISPR gene essentiality, with higher abundance correlating with greater dependency (*β* < −0.1, FDR < 0.01; Fig. 5c).

Third, we aimed to minimize adverse effects by prioritizing tumour-enriched proteins that were significantly upregulated in tumours relative to both paired non-tumour tissues and all of the normal tissues. We identified 41 unique tumour-enriched proteins located at the plasma membrane (Supplementary Table 8). Several are already targeted by clinically approved antibody–drug conjugates (for example, CD79B)^45^, supporting this prioritization strategy. The remaining candidates, particularly uninvestigated transmembrane proteins, represent novel therapeutic targets. TYROBP, a transmembrane signalling adaptor identified as tumour-enriched in ten tumour types (for example, colorectal, gastric, renal and pancreatic carcinoma, Fig. 4d and Supplementary Table 8), is a potential shared antibody–drug conjugate target, although its myeloid expression requires toxicity evaluation. KIT, the established pacemaker of ICCs, was identified as both GIST-specific and GIST-enriched (Fig. 4e and Supplementary Table 8), indicating the therapeutic potential of targeting aberrant ICC-like signalling in GIST. Targeted proteomic analysis confirmed PAX5 as tumour-specific and tumour-enriched in DLBCL (Fig. 4b,f). PAX5 maintains B cell identity, prevents terminal differentiation and contributes to lymphomagenesis through sustained transcriptional activation^46^. Evidence also shows that PAX5 inhibition enhances the efficacy of BTK blockade in DLBCL preclinical models^47^, highlighting its potential as a therapeutic target in this malignancy.

## Conclusions

Here we present an anatomically resolved human proteome atlas spanning four pathophysiological states including fetal, healthy adult, tumour and paired non-tumour tissues, providing a unified resource to delineate tissue-specific proteomic distribution and prioritize therapeutic targets. We identified 1,717 tissue-enriched proteins across 74 refined tissue types, including 480 from previously uncharacterized tissues. The spatial distribution of tissue-enriched drug targets provides a molecular reference for interpreting potential organ-specific toxicities. This data resource allowed us to identify possible therapeutic targets, including 41 tumour-enriched proteins with potentially minimal off-target toxicity. Our comprehensive proteome landscape of the human body and prevalent cancers represents a key first step in the creation of a digital navigator for the human body.

We acknowledge several limitations of our study. The normal samples are predominantly from older donors, which might affect the presentation of some tissues, such as the mammary gland, owing to involution. In addition, specialized protocols were used for certain tissue types, such as hair and body fluids, because of their unique characteristics^48^. These protocol variations might introduce artefacts in cross-tissue comparisons, and we therefore avoided prioritizing tissue-enriched proteins from these tissues. Our focus on comprehensive tissue coverage limited the number of patients per tumour type, constraining the characterization of inter-patient heterogeneity. Nevertheless, our uniform platform and standardized pipeline ensure data quality and comparability, and our findings will improve researchers’ understanding of tumorigenesis in multiple organs, as well as aiding in cancer drug target discovery.

## Methods

### Collection of tissue samples

The autopsy samples involved in this study were collected from the body donation centre of Dalian Medical University and Shanghai Jiao Tong University. The post-mortem interval was within 10 h. The project was approved by the Ethics Committee of Dalian Medical University (institutional review board (IRB): Dalian Medical 2019-05) and Shanghai Jiao Tong University (IRB: 2022-A-01, 2021-03). Written informed consent was obtained from the legally authorized representatives for all donors. To ensure anatomical accuracy, we used (1) macrodissection by two experienced anatomists (included in the author list) following a standardized protocol; and (2) histopathological validation through H&E-stained formalin-fixed paraffin-embedded (FFPE) sections reviewed independently by two pathologists (included in the author list) for tissue types that might not be clearly partitioned at the anatomical level.

Paired tumours and non-tumour samples from surgery were collected from Harbin Medical University Cancer Hospital (from December 2019 to December 2022) and Huashan Hospital (from January 2018 to December 2018). The project was approved by the Ethics Committee of Harbin Medical University Cancer Hospital (IRB: KY2019-08, KY2023-03 and KY2019-16) and Huashan Hospital (KY2021-064). Written informed consent for each patient was collected. The samples mentioned above were stored in FFPE format. We systematically compared the cancer types, number and types of samples of our pan-cancer cohort with those in TCGA^15^ and CPTAC^49^. According to the NCI Genomic Data Commons^50^, our study includes two rare malignancies not covered by either TCGA or CPTAC: GIST and fallopian tube carcinoma. Conversely, TCGA and CPTAC include several cancer types absent in our current cohort, including adrenal cortical carcinoma, bladder cancer, leukaemia, mesothelioma, skin cancers and melanoma. As regards sample size, our study profiles approximately 40 patients per cancer type (Supplementary Table 1), whereas TCGA typically includes around 200 cases per type and CPTAC includes about 160. With respect to normal control tissues, TCGA mainly uses blood samples for germline genomic analysis, whereas CPTAC often includes matched adjacent non-tumour tissues (either fully or partially paired). In our pan-cancer cohort, we incorporated matched adjacent non-tumour tissues for most patients, allowing tumour–non-tumour comparisons at the proteome level.

Autopsy specimens of fetuses, from abortions, were collected from Shenzhen Baoan District Maternal and Child Health Hospital (during 16 June 2020 to 16 September 2020). The post-mortem interval was within 10 h. The project was approved by the Ethics Committee of Shenzhen Baoan District Maternal and Child Health Hospital (IRB: LLSCHY 2019-10-36). Written informed consent was obtained from all donors of tissues. The fetal samples were stored in formalin at 4 °C before use.

### Sample preparation for LC–MS

The sample preparation process was similar to that described previously^51–53^. In brief, we punched each FFPE sample and weighed about 1.0 mg. Heptane was used for dewaxing, gradient (100%, 90%, 75%) ethanol for hydration and 0.1% formic acid (FA) for acid hydrolysis. The obtained product was then placed in pressure cycling technology (PCT) microtubes, and Tris-HCl (pH 10, freshly prepared) was added at 95 °C for 30 min for alkaline hydrolysis. After rapid cooling, lysis buffer (6 M urea, 2 M thiourea), Tris(2-carboxyethyl)phosphine (TCEP) and iodoacetamide (IAA) were added for reductive alkylation. PCT-assisted lysis was performed in the Barocycler NEP2320-Enhanced (Pressure BioSciences) for 90 cycles at 30 °C, with each cycle consisting of 30 s at high pressure (45,000 psi/~310 MPa) followed by 10 s at ambient pressure (AP). LysC (enzymatic substrate concentration ratio 1:80, Hualishi Tech) and trypsin (enzymatic substrate concentration ratio 1:20, sequencing grade, Hualishi Tech) were then added for digestion. The process was conducted with PCT for 120 cycles at 30 °C, with each cycle consisting of 50 s at high pressure (20,000 psi/~138 MPa) followed by 10 s at AP. After the enzymatic hydrolysis process was terminated by 10% trifluoroacetic acid (TFA), we used C18 spin columns (The Nest Group) for desalination.

The dried samples were resuspended with buffer A (high-performance liquid chromatography (HPLC)-grade water, pH 10). The fractionation was performed using an UltiMate 3000 RSLCnano system (Thermo Fisher Scientific) with an HPLC C18 column (diameter 4.6 mm, length 25 cm, particle size 300 Å). The flow rate was 0.5 μl per min. The gradient was 5% to 95% buffer B (98% acetonitrile (ACN), pH 10) in 60 min. A total of 60 fractions were collected. Then, 60 fractionations were combined into 10. The combined samples were evaporated using vacuum centrifugation (CentriVap, Labconco) at 45 °C.

For bone tissues, we cut a piece approximately one-fifth of the volume of a 2 ml centrifuge tube. The teeth were shattered with a clean hammer and placed into the 2 ml tube. After the dewaxing and alkaline hydrolysis described above, we added lysis buffer and grinding beads (about one-third of the volume of the 2 ml tube). The sample was frozen in liquid nitrogen, ground in a tissue grinder for 1 min and refrozen in liquid nitrogen. This procedure was repeated three to five times until the sample turned into a bone slurry (except for the teeth). We then added 1 ml of 10% FA solution and incubated the sample at 4 °C overnight for digestion. Afterwards, approximately 4 mg of the insoluble wet weight was transferred to a PCT tube, and 150 µl Tris-HCl (pH 10) was added to neutralize any excess acid, followed by removal of the supernatant. Then, 20 µl Tris-HCl (pH 10) and 30 µl 6 M urea–2 M thiourea lysis buffer were added, and subsequent PCT-assisted lysis and enzymatic digestion were performed as described above.

Hair samples (4 cm) or nail samples (1 mg) were immersed in 50% methanol or ethanol, chopped, and vortexed, then transferred to a PCT tube. To the tube, 30 µl lysis buffer (30% trifluoroethanol, Tris-HCl buffer, pH 8) and 2.5 µl of 200 mM TCEP solution were added, and the tube was sealed with a PCT pestle and placed in a PCT device. The samples underwent 180 cycles in the Barocycler (50 s at 45,000 psi (~310 MPa), 10 s at atmospheric pressure, 70 °C). After cooling to room temperature, 2.5 µl of 800 mM IAA solution was added, and the samples were incubated in the dark at room temperature for 30 min with gentle mixing (800 rpm). After dilution with 150 µl Tris-HCl buffer (pH 8), 1.25 µg of LysC and 5 µg of trypsin were added, and the tube was sealed and subjected to 120 cycles in the PCT (20,000 psi (~138 MPa) for 50 s, atmospheric pressure for 10 s, 30 °C). After the cycles, 15 µl of 10% TFA solution was added to stop the digestion, and the samples were desalted using C18 tips.

Whole blood was collected by venipuncture into commercial EDTA-containing sampling containers. It was separated into PRP1, erythrocyte, platelet-rich plasma (PRP), platelet-free plasma (PPP) and the pure platelet fraction, according to a previously published article^54^. PRP1 was the supernatant in the first centrifuge at 200*g* for 10 min. PRP was the supernatant in the centrifuge of PRP1 at 200*g* for 10 min. Then, 1 ml whole blood was transferred from the anticoagulation tube into a 50 ml centrifuge tube, 10 ml 1× red blood cell lysate (Invitrogen, 00-4333) was added for 10 min at room temperature (strictly controlled time) and 25 ml phosphate-buffered saline (PBS; total volume 36 ml) was added to stop the reaction. It was then divided into 15 ml centrifuge tubes (9 ml in each tube) and centrifuged at 500*g* for 15 min at 4 °C, followed by aspirating and discarding the supernatant. At this time, 200 μl was allowed to remain in each 15 ml centrifuge tube. The remaining liquid and cell pellet from each tube were pipetted into a 1.5 ml centrifuge tube, which was centrifuged at 500*g* for 5 min at 4 °C, followed by aspirating the supernatant as much as possible, to leave the cell pellet. The cell pellet was washed with 1× PBS three times and centrifuged (500*g* for 5 min, 4 °C) to get a clean white blood cell pellet.

The first stage of the process was the depletion of the top 14 proteins in the blood, PPP, PRP, and PRP1: samples taken out from storage at −80 °C were immediately put on ice. The depletion column was equilibrated to room temperature, following the manufacturer’s instructions (Thermo Fisher Scientific, High-Select Top14 Abundant Protein Depletion Resin, A36371). The second stage was protein concentration, following the manufacturer’s instructions (Thermo Fisher Scientific, Pierce Protein Concentrators PES, 3K MWCO, 0.5 ml, 88512). Then, 500 μl of 8 M urea–100 mM ammonium bicarbonate was added to the concentrator, which was spun at 12,000*g* for 40 min. Finally, the remaining volume was adjusted to 50 µl. The third stage of the process was protein digestion, which was done as described in a previous study^55^; however, the TCEP and IAA processes were both 40 min. Then, the protein extracts were digested with LysC and trypsin to the sample at 1:50. Digested peptides were cleaned using C18 (Thermo Fisher Scientific, 60209-001). The fourth stage was peptide fractionation, as described previously^56^. In brief, peptides were separated into 122 fractions, which were consolidated into 10 fractions. The samples were separated at a flow rate of 1 ml per min using a gradient from 5% ACN in 10 mM ammonia (pH 10.0) to 8% in 7.5 min; the percentage was increased to 18% in 30 min and to 32% in 55 min, then to 95% in 61 min; this 95% composition was held for 6 min; and finally it was dropped to 5% in 68 min. The fractions were combined according to the following strategy: (1) combine the 1st to 6th, 77th to 82nd and 97th, 98th, 107th and 108th fractions; (2) combine the 7th to 10th, 73rd to 76th, 93rd, 94th, 111st and 112nd fractions; (3) combine the 11th to 14th, 69th to 72nd, 89th, 90th, 115th and 116th fractions; (4) combine the 15th to 18th, 65th to 68th, 83rd, 84th, 121st and 122nd fractions; (5) combine the 19th to 22nd, 61st to 64th, 99th, 100th, 105th and 106th fractions; (6) combine the 23rd to 26th, 57th to 60th, 95th, 96th, 109th and 110th fractions; (7) combine the 27th to 30th, 53rd to 56th, 91st, 92nd, 113rd and 114th fractions; (8) combine the 31st to 34th, 49th to 52nd, 85th, 86th, 119th and 120th fractions; (9) combine the 35th to 38th, 45th to 48th, 101st, 102nd, 103rd and 104th fractions; (10) combine the 39th to 44th, 87th, 88th, 117th and 118th fractions. The combined fractions were subsequently dried and redissolved in 2% ACN/0.1% FA, and then analysed by liquid chromatography–mass spectrometry (LC–MS) (Bruker, timsTOF Pro).

For the collection of saliva, we strictly controlled the conditions to minimize contamination from food debris and oral bleeding, according to the instructions from Salimetrics (https://salimetrics.com/saliva-collection-handbook/). In brief, we collected about 1 ml of saliva per person from four healthy participants before eating meals and at least 45 min after brushing teeth, keeping records of the start and end times. Participants had to avoid the following before saliva collection: strenuous exercise, oral issues, intake of a high-sugar diet, intake of a highly sour diet, coffee, alcohol, nicotine and drugs. We collected 1 ml of the middle part of the morning urine. The saliva and urine samples were stored in at −80 °C before use. The reduction, alkylation, digestion, and desalting steps were performed as described for plasma samples in a previous study^55^.

### LC–MS/MS analysis

The peptides redissolved in 98% water:2% ACN:0.1% FA (v/v/v) were separated and analysed by a coupled ultra-high-performance liquid chromatography (UHPLC)–trapped ion mobility spectrometry–tandem mass spectrometry (MS/MS) system. For each acquisition, the peptides were loaded onto a Thermo Fisher Scientific Trap Cartridge (100 Å 5.0 μm, 0.3 mm × 5 mm) and separated by an in house-made analytical column (120 Å 1.9 μm, 150 mm × 0.075 mm) with a Bruker Daltonics nanoElute UHPLC system using a 95 min liquid chromatography gradient at a flow rate of 0.3 μl per min. The composition of the gradient was as follows: linearly increased from 2% B to 22% B in the first 80 min, then from 22% B to 35% B in 10 min, and then from 35% B to 80% B for 2 min and maintained at 80% B for the last 3 min. The mobile phase A was 99.9% water:0.1% FA (v/v), whereas mobile phase B was 99.9% acetonitrile:0.1% FA (v/v). All reagents were MS grade.

A Bruker Daltonics trapped ion mobility quadrupole time-of-flight mass spectrometer (timsTOF Pro) was used to perform data acquisition. In both data-dependent acquisition (DDA) and DIA mode, the MS full scans were acquired over an *m*/*z* range of 100 to 1,700 and an ion mobility range of 0.60 to 1.60 V per s per cm^2^. The ion mobility over *m*/*z* heat maps were filtered using an inclusion–exclusion polygon region with vertices (150, 0.6), (1,300, 1.6), (1,700, 0.6), and (1,700, 1.6). In DDA parallel accumulation–serial fragmentation (ddaPASEF) mode, the method consisted of an MS full scan followed by 10 PASEF MS/MS scans, which consumed 1.17 s of total cycle time. The accumulation time and ramp time were both 100 ms. The target intensity was 20,000, and the intensity threshold was 2,500. In DIA parallel accumulation–serial fragmentation (diaPASEF) mode, the PASEF MS/MS scans were collected from 64 windows in an *m*/*z* range of 400 to 1,200 and corresponding ion mobility range of 0.57 to 1.47 V per s per cm^2^, adapted from the standard 16 diaPASEF scans scheme^57^.

### Database searching and library generation

For generating consensus PASEF spectral libraries, we used the FragPipe^58–61^ computational platform (v.18) with MSFragger^60,61^ (v.3.5), Philosopher^59^ (v.4.2.2) components and the EasyPQP (https://github.com/grosenberger/easypqp/) (v.0.1.29) Python package. Our workflow was based on the FragPipe DIA_SpecLib_Quant workflow^58^, modified accordingly.

The database searching of raw files was performed by the MSFragger search engine, referring to the UniProtKB/Swiss-Prot *Homo sapiens* proteome ID UP000005640 (containing 20,377 canonical protein sequences; downloaded on 7 May 2020), appended with reverse protein sequences as decoys generated by the built-in Philosopher decoys generation tool. Then, Philosopher was used for further evaluation, protein inference and two-dimensional (2D) FDR filtering with a threshold of 0.01; the picked FDR algorithm was used to scale FDRs for large datasets. The built-in library generation script gen_con_spec_lib.py coupled with EasyPQP was used to build consensus PASEF spectral libraries with 0.01 FDR control on both the peptide level and the protein level. Most of the parameters were maintained as reported previously^62^, except for the application of the semi-enzymatic tryptic digestion rule, a 0.05 Da fragment mass tolerance and retention time (RT) alignment based on spiked indexed RT (iRT) peptides. Thus, each peptide was identified either as a unique peptide to a specific protein (or protein group) or as a razor peptide to the protein (or protein group) with the most peptide evidence. Correspondingly, each protein (or protein group) was identified by either a unique peptide or a razor peptide detected for it. In downstream DIA analysis, protein groups containing indistinguishable proteins were excluded from the quantification reports.

### Spectral library evaluation

The information on precursors, fragment ions and modifications in the spectral library was analysed to evaluate its quality according to an algorithm modified from the published DIALib-QC tool^63^. The Perl code for visualization was re-implemented in R. The ratios of sequence length, ion charge, peptide modification, fragment ion type and missed cleavage were counted, respectively, and presented in bar charts. The distributions of precursor *m*/*z* and peptide sequence coverage were displayed as histograms, and the protein sequence database read-in was performed by the seqinR^64^ package. Pearson’s correlation of iRT values between +2 and +3 ion charge states of the same peptide sequence was measured (*R*^2^), and the 2D kernel density estimation was performed using the function stat_density2d in the ggplot2 package.

We further compared the proteins and peptides in our spectral library with PeptideAtlas^65^, which consisted of 16,702 proteins and 2,701,150 peptides (as of 6 July 2021), and ProteomicsDB^66^, which covered 13,553 proteins and 913,326 peptides (as of 6 July 2021). The comparison results were presented as a Venn diagram generated by an R script.

To rigorously assess and control false discoveries in the DIA analysis, we applied a combined entrapment strategy^67^. We constructed a spectral library that merged empirical human spectra from the project-specific DDA library with entrapment spectra generated from non-human species^68^. This design produced the desired ratio of entrapment-to-original target entries at both levels: 16.714 for protein groups (256,259 entrapments versus 15,332 targets) and 1.072 for precursors (739,318 entrapments versus 689,568 targets).

### Analysis of DIA data by DIA-NN

DIA searching was performed by DIA-NN (v.2.2.0 Academia) with the FragPipe-generated spectral library, with its existing protein inference results^62^. Both the MS1 and the MS2 mass accuracy were set as 15 ppm, and a robust liquid chromatography (high-precision) quantification strategy and RT-dependent cross-run normalization were chosen for quantitative results. Moreover, the different runs were treated as unrelated with match-between-runs disabled. For a shorter waiting time, the 2,856 sample runs, including replicates, and 149 pooled sample runs were randomly grouped and searched on two computers in parallel. The 3,005 runs were reanalysed with the generated .quant files in a single run to generate the final report.

By default, DIA-NN iteratively selects the best elution peak per precursor using target decoy-trained classifiers, quantifies each precursor by summing the top six fragment intensities and computes protein group intensities from both proteotypic and shared precursors passing the specified *q* value thresholds, with PG.Q.Values estimated using all precursors assigned to each Protein.Group and Protein.Q.Values computed using only proteotypic precursors assigned to the corresponding genes. To increase stringency, we retained only proteins supported by unique peptides (that is, protein groups without ‘indistinguishable’ members in the FragPipe-annotated DDA library), and only proteotypic peptides (proteotypic = 1 in the DIA-NN report).

For downstream quantification and statistical analyses, we filtered the DIA-NN main report using enforced stringent FDR thresholds based on a series of false discovery proportion (FDP) estimates. The FDP values among the targets and entrapment discoveries (*T* ⋃ *E*_T_) were calculated using the equation below:

$${\hat{{\rm{F}}{\rm{D}}{\rm{P}}}}_{T\cup {E}_{{\rm{T}}}}=\frac{{N}_{{\rm{E}}}(1+1/r)}{{N}_{{\rm{T}}}+{N}_{{\rm{E}}}},$$

where *N*_T_ is the number of target discoveries, *N*_E_ is the number of entrapment discoveries and *r* is the entrapment-to-target ratio in the spectral library.

To select suitable *q* value thresholds, we evaluated precursor and global protein-group *q* value cut-offs across the range 1 × 10^−4^ to 0.01, while keeping the run-specific protein-group *q* value fixed at 0.01. To balance sensitivity and confidence, we finally adopted the following criteria: proteotypic = 1, Q.Value < 0.001, PG.Q.Value < 0.01, Global.Q.Value < 0.001 and Global.PG.Q.Value < 0.001. These settings ensured tight global control of the FDP across all reporting levels in our multi-batch DIA dataset.

### Quality control and preprocessing

We used both biological replicates (independent processing of aliquots from the same tissue sample) and technical replicates (repeated injections of identical peptide samples), with random assignment to minimize bias. Samples were randomized by reshuffling each collection batch, and clinical metadata were blinded during processing. Quality control used a dual approach: a project-specific pool prepared by mixing equal amounts of peptides from early-collected samples, and a universal standard (mouse liver digest) monitored by specialized MS administrators. Both quality control samples were injected before each batch to monitor instrument stability. All samples, including project-specific pool samples, were analysed by DIA-NN and preprocessed together (3,005 raw files in total).

Before quality control analysis, proteins with 50% or greater missing values across all the tissue types were removed from the protein matrix. The remaining protein-level intensities were quantile-normalized across samples using the normalize.quantiles function in the preprocessCore^69^ package (v.1.58.0), followed by log_2_ transformation. Missing values were then imputed using 0.5× the minimal value in the whole matrix. Batch effects were estimated using principal variance component analysis and corrected using the removeBatchEffect function provided by the limma^70^ package (v.3.52.4) in two steps. Unless stated otherwise, this corrected matrix is used in all subsequent analyses.

Consistent processes were implemented for the corresponding data from pooled peptide samples to calculate the coefficients of variation (CVs) of protein abundances and Pearson correlation coefficients. For both technical and biological replicates, CVs were calculated by the same method that was used for pooled peptide samples, and Pearson’s *r* was calculated in all pairs (combined number). Similar quality control analysis was subsequently performed with the corrected matrix.

### Protein identification and quantification

The number of proteins in tissue-specific libraries and DIA-quantified peptides and proteins were visualized in circular bar charts using the canvasXpress R package (v.1.29.6) and retouched using Adobe Illustrator.

### t-SNE visualization

The whole matrix, excluding pooled samples, was standardized and dimensionality-reduced using t-SNE with the Rtsne^71^ package (v.0.16) in R. The perplexity parameter was set to 10. The 2D result was visualized in a scatter plot. To visualize group-level spread, we overlaid data ellipses based on a multivariate *t*-distribution for each sample type using stat_ellipse() from ggplot2 (v.4.0.1) in R.

### Trajectory analysis of sample types

Trajectory analysis was performed using Monocle3 (v.1.4.26)^72^ to reconstruct the pseudotemporal ordering of samples on the basis of their proteomic profiles. After removing hair, nail and body fluid samples, the data were first processed with principal component analysis and then embedded with uniform manifold approximation and projection (UMAP) for dimensionality reduction. The trajectory graph was generated without partitions and without loop closing, with fetal samples set as the starting point of the trajectory, and pseudotime values were obtained for all samples.

To identify proteins whose expression changed along the progression of sample types, we applied a one-way ANOVA with FDR adjustment (*P*_adj_ < 0.05) and removed background contaminants. For these significant proteins, we computed their median intensities across the four sample types (F, T, NT and N) and performed *k*-means clustering (*k* = 4–8) using the ClusterGVis^73^ package (v.0.1.4). Each cluster was subsequently annotated through Gene Ontology: Biological Process (GOBP) enrichment analysis, and the top five enriched terms for each cluster were retained for display purposes.

### Tissue heterogeneity analysis

The proteomic heterogeneity of normal samples was assessed using a preprocessed and quality-controlled protein expression matrix. Samples were hierarchically classified by anatomical classification (major category) and specific tissue name (subcategory). We calculated pairwise Pearson correlations (*r*) and Euclidean distances for all samples using the ‘stats’ package (v.4.4.1). We summarized the distribution of these metrics across combinations of two major categories and two subcategories (Supplementary Table 4). Tissue heterogeneity was further investigated by ranking median distances of major tissue types and subcategory pairs within the same major tissue type. Statistical differences in median distances and correlations across these hierarchical groups were evaluated using robust non-parametric comparisons in the ggstatsplot package (v.0.13.6). Subcategories with fewer than two samples were excluded to ensure statistical rigour. Global heterogeneity trends were visualized using multi-panel violin plots, and point-range distributions were generated with ggplot2 (v.4.0.1).

### H&E staining

Sections of 7 μm thickness were cut using a HistoCore AUTOCUT microtome (Leica Biosystems) and mounted on positively charged glass slides. Slides were stained with H&E using the HistoCore SPECTRA Automated Slide Staining Workstation (Leica Biosystems) according to the manufacturer’s standard protocol. In brief, sections were deparaffinized, rehydrated through graded alcohols, stained with haematoxylin to visualize nuclei, differentiated, blued and counterstained with eosin to visualize cytoplasm and extracellular matrix. After staining, sections were dehydrated through ascending ethanol concentrations, cleared in xylene and coverslipped automatically. Stained sections were examined and photographed using a Zeiss Axio Observer 3. Images were captured at 40× magnification and processed by ZEN Blue (v.3.4).

### Comparison with previous human transcriptome and proteome draft

For comparison with previous human proteome drafts, we used Ensembl^74^ stable gene IDs (v.GRCh37.p13) as our primary mapping strategy, ensuring consistency across data versions and minimizing gene-symbol ambiguity. We exclusively used canonical reviewed proteins from UniProt^75^, mapped to stable gene IDs by BioMart^76^, thereby eliminating isoform mismatch issues. For transcriptomic integration, we retrieved the HPA-normalized transcripts per million (TPM) matrix and associated tissue specificity annotations.

To address quantification differences, we implemented two complementary strategies: (1) *z*-score normalization of overlapped proteins before Pearson correlation analysis for cross-study expression comparisons; and (2) comparison of tissue specificity labels derived from independent multi-tissue proteome analyses within each study, following established methodology.

To assess the consistency of tissue specificity annotations across independent datasets, we manually aligned the anatomical classification labels used in our data to the tissue nomenclatures used in previous studies^3,4,14^. We specifically cross-referenced tissue-enriched and group-enriched protein categories for corresponding tissues between datasets. For correlation analyses, the HPA^3^ and Wang et al.^14^ expression matrices were *z*-score-normalized. Because the GTEx^4^ dataset uses a tandem mass tag-based ratio matrix, we performed median normalization before applying *z*-score normalization. From these standardized matrices, we calculated pairwise Pearson correlation coefficients to quantify inter-study proteomic and transcriptomic consistency.

### Tissue specificity analysis

The tissue specificity  analysis was only performed on healthy adult autopsy specimens, excluding pan-cancer specimens, body fluids, hair, nail, umbilical cord and aborted fetus, using the modified HPA classification method^3^. The protein abundances of each tissue type were aggregated by the median of all samples after imputing missing values to 0.5× the minimal value in the whole matrix. We changed the categories of tissue-enriched, group-enriched and tissue-enhanced proteins from fivefold higher abundance to threefold for our more varied tissue types.

Tissue-enriched proteins were extracted for each tissue type and used to compare functional profiles based on GOBP annotations. Enrichment analysis was performed using the clusterProfiler^77^ package (v.4.12.6) in R. The gene sets with genes between 10 and 500 were selected, with a *q* value threshold of 0.05 and a minimum protein count of 3. In the bubble plot, the top five most significant tissue types were highlighted for their uniquely enriched pathways. Network relationships among enriched pathways were generated using the enrichmentNetwork function in the aPEAR^78^ package (v.1.0), applying a Jaccard similarity matrix and hierarchical clustering to identify pathway clusters, and selecting the term with the minimal *q* value as the cluster name.

### Differential expression analysis

Differential expression was analysed separately for each cancer type using only patients with paired tumour and adjacent non-tumour samples. Given the limited sample size per cancer type, inherent tumour heterogeneity and potential confounding from technical batch effects, we applied a linear mixed model for comparative analysis between paired T and NT samples for each cancer type. Batch effects and clinical factors (sex, age and subtype) were explicitly included as covariates in differential expression analyses to ensure robustness, with standardized effects calculated after Hedges’ small-sample correction^79^. This dual strategy enhances the reliability of biological signal detection while minimizing false positives attributable to technical variation^80–82^. We removed proteins with missing values in more than 50% of samples within each cancer type, and imputed the remaining missing values under a left-censoring (limit-of-detection) assumption. Missing values were replaced using a global intensity floor multiplied by a random noise term drawn from a narrow normal distribution. Specifically, the imputed value *I*_imp_ was calculated as:

$${I}_{{\rm{imp}}}={I}_{{\rm{global}},\min }\times 0.5\times {\epsilon },$$

where *I*_global,min_ is the minimum intensity observed across the cancer dataset (the global floor), and *ϵ* represents a random noise term drawn from a narrow normal distribution with a mean of 1 and a s.d. of 0.001.

After log_2_ transformation, we performed differential expression analysis for each protein by fitting a linear mixed model using the lmerTest^83^ package (v.3.1.3), in which the log-scaled intensity was the outcome. The model included sample type (tumour versus non-tumour) as the primary fixed effect and a patient-specific random intercept to account for the paired sample structure. Additional fixed covariates (cancer subtype, sex, age and dataset) were included only if supported by the available data. The tumour–non-tumour contrast was obtained from the coefficient of the sample-type term ($\hat{{\beta }_{1}}$). To quantify the bias-corrected standardized effect size, the Hedges’ *g* statistic^84^ was computed as:

$$g=\frac{\hat{{\beta }_{1}}}{\hat{\sigma }}\times J(\nu ),$$

where $\hat{\sigma }$ is the fitted model’s residual s.d., *ν* denotes the degrees of freedom and *J*(*ν*) is the small-sample bias correction factor defined by the Gamma function (Γ):

$$J(\nu )=\frac{\Gamma (\nu /2)}{\sqrt{\nu /2}\,\Gamma ((\nu -1)/2)}.$$

The *P* values were adjusted for multiple testing within each cancer type using the Benjamini–Hochberg procedure, and proteins were considered significantly DEPs if they satisfied *P*_adj_ < 0.05 and |Hedges’ *g*| ≥ 0.5. Proteins exhibiting statistically significant up- or downregulation in a single tumour type were classified as tumour-specific DEPs. A single protein could be categorized as a tumour-specific DEP in more than one cancer type, exhibiting different regulation patterns (up- or downregulation) across these distinct tumour types. The DEPs corresponding to RTKs were standardized and shown in a heat map with hierarchical clustering. The DEPs were also mapped to TCGA-curated pathways^85^, and the degree of pathway dysregulation was assessed by the protein count for each pathway in each cancer type. Here, we present the pseudocode for this analysis:


Input: Proteomics matrix X, sample metadata MOutput: Per-cancer results R = (c, j, q, g_adj_)FUNCTION EstimableCovariates (M_c_)


V ← ∅


FOR EACH covariate v ∈ {cancer_subtype, sex, age_centered, dataset}:IF |unique values of v in M_c_ | ≥ 2 THEN V ← V ∪ {v} ENDIFRETURN VENDFUNCTIONFOR EACH cancer type c:1. Subset X_c_, M_c_ to samples where cancer_type = c2. Retain only complete tumour–normal pairs (indexed by patient_ID);let n = number of pairs3. Center age: age_c_ ← age − mean (age)4. V ← EstimableCovariates (M_c_)FOR EACH protein j ∈ {1, …, P}:5. Fit linear mixed-effects model via REML:y_ij_ = β_0_ + β × sample_type_i_ + γTV_i_ + u_j_ + ε_ij_where u_j_ ~ N(0, τ^2^) (patient random intercept),ε_ij_ ~ N(0, σ^2^),sample_type coded as ordered factor {normal < tumour}6. If model fails to converge, skip protein j7. Extract from the tumour coefficient:


β^, SE, t, df, p


8. Compute bias-corrected effect size:


$${g}_{\mathrm{adj}}={\rm{J}}(\mathrm{df})\times (\hat{\beta }/\hat{\sigma })$$ *▷* Hedges’ *g*


ENDFOR9. Apply Benjamini–Hochberg FDR correction to {p_j_} within c → {q_j_}10. Append {c, j, g_adj_, q_j_} to RENDFOR


### Consensus clustering analysis

The pan-cancer data were extracted from the corrected protein matrix, and subsequently preprocessed by selecting proteins whose CV values were larger than the median across all samples. Consensus clustering was then performed using the ConsensusClusterPlus^86^ package (v.1.67.0) with the *k*-means algorithm (*k* = 2–20) based on Euclidean distance. Silhouette analysis was applied to remove samples with silhouette widths less than 0. The remaining ‘core’ samples were subsequently used for differential protein expression analysis for each cluster versus all others using the Wilcoxon rank-sum test, which identified significantly upregulated and downregulated proteins (|log_2_ fold change| ≥ 1.5, *P*_adj_ < 0.05). Cluster-enriched proteins were also computed using the tissue specificity classification algorithm. The final protein signature set was derived from either significant proteins or cluster-enriched proteins, and was used for single-sample gene set enrichment analysis (ssGSEA^87^) against the MSigDB Hallmark gene sets^88^ using the GSVA^89^ package (v.1.51.1). The resulting pathway enrichment scores were statistically compared between the patient clusters using the limma framework.

### Tumour-enriched proteins

Tumour-enriched proteins were defined for each tumour type as proteins significantly upregulated in the tumour (T) compared with both paired non-tumour (NT) and a comprehensive panel of normal (healthy) tissues (N) from diverse organs. The T–NT comparisons were taken from the ‘Differential expression analysis’ section in the Methods. The T–N differences were computed using a linear mixed model similar to the T–NT analysis, but including only the sample collection batch as an additional fixed covariate, because the corrected matrix was used here. To ensure stringent tumour enrichment, the maximum intensity across all of the N samples was required to be lower than the 25th percentile intensity in the T samples, and the highest median intensity across the N samples was required to be less than half of the median intensity in the T samples.

### Locally enriched DEPs

The list of tumour-specific DEPs was mapped to tissue-enriched proteins from normal samples. Locally enriched DEPs were defined as the intersection of them.

### External database cross-referencing

The list of cancer dysregulated proteins was mapped to the DrugBank online database^90^ to find out potential targeted drugs, and the cancer–target–drug combinations were used to query clinical trials information from the ClinicalTrials.gov online database (https://clinicaltrials.gov). We downloaded 8,179 DrugBank records (6,653 targets; 7 February 2023), filtering for 528 biotech drugs targeting 573 proteins. Among these targets, 301 were quantified in our paired tumour dysregulation analysis, 260 of which were DEPs. By matching these drugs to ClinicalTrials.gov records for cancer types in which their targets were upregulated, we identified 36 drugs linked to 2,084 trials involving 77 upregulated DEPs across 10 cancer types (Supplementary Information). All matched clinical trials were visualized in a circular scatter plot.

We used the pan-cancer proteomic map (ProCan-DepMapSanger) drug response and CRISPR–Cas9 gene-essentiality screens dataset^44^ as a validation. In the ProCan-DepMapSanger dataset, linear regression analysis was performed to test the associations between protein abundances and either drug sensitivity or CRISPR–Cas9 gene dependency. Calculated nc_fdr and nc_beta denote the FDR and the corresponding regression coefficient from models, respectively. We filtered the protein–drug or protein–knocked gene associations of nc_fdr <0.01 and nc_beta < −0.1. Meanwhile, we selected the association pairs where the protein functions as the target of the drug or corresponds to the CRISPR perturbed gene (PPI = TRUE). The gene names were transformed into Swiss-Prot protein IDs and mapped to our list of cancer dysregulated proteins. The beta without-transcriptomics covariate, Hedges’ *g* and cancer of each validated protein–drug relationship were visualized in a scatter plot.

The favourable prognostic, unfavourable prognostic and cancer-enriched genes, from 14 types of human cancer (head and neck cancer, thyroid cancer, lung cancer, liver cancer, testis cancer, prostate cancer, stomach cancer, colorectal cancer, breast cancer, endometrial cancer, ovarian cancer, cervical cancer, pancreatic cancer and renal cancer), were obtained from the Human Pathology Atlas^40^ (https://www.proteinatlas.org/humanproteome/cancer). Our list of dysregulated proteins in cancer was mapped to the above public datasets for the same cancer types and visualized using a Sankey diagram.

The list of cancer dysregulated proteins was also mapped to TCGA and CPTAC datasets by proteins and corresponding cancer types, with the results displayed as scatter plots.

### Parallel reaction monitoring validation

A total of 137 cancer-specific dysregulated proteins were selected from pan-cancer data acquired before the year 2022 for parallel reaction monitoring (PRM) validation. Each selected precursor has no modification and no missed cleavage, with the peptide length ranging from 8 to 20, identified using Skyline (v.21.1). In total, 56 peptide precursors from 47 proteins and 9 iRT peptide precursors were analysed. Among the 47 cancer-specific proteins, 10 were still classified as cancer-specific. Skyline^91^ was used to analyse and quantify the targeted PRM data before generating the peptide matrix. The peptide matrix was transformed into the protein matrix using the online ProteomeExpert Peptide2Protein tool^92^ by means of the top three precursor intensities and with log_2_ transformation and quantile normalization. Differential expression analysis was performed after filling missing values with 0.5 times the minimum value in the whole matrix. DEPs were identified as proteins with a B-H adjusted *P* < 0.05 (paired Wilcoxon rank-sum test) and an absolute log_2_ fold change of means ≥ 1. The cancer-enriched proteins and cancer-specific dysregulated proteins were selected using the methods described above. Validated proteins were visualized in box plots.

### Validation of protein detection

Peptides were synthesized for PANX3 (SLAHTAAEYMLSDALLPDR, LVQHMLK and YFEFPLLER) by Jietai Biotechnology (http://www.synpeptide.cn/). These peptides were redissolved in 98% water:2% ACN:0.1% FA (v/v/v), mixed and analysed by the same LC–MS method that was used in the library construction process. Tandem MS spectra of three PANX3 peptides were manually reviewed in DDA files of cochlea in FragPipe.

The data analysis mentioned above was performed in R (v.4.4.0).

### Development of a data resource website

The website was constructed using a standard three-tier web architecture comprising an HTML-based presentation layer, Java- and Python-based business logic layer and MySQL-based data persistence layer. The front-end interface communicates with back-end services via the HTTP protocol, with all structured data stored in optimized MySQL relational tables. User queries are processed through database matching operations followed by Python-based analytical processing before being returned to the presentation layer. For enhanced data visualization, DEP results are dynamically rendered using the ECharts JavaScript library (https://echarts.apache.org), enabling interactive graphical representations.

### Reporting summary

Further information on research design is available in the Nature Portfolio Reporting Summary linked to this article.

## Online content

Any methods, additional references, Nature Portfolio reporting summaries, source data, extended data, supplementary information, acknowledgements, peer review information; details of author contributions and competing interests; and statements of data and code availability are available at 10.1038/s41586-026-10660-y.

## Supplementary information

Supplementary Information This file contains full descriptions for Supplementary Tables 1–9 (tables supplied separately), Supplementary Results, including Supplementary Figs. 1–8 and Supplementary References.

Reporting Summary

Supplementary Table 1 Sample origin information.

Supplementary Table 2 DDA data identifications.

Supplementary Table 3 Proteome transformation in development and oncogenesis.

Supplementary Table 4 Tissue specificity.

Supplementary Table 5 Druggable tissue-enriched proteins information.

Supplementary Table 6 Differential expression analysis.

Supplementary Table 7 Cross-reference to external cancer datasets.

Supplementary Table 8 Cancer-specific proteins.

Supplementary Table 9 Cancer DEPs and associated pathways and drugs.

Peer Review File

Source Data Supplementary Figs. 1–6 and 8.

## Source data

Source Data Fig. 1

Source Data Fig. 2

Source Data Fig. 3

Source Data Fig. 4

Source Data Fig. 5

Source Data Extended Data Fig. 1

Source Data Extended Data Fig. 2

Source Data Extended Data Fig. 3

Source Data Extended Data Fig. 4

Source Data Extended Data Fig. 5

Source Data Extended Data Fig. 6

Source Data Extended Data Fig. 7

Source Data Extended Data Fig. 8

## Data Availability

All of the qualitative and quantitative data that were used for analysis in this paper are openly accessible and fully available. Owing to the limits on publication length, the proteome data presented here cover some selected tissues and carcinomas only. To better visualize and present the entire database, we have built a website (https://db.prottalks.com/) that supports both protein-centric and tissue-centric queries. Specifically, pairs of tissues of interest can be subjected to differential expression analysis online, and the DEPs are shown in an instant heat map. Raw data as well as corresponding metadata annotations following the Sample and Data Relationship Format (SDRF-Proteomics) standard have been deposited at the ProteomeXchange Consortium (https://proteomecentral.proteomexchange.org) through the iProX^93,94^ resource (PXD077178, IPX0003578000) and the PRIDE^95,96^ repository (PXD063370). Source data are provided with this paper.

## Code availability

The code used for bioinformatic analyses in this study is publicly available on GitHub at https://github.com/guomics-lab/TPHP.
