# Supplementary material for: Spatial distribution of the proteome in the human body and in cancers
Source: Nature. 2026 Jun 17;656(8126):227–36. doi: 10.1038/s41586-026-10660-y (PMC13442039; doi:10.1038/s41586-026-10660-y)
Supplement: Supplementary file 1 [file 41586_2026_10660_MOESM12_ESM.pdf]

nature portfolio

Peer Review File

Spatial distribution of the proteome in human body and cancers

Corresponding Author: Professor Tiannan Guo

Any redactions in this file are there to maintain patient confidentiality, the confidentiality of unpublished data, or to remove third-party material.

This file contains all reviewer reports in order by version, followed by all author rebuttals in order by version.

Version 0:

Reviewer comments:

Referee #1

(Remarks to the Author)
This manuscript describes the generation and analysis of pan-cancer proteomes using Data Independent Acquisition (DIA) approaches (using a timsTOF instrument). The manuscript includes a general description of the work performed and then, naturally, includes the analysis of some highlights of the obtained results.

I have some comments that I think could enhance the manuscript significantly.

1. Since this journal is targeting non-experts in cancer proteomics as well, I think there is the need to describe this study (e.g. its uniqueness) in the context of others that have been performed before, for instance the CPTAC efforts and others. How does this study compare to them in different aspects: e.g. types of cancer studied, number and type of samples included, analysis approaches, other technical details? There is some information included about comparing Differentially Expressed Proteins with CPTAC/TCGA studies, but I think that more comparisons, and the unique points of this study in comparison with previous ones should be highlighted. This would help to understand better the extra value of the work included in this manuscript.

2. Also there are some large-scale proteomics studies (quantitative profiling) performed using cell lines. At least some of them should be cited. It would also be useful to highlight from the results obtained, why transcriptomics data is not useful enough in the case of analogous studies, and which are the advantages that proteomics techniques offer in addition. A lot of the information is already there in the current version, but I think this should be made more clear.

3. In the context of the samples coming from healthy donors, the authors state that they come from older individuals. I understand this is not the main focus of the manuscript, but it would be good to see if there are some clear differences to other previously published human proteomes generated in baseline conditions using mass spectrometry. The Human Protein Atlas has been used for this purpose in the manuscript, but this is data generated using antibodies (not mass spectrometry).

4- In the context of the data analysis, since it is quite a large dataset (1,635 samples, 1,900 MS raw files), I think the authors should ideally do a search using an entrapment database. This is becoming common in large-scale DIA proteomics studies to control better the False Discovery Rate. This is also relevant since the authors analysed the data in four different batches, that were combined at a later stage.

5. Then, there are some technical information/details which are just missing, which makes comparison with previous results difficult. For instance:
- How many PSMs and peptide sequences are required for a good protein identification/quantification in the DIA analysis? Is one PSM enough? Are shared peptides considered at all? This is not explained.
- The same type of information about thresholds for reliable peptide sequences is also missing for the creation of the spectral library using DDA data (which is the basis for the DIA analysis).
- The authors make claims about detecting “missing” proteins (when compared with the Human Proteome Project, HPP), but

comparisons are unfair, considering that the HPP has strict requirements to consider a protein as detected (see HPP guidelines 3.0). I understand of course that analogous guidelines have not been developed for DIA approaches, but also considering the previous point in my review, which are the requirements that have been used here to consider a protein as detected? Of course it is good that the detection of three of the missing proteins was validated in some cases using synthetic reference peptides, but some key information is missing anyway.
- Detailed search parameters are also missing.

6. The website summarising access to the data should be more highlighted in the text (maybe even in the abstract), and also information about the technical details about how it was developed should be added to the Methods section. I think the resource contains data coming from different studies, not only this one, doesn't it?

7. I tried to access the raw data in iProX and was unable to. Maybe reviewer access needs to be enabled? Additionally, the spectra library should also be shared in the public domain as well, for reproducibility purposes.

8. Minor detail: It is ProteomicsDB and not ProteomeDB.

(Remarks on code availability)
I have reviewed the website that hosts the data. I have added a comment in my review.

Referee #2

(Remarks to the Author)
The authors present a study that aims to refine and expand initial resources of the human proteome using spatial protein expression information of various normal and cancer-associated human tissues by state-of-the-art MS-based proteomics. Furthermore, the data is used to derive information of diagnostic and therapeutic value in the field of oncology.

Overall, the study presents as an impressive effort in terms of sample collection and preparation, MS data acquisition, as well as comprehensive bioinformatic analysis and visualization thereof. Already existing drafts of the human proteome are recapitulated using DIA-MS bottom-up proteomics, but expanded towards a much broader range of human tissue samples. Notably, the presented data does not imply a substantial extension of the initial drafts regarding proteome coverage. This implies that conventional, untargeted shotgun proteomics has limitations in obtaining "full" coverage of the theoretical human proteome and should be discussed by the authors in the context of their hypotheses.

Taken together, the presented study provides a refined approach to draft the human proteome using state-of-the-art methods like DIA analysis and high-speed, high-sensitivity mass spectrometry. Although it represents an important resource, it remains in its present form mainly descriptive and novel aspects, regarding both technology and biological insight, are insufficiently validated.

Major points:

1. The presented sampling and analysis strategy is based on well-established workflows for DIA proteomics and is conducted in a reasonable manner. However, some of the methodological aspects demand further clarification. Regarding sample collection it would be interesting how tissue types and borders were defined within and between organs. This might explain counter-intuitive proteome coverages found in specific tissues, for example cartilage and hair, which are very close to more complex tissues.

2. Furthermore, the rationale for the MS data acquisition strategy should be explained in more detail, i.e. i) why MS1 quantitative information was not already used from the DDA dataset and ii) what is the study-specific benefit of constructing the massive spectral library, while common DIA processing engines (including DIA-NN) are capable to perform comprehensive library-free searches.

3. In this regard, the common thread regarding sample preparation, replication and data standardization remains elusive. As the authors state in the last paragraph of the main text, different sample preparation methods were used for subsets of tissues, inevitably raising the questions for batch effects and quantitative integrity. To account for that, in Fig. S2f the authors nicely show that DIA samples cluster based on their pathophysiological profile. However, compared to Fig. S2e this pattern strongly correlates with another variable, which could be either the month of acquisition (actual figure) or the batch of "glass capillary" that was used (figure legend). These inconsistencies should be corrected and discussed in their intended context to enable proper assessment by the reader.

4. The measures taken throughout the study for sample replication and randomization should be clarified, as well as the inclusion of a "sample pool", which is mentioned, but not explained regarding composition, sampling strategy and data processing.

5. Besides these open methodological questions, in Fig. 2 the authors nicely show the somehow expected observation that different tissue states can be tracked based on their proteome profile. To tease out differences they focus on the distinct behaviour of brain samples; However, such a sub-analysis may also be interesting for other tissue types. This would strengthen the uniqueness of the "brain profile" and otherwise help to rule out batch effects, e.g. based on sequential analysis of samples from the same specific tissue during a given period of time.

6. Beyond general sample classification the authors touch on their approach to exploit the dataset to inform diagnosis and therapy of various cancers. This has high potential for innovation; however, the main limitation is the low number of individual patients for each of the analyzed cancer entities. A growing body of evidence suggests that inter- and even intra-individual heterogeneity is critical to inform such studies for precision medicine apart from the obtained pan-cancer motifs. This is not addressed in the study. Hence, the clinical value for both diagnostics and cancer therapy is limited.

7. Some proteins and protein signatures are put in biological/clinical context by the authors. However, none is experimentally validated leaving the present study purely descriptive.

8. The authors should analyze interpatient variability within the individual cancer entities and should investigate whether they can identify patterns of biological/ clinical relevance beyond known ones.

Bioinformatics:

The authors employ a state-of-the-art computational pipeline for facilitating a proteomic mapping across many tissue types and cancer samples. However, I have some concerns and several aspects of the bioinformatics workflow — ranging from data pre-processing to downstream statistical analysis — should be more rigorously explained and clearly justified.

1. My main concern regards the inconsistent and sometimes arbitrary choice of parameters in different parts of the pipeline. Please justify choice and use consistent values throughout the analysis in the following aspects: Different imputation methods are used in distinct parts of the analysis. For example, the authors mention filling missing values with 0.5 times the minimal value in the whole matrix in the tissue specific context while also using random numbers drawn from a normal distribution scaled by a minimal value in the matrix in the pan-cancer analysis. How robust are the downstream analyses when instead using state-of-the-art imputation algorithms such as the dreamAI algorithm (Weiping et al., DreamAI: algorithm for the imputation of proteomics data. Biorxiv, 2020, S. 2020.07. 21.214205)?

2. Similarly, inconsistent thresholds for fold change were chosen, with 1.5 for the paired tumor/normal analysis and 2 for the drug analysis.

3. The choice of perplexity for t-SNE as (n-1)/3-1 seems unconventional; could you motivate this? How robust was the analysis wrt to this choice?

4. I wonder how robust the hierarchical clustering for pancancer analysis is; in proteomics analyses, correlation-based metrics can sometimes capture relationships more robustly. In addition, does the structure hold when performing a consensus clustering?

5. For the batch-effect correction, more quantitative evaluations of the batch-effect removal (e.g., before–after metrics/plots, clustering statistics) would be helpful; also, in the downstream analysis, were batch variables included in limma?

6. For the integration with external data (mapping to drug targets), could you clarify how mismatches in protein identifiers, isoforms, or quantification differences were handled?

Finally, for reproducibility, the entire computational workflow should be made accessible through open code and well-annotated analysis pipelines.

Minor comments:

• The abbreviations introduced in Fig. 1 are difficult to track in the following figures. It may be beneficial to use the full descriptions instead.

• In Fig. 2b the color-coded tissue class does not appear in the actual graph.

• The section about brain development requires references (line 149 and following).

• The tissue classification metrics should be explained more in detail (line 169 and following).

• In Fig. S1 the legend does not fit to the actual illustrations.

• In Fig. S2b the color-coding does not fit, which complicates access to the illustration. Overall, the figure looks quite busy for the actual content.

• For Fig. S4a the shown graph does not fit to the main text (line 192). Furthermore, the scope of the shown analysis requires more detailed explanation as well as suitable citations.

• The comparison opened in line 214 should be explained more in detail including citations.

• In line 229 and following the correct protein name would be “BGLAP”.

(Remarks on code availability)
The entire computational workflow should be made accessible through open code and well-annotated analysis pipelines.

Referee #3

(Remarks to the Author)
The manuscript, "Spatial distribution of the proteome in human body and cancers", presents a valuable and comprehensive proteomic resource, profiling 251 human tissues and 25 carcinoma types. The data have strong potential to support research in tissue specificity, cancer biomarker discovery, and drug target investigation. However, while the dataset itself is impressive in scope, the current version of the manuscript falls short in terms of data interpretation, clarity, and depth of analysis.

Major Comments

1. Data visualization and interpretability

Several figures are difficult to interpret, particularly in terms of understanding the quality and structure of the data. For instance, in Fig. 2a (t-SNE), the brain grouping is emphasized, but other apparent groupings are not annotated or explored. Adding additional coloring schemes (e.g., by tissue type or anatomical group) could enhance the readability and interpretability of these dimensionality reduction plots. Similarly, Fig. 3g does not clearly convey clear data patterns in its current form.

2. Limited discussion on data quality and deviations

While the authors acknowledge some heterogeneity or unexpected patterns (e.g. in tissues such as vagina, eye, cartilage, fallopian tube, and pancreas, as well as clustering involving peripheral nerve & fat, uterus & ureter, throat & salivary gland), these are not sufficiently discussed. Providing deeper insights into these deviations would increase the transparency and credibility of the dataset.

3. Validation strategy

The rationale and outcome of the PRM validation (14 out of 315 cancer-specific proteins) are unclear. The authors should clarify the selection criteria for these proteins, the success rate of validation, and how these results reflect the overall reliability of the cancer-specific findings.

4. Transcriptomic vs. proteomic specificity

It would be valuable to discuss how the observed protein specificity categories compare to transcriptomic data for overlapping tissues.

5. Biological insights and cancer findings

Many of the most compelling biological findings, particularly those from the paired carcinoma-adjacent analyses, are included in the supplementary materials. These results could significantly enhance the manuscript and deserve to be highlighted in the main figures. In contrast, the drug target investigation section receives considerable attention but remains largely conceptual or anecdotal. Additionally, mapping the cancer-specific proteins to the protein specificity categories would be valuable for further biological insights.

Version 1:

Reviewer comments:

Referee #1

(Remarks to the Author)
This revised version of the manuscript addresses adequately the major points mentioned in my previous review. As key points, the methodology and the uniqueness of this study (when compared with previous ones) are much clearer. Overall, the information that the manuscript contains is extensive, and naturally, the authors can only show some highlights from all the generated data.

For this reason, I think the authors should improve the reusability of such valuable dataset for the community, since I anticipate that this dataset will be heavily reused in the future. For this purpose, I suggest that the authors provide an accurate metadata annotation of the dataset, by showing the links between the raw files and the samples (for instance, by creating a SDRF-Proteomics file, or several ones, and making it available in the dataset submitted to ProteomeXchange/PRIDE).

Also, the tissues under study do not seem to be properly annotated in the dataset (as everything is annotated as ‘whole body’, which is not correct). Furthermore, the submitted dataset does not seem to contain (at least as far as I could see) the

spectral library used for the analysis and the corresponding DDA generated data for constructing the PASEF spectral library. I only could see DIA and PRM related data. These data should be provided e.g. in a different linked dataset, and would enable the reproducibility of the reported results. Just for clarification, I accessed the dataset via PRIDE as a reviewer.

An extra detail is that neXtProt is mentioned as an active database, but it is no longer active, and the latest release was done in September 2023 (see https://www.expasy.org/archives/nextprot). UniProtKB/SwissProt is the best alternative, and any claims on missing proteins should be confirmed and attributed to UniProt.

Referee #2

(Remarks to the Author)
The authors have sufficiently addressed my comments.

Open Access This Peer Review File is licensed under a Creative Commons Attribution 4.0 International License, which permits use, sharing, adaptation, distribution and reproduction in any medium or format, as long as you give appropriate credit to the original author(s) and the source, provide a link to the Creative Commons license, and indicate if changes were made.
In cases where reviewers are anonymous, credit should be given to 'Anonymous Referee' and the source.
The images or other third party material in this Peer Review File are included in the article's Creative Commons license, unless indicated otherwise in a credit line to the material. If material is not included in the article's Creative Commons license and your intended use is not permitted by statutory regulation or exceeds the permitted use, you will need to obtain permission directly from the copyright holder.
To view a copy of this license, visit https://creativecommons.org/licenses/by/4.0/

Referee #1 (proteomics)

Remarks to the Author:

This manuscript describes the generation and analysis of pan-cancer proteomes using Data Independent Acquisition (DIA) approaches (using a timsTOF instrument). The manuscript includes a general description of the work performed and then, naturally, includes the analysis of some highlights of the obtained results.

I have some comments that I think could enhance the manuscript significantly.

1. Since this journal is targeting non-experts in cancer proteomics as well, I think there is the need to describe this study (e.g. its uniqueness) in the context of others that have been performed before, for instance the CPTAC efforts and others. How this study compares to them in different aspects: e.g. types of cancer studied, number and type of samples included, analysis approaches, other technical details? There is some information included about comparing Differentially Expressed Proteins with CPTAC/TCGA studies, but I think that more comparisons, and the unique points of this study in comparison with previous ones should be highlighted. This would help to understand better the extra value of the work included in this manuscript.

Reply: We agree and have substantially revised the manuscript to better situate our work within existing cancer proteomics efforts and clarify its unique contributions, as detailed below.

1, The comparative analysis of our pan-cancer cohort versus TCGA and CPTAC—detailing differences in cancer types, sample types, and patient numbers—has been integrated into the "Collection of tissue samples" subsection within the Materials and Methods section of the Supplemental Information (SI).

"…We systematically compared the cancer types, number, and types of samples of our pan-cancer cohort with those in TCGA[1] and CPTAC[2]. According to The NCI Genomic Data Commons[3], our study includes two rare malignancies not covered by either TCGA or CPTAC: gastrointestinal stromal tumors (GIST) and fallopian tube carcinoma. Conversely, TCGA and CPTAC include several cancer types absent in our current cohort, including adrenal cortical carcinoma, bladder cancer, leukemia, mesothelioma, skin cancers, and melanoma. Regarding sample size, our study profiles approximately 40 patients per cancer type (Table S1), while TCGA typically includes ~200 cases per type and CPTAC ~160. With respect to normal control tissues, TCGA primarily uses blood samples for germline genomic analysis, whereas CPTAC often includes matched adjacent non-tumor tissues (either fully or partially paired). In our pan-cancer cohort, we incorporated matched adjacent non-tumor tissues for most patients, allowing tumor-non-tumor comparisons at the proteome level…"

More details are provided in the Table R1 only for reviewer.

Besides, we have stressed these differences in the Introduction:

"…While consortia like TCGA, ICGC, and CPTAC have generated extensive multi-omics datasets for specific tumors[15,16], challenges in cross-tumor type comparisons limit the insight into differences among cancers that can be obtained from these resources[17]. Comprehensive proteome profiling across diverse human tissues and states requires broad tissue coverage and in-depth, high-throughput proteomics, a need effectively addressed by DIA-MS[18-20]. Leveraging DIA-MS, we present a rich data resource detailing the spatial distribution of 13,609 proteins. This coverage includes 58 normal adult tissues, paired tumor and non-tumor samples from 25 cancer types, and 22 fetal tissue types, encompassing nearly all solid human tissues, body fluids, and major cancer types (Figure 1, Extended Data Figure 1A) …"

2, Methodological details: We deposited the information of the analysis approaches and other technical details in the Supplementary Information file. Briefly, our study employs a data-independent acquisition (DIA)–based proteomics approach for protein quantification, which offers several advantages over the

tandem mass tag (TMT) and data dependent acquisition (DDA)–based strategy used in most CPTAC studies. Specifically, DIA provides a high degree of quantitative reproducibility which enables reliable cross-cohort, and cross-study quantitative comparison, and better scalability for large clinical cohorts. Additionally, DIA allows retrospective data mining as spectral libraries and search methods improve, enhancing long-term data reusability. While TMT enables multiplexing of up to 35 samples per run, it may suffer from ratio compression and requires common control samples for multi-batch data integration. The DIA workflow avoids these limitations and supports more robust cross-study quantitative comparisons and extensible sample collection—making it particularly suitable for building a reliable, open-access proteomic resource for the broader research community.

We have highlighted these methodological strengths in the revised main text.

In Introduction section:

"Comprehensive proteome profiling across diverse human tissues and states requires broad tissue coverage and in-depth, high-throughput proteomics, a need effectively addressed by DIA-MS[18-20]. Leveraging DIA-MS, we present a rich data resource detailing the spatial distribution of 13,609 proteins…"

In Results section:

"…Paired tumor and non-tumor samples were used to generate a pan-cancer proteomic landscape (Figure S3A), representing the first pan-cancer proteomic dataset using a unified pipeline and platform, with batch design minimizing multi-batch effects[36]…"

3, Unlike CPTAC/TCGA pan-cancer studies that harmonize datasets from multiple single-cancer projects (potentially reducing inter-cancer differences), our approach maintains cancer-specific signals while enabling direct comparison.

We characterized tumor-specific proteomic signatures, including dysregulated proteins unique to the tumor and the tissue-enriched proteins dysregulated within the corresponding primary tumor (revised Figure 6). These signatures strongly correlated with the cell of origin, tissue developmental pathways, and impaired tissue-specific functions, offering a reasonable explanation for the observed distinct tissue-specific trajectory patterns. Crucially, the significantly higher intensity of these tumor-enriched proteins, compared to both paired non-tumor tissue and all normal samples, suggests a strong therapeutic targeting potential with minimal predicted off-target side effects, a conclusion supported by our comprehensive baseline comparisons. These findings highlight the critical importance of these cancer-specific proteomic signatures for both elucidating disease mechanisms and developing effective, targeted therapeutic strategies.

2. Also there are some large-scale proteomics studies (quantitative profiling) performed using cell lines. At least some of them should be cited. It would also be useful to highlight from the results obtained, why transcriptomics data is not useful enough in the case of analogous studies, and which are the advantages that proteomics techniques offer in addition? A lot of the information is already there in the current version, but I think this should be made more clear.

Reply: We further clarify the limitations of transcriptomics and the unique advantages of proteomics in functional and translational research.

1, In the pan-cancer section, we have already cited and integrated the pan-cancer proteomic atlas of 949 cell lines[4], which systematically linked protein intensity to drug response and CRISPR KO cell wellness as excerpted below.

"...we mapped our data to ProCan-DepMapSanger proteomic map[59]. By correlating the proteomic profiles of the cell lines with their drug response data and CRISPR-Cas9 gene essentiality data in 949 cell lines using linear regression, ..."

2, We also cite the CCLE proteome[5] (375 cell lines, TMT-MS). This resource quantitatively profiled thousands of proteins across 375 diverse cell lines by mass spectrometry, along with DNA and RNA information, identified poorly correlated pathways between RNA and protein in diverse cell lines. We have identified similar discordant pathways in the tissue level as discussed in SI.

"...Multiple cross-tissue studies have shown that RNA–protein correlations across tissues are only moderate[6], and our data align with this trend, showing median Pearson correlations of 0.25 (HPA[7]) and 0.24 (GTEx[8]). Poorly RNA-correlated proteins mainly enriched in post-transcriptional regulation, including translational efficiency, protein complex and RNA splicing, and axonal transport (SI, Table S3), which is consistent with a previous study[5] ..."

3, Proteomic analysis offers unique advantages in biological and clinical settings where transcriptomic data may be limited. For instance, RNA is often unstable and requires fresh or rapidly preserved tissues for reliable measurement, whereas proteins are more stable and can be robustly analyzed in a wider range of sample types. This makes proteomics particularly valuable for studying formalin-fixed paraffin-embedded (FFPE) tissues, which are routinely collected in clinical practice and allow precise anatomical and histopathological annotation. Recent advances have demonstrated that FFPE samples are increasingly compatible with mass spectrometry–based proteomics[9,10], enabling retrospective studies on large, well-annotated patient cohorts. Moreover, in non-cellular or acellular biospecimens such as blood plasma, urine, hair, and nails, proteins—not mRNAs—are the primary stable molecular carriers of biological function and environmental exposure history. These matrices are often inaccessible to transcriptomic profiling but are highly relevant for biomarker discovery and monitoring. Critically, proteomics provides unique and irreplaceable insights into pathway activity at the effector level, protein complex homeostasis, secretion axes, and clinically relevant sample types, beyond what transcriptomics alone can capture. Thus, proteomics delivers complementary and irreplaceable information beyond transcriptomics, especially in clinically relevant contexts and diverse biospecimen types.

Revised text in Introduction:

"...messenger RNA (mRNA) abundance correlates only moderately with the expression of proteins, which are the major functional and druggable molecules..."

3- In the context of the samples coming from healthy donors, the authors state that they come from older individuals. I understand this is not the main focus of the manuscript, but it would be good to see if there are some clear differences to other previously published human proteomes generated in baseline conditions using mass spectrometry. The Human Protein Atlas has been used for this purpose in the manuscript, but this is data generated using antibodies (not mass spectrometry).

Reply: Thank you for this careful reminder. In response, we have made the following revisions.

1, We have noted the age distribution of the tissue donors as a limitation in the Conclusion:

"...Normal samples are predominantly from elderly donors, which may affect tissues such as the mammary gland due to involution..."

In addition, we evaluated Pearson correlations for overlapping tissues and proteins with previous human proteome studies with age information[11,12], as detailed in the SI section.

"…We evaluated Pearson correlations for overlapping tissues and proteins among established human proteome studies with age information[11,12]. Seven tissues overlapping with the previous human draft exhibited moderate correlations (~0.5) of the overlapping 8398 proteins, indicating reasonable concordance despite methodological differences (Extended Data Figure 10, SI). GTEx age-matched comparisons[11] did not substantially improve tissue expression correlations. Notably, pancreas exhibited minimal correlation, probably due to post-mortem autolysis…"

2, Compared to previous proteome drafts, we have 29 major tissue types only in our data, leading to refined tissue specificity classification and reflecting broader tissue representation. As documented in Table S4, we performed comprehensive comparisons with GTEx, HPA (based on RNA), and the dataset from Wang et al.[13] as excerpted below:

"…Of the 1716 tissue-enriched proteins identified, 749 were previously reported as enriched in the corresponding tissue types in published human proteome[13,14] or transcriptome datasets[8]. Of these, 666 were supported at the protein level and 426 showed concordant enrichment in HPA RNA-seq data. Across 36 tissues overlapping with the HPA, we identified 831 proteins uniquely enriched in our dataset and 122 exclusively enriched in the HPA RNA data. Notably, 480 tissue-enriched proteins were identified in 24 tissue types underrepresented in prior studies, underscoring the expanded tissue coverage. Among these, we identified PANX3 as the top cochlea-enriched protein (Table S3), a finding of particular significance given that PANX3 is classified as a "missing protein" in neXtProt[27] (PE2, Extended Data Figure 2B). We further synthesized two unique peptides (LVQHMLK and YFEFPLLER) from PANX3 to confirm its presence and cochlear-specific expression (Extended Data Figure 2C) …"

3, Compared to existing human proteome drafts, our dataset provides significantly higher sub-tissue granularity (212 newly added, Extended Data Figure 1A), enabling detailed characterization of tissue and organ heterogeneity. Additionally, our comprehensive inclusion of fetal, cancer, and tumor-adjacent samples extend the biological scope beyond previous mass spectrometry-based human proteome studies.

4- In the context of the data analysis, since it is quite a large dataset (1,635 samples, 1,900 MS raw files), I think the authors should ideally do a search using an entrapment database. This is becoming common in large-scale DIA proteomics studies to control better the False Discovery Rate. This is also relevant since the authors analysed the data in four different batches, that were combined at a later stage.

Reply: We thank the reviewer for raising this important and constructive point. We would like to clarify that the different batches in our study were processed jointly in DIA-NN in a single run using the existing .quant files to generate a unified matrix. In response to the reviewer's comment, we have reprocessed all data together in a single analysis and implemented a combined entrapment strategy[14]. We have now explicitly emphasized these in the revised Methods section.

Main text:

"…To ensure the quality of large-scale proteomic data[21], we searched the DIA-MS raw data against a combined spectral library that integrated the human spectra with entrapment spectra from non-human species[22]…"

SI:

"… To rigorously assess and control false discoveries, we implemented a combined entrapment strategy by constructing a combined spectral library that contained empirical human-derived spectra from the project-specific DDA library together with decoy spectra generated from non-human species[15]. This design enabled a direct evaluation of identification specificity at both the peptide and protein group levels. Entrapment PSM and precursor were detected at a low level, representing less than 1% of the

False Discovery Proportion (FDP) at a 1% q-value cutoff. However, the FDP exceeded 2% at the protein level. To control the protein FDR to less than 2%, we subsequently adjusted the cutoff to 0.1% (Extended Data Figure 3) ...”

“...This design produced the desired ratio of entrapment-to-original target entries at both levels: 16.714 for protein groups (256,259 entrapment vs. 15,332 targets) and 1.152 for precursors (739,318 entrapment vs. 689,568 targets) ...”

...To select suitable q-value thresholds, we evaluated precursor and global protein-group q-value cutoffs across the range 1×10^{-4} to 0.01, while keeping the run-specific protein-group q-value fixed at 0.01. To balance sensitivity and confidence, we finally adopted the following criteria: Proteotypic = 1, Q.Value < 0.001, PG.Q.Value < 0.01, Global.Q.Value < 0.001, and Global.PG.Q.Value < 0.001. These settings ensured tight global control of the false discovery proportion across all reporting levels in our multi-batch DIA dataset...”

5- Then, there are some technical information/details which is just missing, and then it makes comparison with previous results difficult. For instance:

- How many PSMs and peptide sequences are required for a good protein identification/quantification in the DIA analysis? One PSM is enough? Are shared peptides considered at all? This is not explained.

Reply: Thank you for this detailed question. In DIA workflows, the fundamental scored evidence is a precursor-peak match (candidate chromatographic elution peak with co-eluting fragment-ion traces) rather than a DDA-style PSM[16]. Besides, there is no universally defined threshold for PSM counts to establish confident protein identification in DIA. Instead, confidence is determined by the quality of the chromatographic and spectral evidence across multiple fragments, combined with library matching, retention time prediction, and statistical scoring at the precursor and protein levels via tools like DIA-NN[17].

We have expanded the Methods section in the SI to address these concerns for shared peptides.

“... To increase stringency, we retained only proteins supported by unique peptides (i.e., protein groups without “indistinguished” members in the FragPipe-annotated DDA library) and only proteotypic peptides (Proteotypic = 1 in the DIA-NN report) ...”

- The same type of information about thresholds for reliable peptide sequences is also missing for the creation of the spectral library using DDA data (which is the basis for the DIA analysis).

Reply: For DDA data, we have added to the SI in Database searching and library generation of the Methods part.

“...Thus, each peptide was identified either as a unique peptide to a specific protein (or protein group) or as a razor peptide to the protein (or protein group) with the most peptide evidence. Correspondingly, each protein (or protein group) was identified by either a unique peptide or a razor peptide detected for it. In downstream DIA analysis, protein groups containing indistinguishable proteins are excluded from the quantification reports...”

- The authors make claims about detecting “missing” proteins (when compared with the Human Proteome Project, HPP), but comparisons are unfair, considering that the HPP has strict requirements to consider a protein as detected (see HPP guidelines 3.0). I understand of course that analogous guidelines have not

been developed for DIA approaches, but also considering the previous point in my review, which are the requirements that have been used here for consider a protein as detected? Of course it is good that the detection of three of the missing proteins was validated in some cases using synthetic reference peptides, but some key information is missing anyway.

Reply: We thank the reviewer for the careful assessment of our methods.

We have further detailed the criteria for missing proteins in the SI.

"...We applied the criteria in line with the Human Proteome Project (HPP) Guidelines 3.0 to the DDA data to ensure high-confidence identifications of missing proteins. Specifically, we controlled the false discovery rate (FDR) at multiple levels, including peptide-spectrum match (PSM), precursor, and peptide levels, typically maintaining a threshold of 1%. Proteins were considered confidently identified only if supported by at least two unique, proteotypic peptides, each comprising a minimum of nine amino acids. In the context of missing protein detection, we focused on candidates annotated as protein existence (PE) level 2 to 4 in the neXtProt database (release: January 20, 2024) ..."

For DIA-based identification, we applied stringent criteria using a combined entrapment spectral library and rigorous multi-level filtering as excerpted below.

Main text:

"...A total of 13,609 proteins were quantified across 3005 MS files, with a global false discovery rate (FDR) of 0.1% at protein level (Figure 1, S2A, Extended Data Figure 3, SI) ..."

SI:

"...To rigorously assess and control false discoveries, we implemented a combined entrapment strategy by constructing a combined spectral library that contained empirical human-derived spectra from the project-specific DDA library together with decoy spectra generated from non-human species[15]. This design enabled a direct evaluation of identification specificity at both the peptide and protein group levels. Entrapment PSM and precursor were detected at a low level, representing less than 1% of the False Discovery Proportion (FDP) at a 1% q-value cutoff. However, the FDP exceeded 2% at the protein level. To control the protein FDR to less than 2%, we subsequently adjusted the cutoff to 0.1% (Extended Data Figure 3) ..."

...To select suitable q-value thresholds, we evaluated precursor and global protein-group q-value cutoffs across the range 1×10^{-4} to 0.01, while keeping the run-specific protein-group q-value fixed at 0.01. To balance sensitivity and confidence, we finally adopted the following criteria: Proteotypic = 1, Q.Value < 0.001, PG.Q.Value < 0.01, Global.Q.Value < 0.001, and Global.PG.Q.Value < 0.001. These settings ensured tight global control of the false discovery proportion across all reporting levels in our multi-batch DIA dataset..."

- Detailed search parameters are also missing.

Reply: We thank the reviewer for emphasizing methodological transparency. All parameter configuration files (.txt) are deposited with raw mass spectrometry data in iProX and PRIDE. Temporary iProX links are provided below in the answer for point 7 for reviewer access.

Methodological Description in Materials and Methods Section of SI:

For DDA Search:

"*...The database searching of raw files was performed by the MSFragger search engine, referring to a UniProtKB/Swiss-Prot Homo sapiens (UP000005640) database (containing 20,377 canonical protein*

sequences; downloaded on May 7th, 2020), appended with reverse protein sequences as decoys generated by the built-in Philosopher decoys generation tool. Then, Philosopher was utilized for further evaluation, protein inference, and 2D FDR filtering with a threshold of 0.01; the picked FDR algorithm is used to scale FDRs for large datasets. The built-in library generation script gen_con_spec_lib.py was utilized to build consensus PASEF spectral libraries with 0.01 FDR control on both peptide-level and protein-level. Most of the parameters were maintained as reported in the prior report[45], except for the application of semi-enzymatic tryptic digestion rule, 0.05 Daltons of fragment mass tolerance, enabled razor algorithm, and spiked iRT based linearly RT alignment...."

For DIA search:

"... DIA searching was performed by DIA-NN (version 2.2.0 Academia) with the FragPipe-generated spectral library with its existing protein inference results[45]. Both MS1 and MS2 mass accuracy were set as 15 ppm, and a Robust LC (high precision) quantification strategy and RT-dependent cross-run normalization were chosen for quantitative results. Moreover, the different runs were treated as unrelated with MBR disabled ... we finally adopted the following criteria: Proteotypic = 1, Q.Value < 0.001, PG.Q.Value < 0.01, Global.Q.Value < 0.001, and Global.PG.Q.Value < 0.001. These settings ensured tight global control of the false discovery proportion across all reporting levels in our multi-batch DIA dataset ..."

6- The website summarising access to the data should be more highlighted in the text (maybe even in the abstract), and also information about the technical details about how it was developed should be added to the Methods section. I think the resource contains data coming from different studies, not only this one, doesn't it?

Reply: We thank the reviewer for this suggestion. We have revised the manuscript as follows:

1, Added the data access website link in the main text.

2, Included technical details about the website's development in the Supplementary Information (Materials and Methods - Development of data resource website).

"...The website was constructed using a standard three-tier web architecture comprising an HTML-based presentation layer, Java/Python-based business logic layer, and MySQL-based data persistence layer. The frontend interface communicates with backend services via the HTTP protocol, with all structured data stored in optimized MySQL relational tables. User queries are processed through database matching operations followed by Python-based analytical processing before being returned to the presentation layer. For enhanced data visualization, differentially expressed protein results are dynamically rendered using the ECharts JavaScript library (https://echarts.apache.org), enabling interactive graphical representations ..."

3, As noted, the resource integrates data from multiple studies (including this work).

7- I tried to access the raw data in iProX and I was unable to. Maybe reviewer access needs to be enabled? Additionally, the spectra library should also be shared in the public domain as well, for reproducibility purposes.

Reply: We sincerely appreciate the reviewer's diligence in verifying data accessibility and apologize for the initial access issues. We have taken the following steps to ensure full transparency and reproducibility:

1, Temporary iProX links are provided below for reviewer access. These links are valid for 360 days, from March 26, 2025, to March 21, 2026.

DDA: https://www.iprox.cn/page/DSV021.html;?url=1742978458830yNqh (Password: Ntrd)

DIA: https://www.iprox.cn/page/DSV021.html;?url=1742978556591ZyK6 (Password: M1qd)

PRM: https://www.iprox.cn/page/DSV021.html;?url=1742978596090SrRP (Password: upTV)

2, Besides, all raw data are now available through the PRIDE repository with reviewer-specific access:

Project accession: PXD063370

Token: 6ShvG10D5X4i

Alternatively, reviewer can access the dataset by logging in to the PRIDE website using the following account details:

Username: reviewer_pxd063370@ebi.ac.uk

Password: Ebbaz7FL3Drh

And we have changed the description in Data Accessibility in the Manuscript:

"Raw data are available for download at https://www.iprox.org/ (IPX0003578000) and https://www.proteomexchange.org/ (PXD063370)."

3, We have uploaded the spectral library along with the raw data both in iProX and PRIDE with DDA data.

Following publication, the complete dataset will be publicly deposited with a persistent identifier, superseding current restricted access.

8- Minor detail:

- It is ProteomicsDB and not ProteomeDB.

Reply: We sincerely appreciate the reviewer's meticulous attention to nomenclature accuracy. We reviewed all text and figures and corrected every instance.

Referee #2 (proteomics, cancer, clinical)

Remarks to the Author :

The authors present a study that aims to refine and expand initial resources of the human proteome using spatial protein expression information of various normal and cancer-associated human tissues by state-of-the-art MS-based proteomics. Furthermore, the data is used to derive information of diagnostic and therapeutic value in the field of oncology.

Overall, the study presents as an impressive effort in terms of sample collection and preparation, MS data acquisition, as well as comprehensive bioinformatic analysis and visualization thereof. Already existing drafts of the human proteome are recapitulated using DIA-MS bottom-up proteomics, but expanded towards a much broader range of human tissue samples. Notably, the presented data does not imply a substantial extension of the initial drafts regarding proteome coverage. This implies that conventional, untargeted shotgun proteomics has limitations in obtaining "full" coverage of the theoretical human proteome and should be discussed by the authors in the context of their hypotheses.

Taken together, the presented study provides a refined approach to draft the human proteome using state-of-the-art methods like DIA analysis and high-speed, high-sensitivity mass spectrometry. Although it represents an important resource, it remains in its present form mainly descriptive and novel aspects, regarding both technology and biological insight, are insufficiently validated.

Major points:

1. The presented sampling and analysis strategy is based on well-established workflows for DIA proteomics and conducted in a reasonable manner. However, some of the methodological aspects demand further clarification. Regarding sample collection it would be interesting how tissue types and borders were defined within and between organs. This might explain counter-intuitive proteome coverages found in specific tissues, for example cartilage and hair, which are very close to more complex tissues.

Reply: We thank the reviewer for the careful assessment of our data. We have made the following revisions.

1, We added more technical details in the Methods part of SI as copied below:

"…To ensure anatomical accuracy, we used (i) macro-dissection by two experienced anatomists following a standardized protocol by two experienced anatomists guided by a standardized anatomical protocol listed in the author list and (ii) histopathological validation via H&E-stained FFPE sections (7-µm thickness) reviewed independently by two pathologists listed in author list for tissue types that are unsure to be clearly partitioned at the anatomical level…"

2, To evaluate whether the high number of protein identifications included low-confidence entries, we applied stricter false discovery rate (FDR) filtering using entrapment database analysis—a method that estimates false positives by detecting proteins in decoy or non-existent sequences (Figure S2A, S2B). This reduced protein and precursor identifications by approximately 10% (Table below), indicating a measurable contribution of spurious matches under standard criteria.

File name	Tissue type	PrecursorID of target-entrapment spectral library	ProteinID of target-entrapment spectral library	PrecursorID of target spectral library	ProteinID of target spectral library	PrecursorID reduction	ProteinID reduction
DIA_497	hair	20,638	3267	24,102	3770	-0.14	-0.13
DIA_494	hair	13,467	2413	16,180	2892	-0.17	-0.17
DIA_495	hair	28,088	3986	31,049	4432	-0.10	-0.10
DIA_496	hair	26,315	3634	28,496	4025	-0.08	-0.10

Furthermore, excluding proteins absent in more than half of hair samples reduced the dataset to 2,946 proteins. These missing-value-prone proteins are often low-abundance or inconsistently detected, and

their removal enhances dataset reliability. Overall, the hair proteome is dominated by highly abundant keratin and filament proteins (Extended Data Figure 5A).

In addition, we compared the hair proteome with previous studies in SI.

"...most hair proteomics studies have identified only a few hundred proteins[18,19]. A recent large-scale study[20] (130 samples) identified ~5000 proteins in all samples, likely reflecting both sample heterogeneity and improved detection methods (Extended Data Figure 5B). Proteins uniquely identified by this study were enriched in platelet- and fibrinogen-related pathways, suggesting possible blood contamination (Extended Data Figure 5C, 5D) ..."

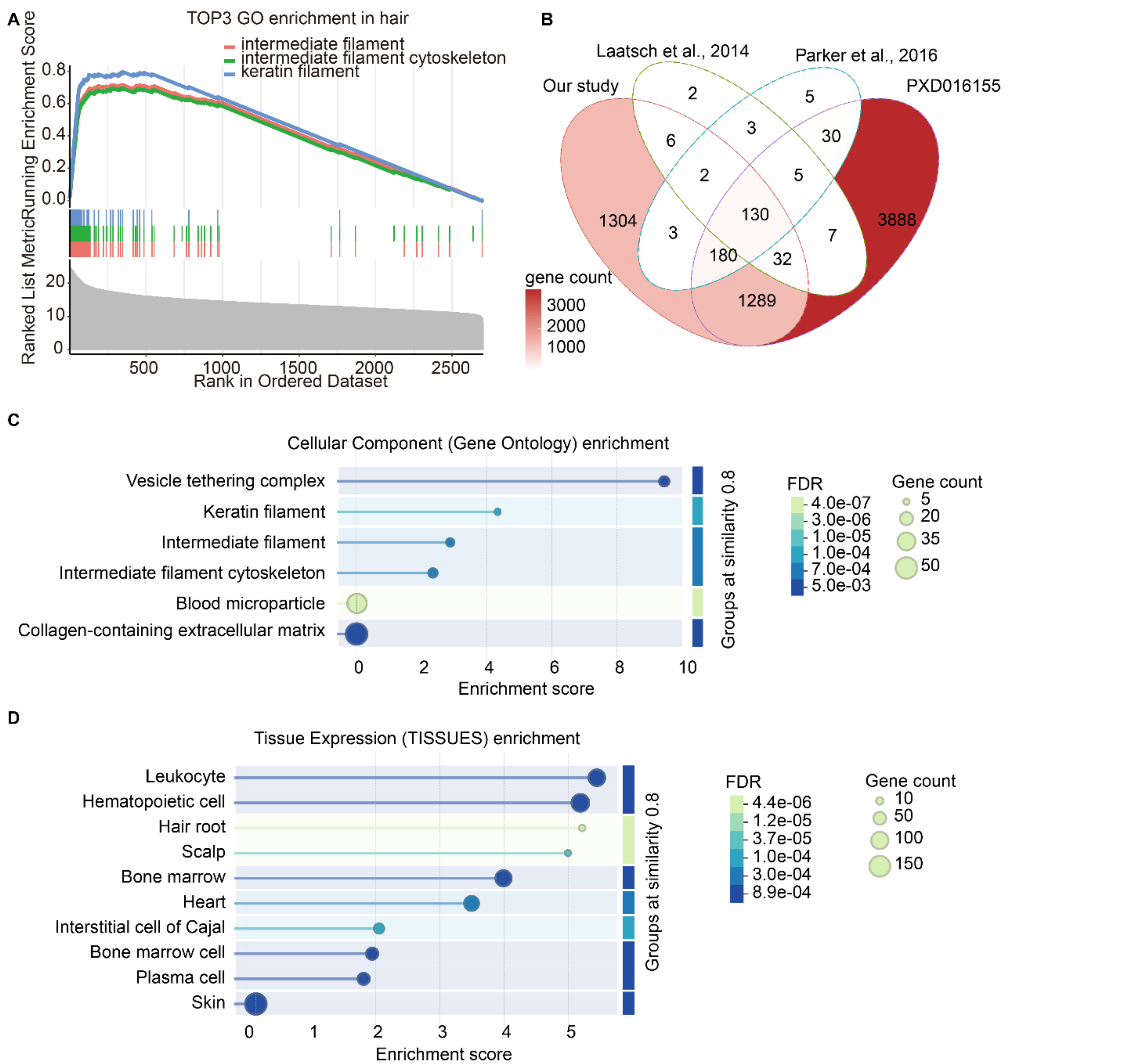

Extended data figure 5. Hair proteome composition. A, Gene set enrichment analysis of the average intensity of hair proteome, highlighting the top three enriched Gene Ontology Cellular Component terms. **B,** Venn diagram showing the overlap of identified hair proteins between our study and published datasets. Numbers indicate protein counts for each subset. **C-D**, Over-representation analysis of proteins

uniquely identified in our study using Gene Ontology Cellular Component (**C**) and Tissue Expression (TISSUES) database (**D**). Dot size scales with gene count, and color represents FDR.

"...Analysis of blank controls indicated residual high-abundance blood and intracellular proteins in the LC-MS system, as hemoglobin, actin, and GAPDH were consistently detected (Extended Data Figure 5A). These proteins can be regarded as background contaminants, and we are expanding a contamination library to further mitigate their impact on pre-test tissue-specific analyses (Table S1). Contaminant proteins were predominantly detected in body fluid, hair, and nail samples (Extended Data Figure 5B). To ensure robust tissue-specific profiling, these tissue types were excluded from downstream tissue specificity analyses. Additionally, contaminant proteins identified in other tissue types will be filtered out using the updated contamination library..."

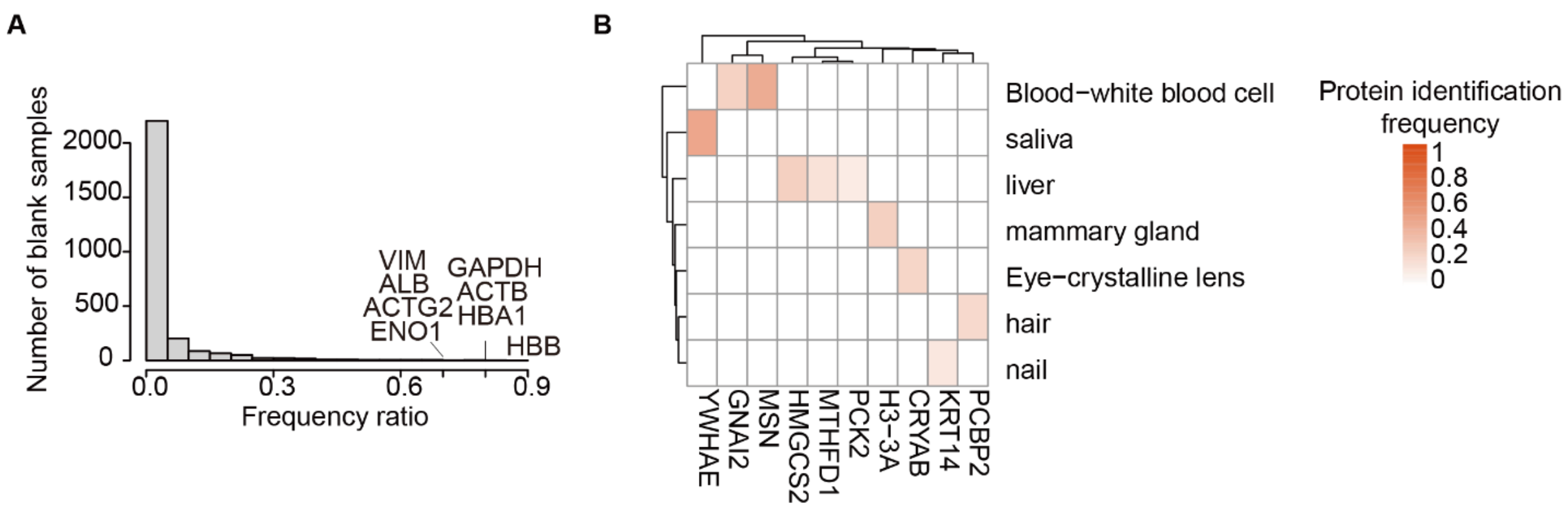

Extended data figure 5. Background contaminants detected in blank samples. **A**, Histogram of protein detection frequency in LC–MS/MS blank controls, proteins repeatedly detected across blanks. **B**, Heatmap showing tissue-enriched representative contaminant proteins across sample types. Color indicates the percentage frequency of detection in blanks for each protein displayed.

2. Furthermore, the rationale for the MS data acquisition strategy should be explained in more detail, i.e. i) why MS1 quantitative information was not already used from the DDA dataset and ii) what is the study-specific benefit of constructing the massive spectral library, while common DIA processing engines (including DIA-NN) are capable to perform comprehensive library-free searches.

Reply: We thank the reviewer for bringing these questions to our attention.

(i) The DDA data were generated solely to construct the spectral library. We used pooled samples prepared by combining equal aliquots from the same tissue type (Figure S2C), followed by high-pH reversed-phase fractionation into multiple fractions. These pooled and fractionated samples do not reflect biologically meaningful quantitative relationships and are thus inherently unsuitable for MS1-based quantification.

(ii) While recent benchmark studies[21-23] have demonstrated that library-free workflows can perform comparably or even favorably in certain contexts, our empirical results from a 48-run subset show that library-based DIA-NN analysis yielded substantially higher numbers of precursor (259,625 vs. 200,443) and protein identifications (12,256 vs. 11,201). This is likely due to the fact that we generated a comprehensive spectral library with the samples to be analyzed with DIA-MS.

More details of the results of two workflows are provided in the Table R2 only for reviewer.

3. In this regard, the common thread regarding sample preparation, replication and data standardization remains elusive. As the authors state in the last paragraph of the main text, different sample preparation methods were used for subsets of tissues, inevitably raising the questions for batch effects and quantitative integrity. To account for that, in FigureS2F the authors nicely show that DIA samples cluster based on their pathophysiological profile. However, compared to FigureS2E this pattern strongly correlates with another variable, which could be either the month of acquisition (actual figure) or the batch of "glass capillary" that was used (figure legend). These inconsistencies should be corrected and discussed in their intended context to enable proper assessment by the reader.

Reply: We thank the reviewer for pointing out this issue. In the legend of Figure S2E, "Glass capillary", should be "Year-month". This is a typo. We are sorry for this typo and appreciate the reviewer for pointing this out.

In revised results in SI, we quantitatively evaluated batch effects using Principal Variance Component Analysis (PVCA), which quantifies variance introduced by biological (e.g., sample/tissue type) and technical factors (e.g., instrument, data acquisition batch, sample collection batch).

"...we performed batch correction and evaluated the batch effect by Principal Variance Component Analysis (PVCA). As shown in Figure S2E, biological factors account for ~66% of total variance, with tissue type being the largest contributor (50.94%). Technical factors collectively explain ~4% in all, with batch plus major tissue type contributing 1.49% and instrument plus major tissue type contributing 1.33%. Other technical variables were below 1% each. This demonstrates that the observed proteomic structure is primarily driven by biology rather than technical noise..."

The improved visualization provides a statistically rigorous foundation for downstream interpretation and confirms the robustness of our experimental and analytical workflow.

2, All samples were processed using a standardized protein extraction protocol, with the exception of certain tissue types—such as hair, nails, and body fluids—where inherent biochemical properties necessitated protocol optimization for effective protein isolation[24].

As shown in the t-SNE plot (Figure S4G), these tissues form distinct clusters separate from conventional tissue types. Given their substantial biological divergence from other tissues and their minimal contribution to the systemic drug target distribution landscape, we excluded hair, nails, and body fluids from the tissue specificity analysis. This ensures greater accuracy and relevance in interpreting tissue-restricted target expression patterns across canonical human tissues.

Revised text in SI:

"...The tissue specificity of quantified proteins of normal tissue samples with only autopsy specimens, excluding body fluids, hair, nail, umbilical cord, and aborted fetus, was estimated using the modified Human Proteome Atlas (HPA) classification method[5]...After filtering, we retained 10,169 proteins and 466 normal adult samples across 64 major tissue types..."

In summary, while we acknowledge the theoretical concern about batch effects due to varied sample preparation methods, our comprehensive evaluation demonstrates that these effects are minimal and do not compromise the quantitative integrity of our dataset. The biological signals clearly dominate over technical variation, and our approach of including batch factors as covariates when necessary provides additional robustness to our findings.

4. The measures taken throughout the study for sample replication and randomization should be clarified, as well as the inclusion of a "sample pool", which is mentioned, but not explained regarding composition, sampling strategy and data processing.

Reply: Thank you for this insightful comment. We have added these technical details into the "Quality control and preprocess" session in Materials and Methods part in revised SI, as copied below.

"...We employed both biological replicates (independent processing of aliquots from the same tissue sample) and technical replicates (repeated injections of identical peptide samples), with random assignment to minimize bias. Samples were randomized by reshuffling each collection batch, and clinical metadata were blinded during processing. Quality control utilized a dual approach: a project-specific pool prepared by mixing equal amounts of peptides from early-collected samples, and a universal standard (mouse liver digest) monitored by specialized MS administrators. Both quality control samples were injected before each batch to monitor instrument stability. All samples, including project-specific pool samples, were analyzed by DIA-NN and pre-processed together (3005 raw files in total) ..."

5. Besides these open methodological questions, in Figure 2 the authors nicely show the somehow expected observation that different tissue states can be tracked based on their proteome profile. To tease out differences they focus on the distinct behaviour of brain samples; However, such a sub-analysis may also be interesting for other tissue types. This would strengthen the uniqueness of the "brain profile" and otherwise help to rule out batch effects, e.g. based on sequential analysis of samples from the same specific tissue during a given period of time.

Reply: We thank the reviewer for this insightful suggestion. In response, we have incorporated a new data analysis section in the Results.

In Results section of main text:

"...Interestingly, brain and liver tumors and their paired non-tumor tissues deviated from this F-T-NT-N pattern, clustering together rather than with their respective sample types (Figure 2B). Next, we applied trajectory analysis to quantify these observations by assigning a pseudotime value to each sample based on its relative position along the developmental trajectory from fetal samples, thereby highlighting tissue-specific F-T-NT-N transitions (SI). Brain tissues exhibited exceptional proteomic stability during malignant transformation and development, characterized by low pseudotime values and minimal variance across all four states (Extended Data Figure 8, SI), consistent with functionally constrained gene expression during brain development[23]. Conversely, liver tumor and non-tumor samples clustered at high pseudotime values, distant from fetal liver, potentially attributable to the adaptive plasticity of liver in response to variable environmental factors[23].

To elucidate the specific proteins and biological processes underpinning this trend, we conducted unsupervised clustering analysis on all the samples, which identified eight distinct protein modules characterized by coherent expression patterns (Figure 2C). Module 6, notably, showed a descending F-T-NT-N trend and was highly enriched for RNA splicing, reflecting its critical role in both organ development and oncogenesis[24]. In contrast, Module 1 exhibited a progressive upregulation along the F-T-NT-N axes and was significantly enriched in humoral immune response (Figure 2C), likely reflecting suppressed or incomplete humoral immunity in prenatal and tumor samples[25,26]. Unsupervised clustering analysis of liver and brain samples separately showed that while decreased RNA splicing and increased immune activation were also observed during the F-T-NT-N trend, tissue-specific functions were also enriched, like enhanced synaptic transmission pathways in brain and metabolic activities in liver (Extended Data Figure 9, SI) ..."

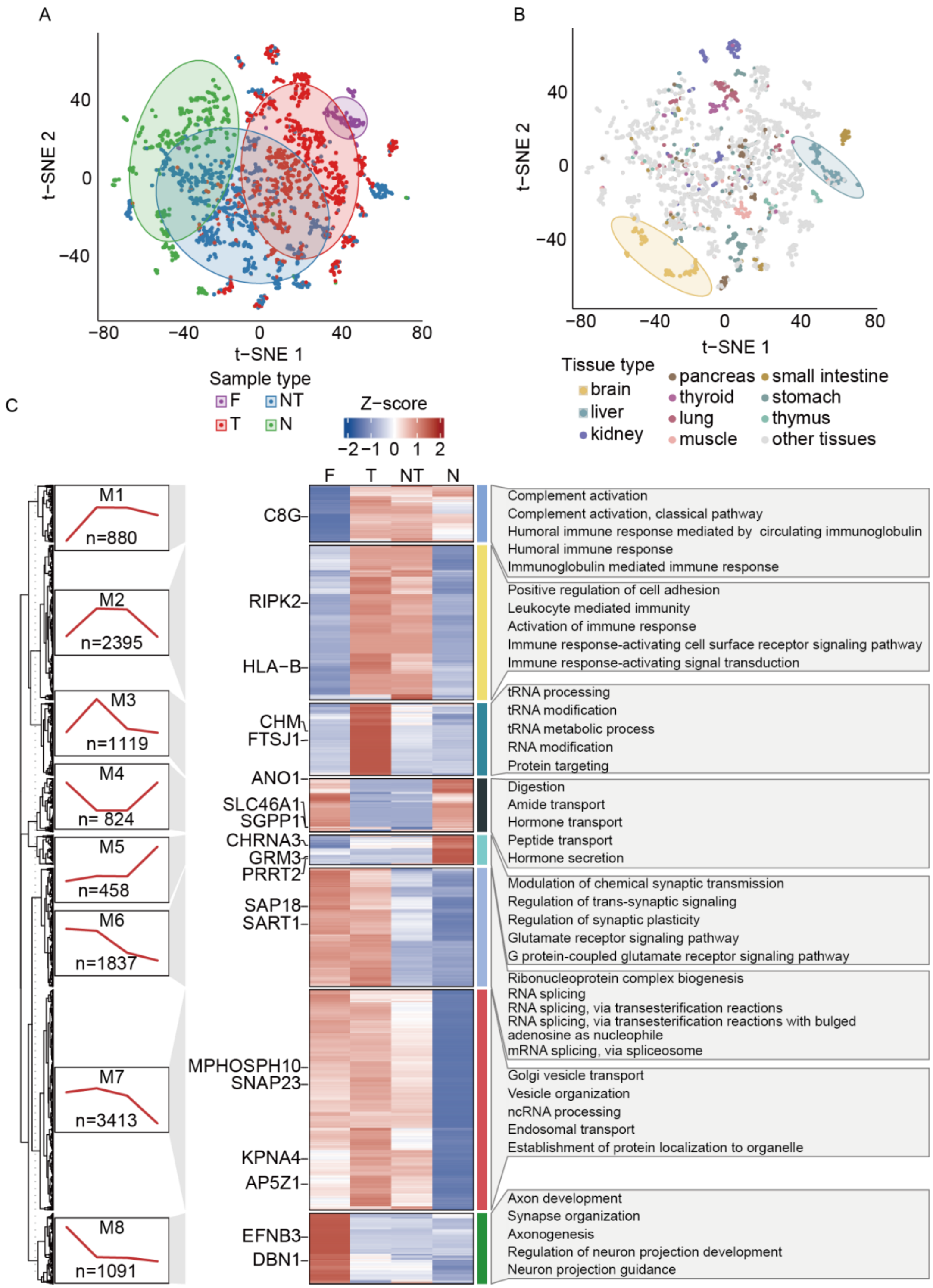

Figure 2. Proteome transformation in tissue development and oncogenesis. A & **B**, t-SNE plots of tumor (T), paired non-tumor (NT), normal adult (N) and fetal (N) samples with sample type (**A**) and tissue type classification (**B**) highlighted. **C**, Heatmap of trajectory-associated proteins (rows) clustered into eight co-expression modules based on expression in F, T, NT, N. Each module is annotated with its top five enriched Gene Ontology Biological Process (GOBP) terms ($q < 0.05$, ranked by $-\log_{10}(q)$). Z-scores reflect relative expression per protein.

In Supplementary results section of SI:

"...To characterize the dynamic landscape of proteomic remodeling across developmental and pathological states, we performed trajectory inference on fetal (F), tumor (T), paired non-tumor (NT), and normal (N) tissues using UMAP and pseudotime modeling. Analysis of 2,847 human samples exhibited a continuous, ordered distribution of pseudotime—spanning F, N, NT, to T states—mirroring development and tumorigenesis (Extended Data Figure 8). This global trajectory was accompanied by distinct tissue-specific topologies: brain samples clustered closely, indicative of minimal proteomic divergence, whereas other tissues formed discrete subclusters (Extended Data Figure 8). Notably, brain tissues exhibited exceptional proteomic stability during malignant transformation and stayed at a low level of pseudotime, with low variance across F-N–NT–T axes (Extended Data Figure 8). These results highlight the unique resilience of the brain proteome and delineate tissue-specific trajectories of proteomic reorganization in tumorigenesis..."

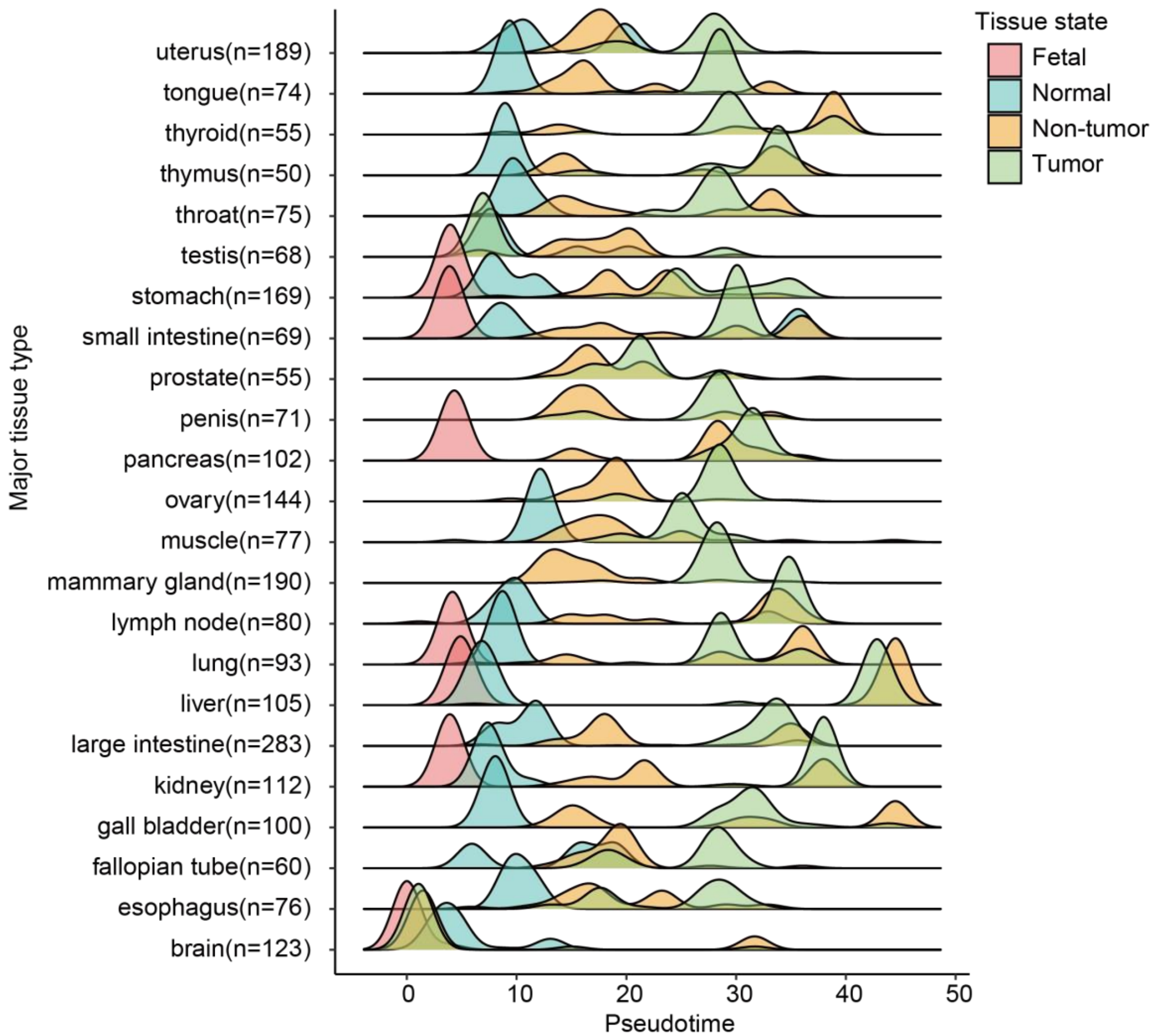

Extended data figure 8. Ridge plots showing pseudotime distributions across 23 tissue types with multiple tissue states, stratified by sample type (color-coded).

"...Unsupervised clustering analysis by ClusterGVis[19] exhibited both conserved and organ-specific molecular changes across the F–T–NT–N transition. Across all tissues, this transition consistently involved the progressive downregulation of RNA splicing and ribosome biogenesis, along with increased

immune activation and metabolic reprogramming. However, substantial regulatory differences were also observed. In the liver, Ras signaling pathways peaked in clusters corresponding to T states. The brain, on the other hand, showed enrichment of adaptive immune-related pathways in similar clusters. Both organs exhibited increasing enrichment in metabolism as the trajectory progressed (F–T–NT–N). Notably, the brain exhibited a marked upregulation of synaptic transmission-related pathways along the trajectory, reflecting ongoing neuronal maturation and circuit refinement in postnatal development[20,21], processes typically suppressed in tumorigenesis[22]. This sustained activation suggests a tissue-intrinsic differentiation program that remains partially preserved even in the transformed state…"

6. Beyond general sample classification the authors touch on their approach to exploit the dataset to inform diagnosis and therapy of various cancers. This has high potential for innovation; however, the main limitation is the low number of individual patients for each of the analyzed cancer entities. A growing body of evidence suggests that inter- and even intra-individual heterogeneity is critical to inform such studies for precision medicine apart from the obtained pan-cancer motifs. This is not addressed in the study. Hence, the clinical value for both diagnostics and cancer therapy is limited.

Reply: We thank the reviewer for this thoughtful comment and appreciate the opportunity to clarify our study design rationale and its clinical implications.

1, As discussed in the Conclusions, our current objectives differ from those of large-scale cancer cohort studies.

Conclusions:

"Here, we present an anatomically resolved human proteome atlas spanning four pathophysiological states including fetal, normal adult, tumor, and paired non-tumor tissues, providing a unified resource to delineate tissue-specific proteomic distribution and prioritize therapeutic targets...."

2, In response to this concern, we have substantially expanded our cohort, increasing the pan-cancer sample size from 519 to 1006 patients, improving the statistical power for pan-cancer analysis (see Supplementary Table 1). While our study design prioritizes tissue-type breadth over per-cancer depth, our unified experimental platform and standardized analytical pipeline provide distinct advantages that complement large single-cancer cohorts like CPTAC. Specifically, 1) We conduct rigorous cancer-specific signature identification with minimal confounding batch effects that would otherwise necessitate removal of genuine inter-cancer biological differences in multi-project meta-analyses ; (2) We conduct multiple-way comparisons (F-T-NT-N) rather than being limited to comparisons between tumor and non-tumor or molecular subtyping analyses (Figure2, 5, 6); (3) Crucially, the significantly higher intensity of tumor-enriched proteins, compared to both paired non-tumor tissue and all normal samples, suggests a strong therapeutic targeting potential with minimal predicted off-target side effects, supported by our comprehensive baseline comparisons (Figure 6). These findings highlight the critical importance of these cancer-specific proteomic signatures for both elucidating disease mechanisms and developing effective, targeted therapeutic strategies.

3, While we acknowledge that individual patient heterogeneity cannot be fully captured with our current sample sizes per cancer type, our approach identifies robust pan-cancer therapeutic vulnerabilities and tissue-specific drug targets that have been validated through orthogonal analyses. For example, we integrated our findings with CPTAC/TCGA datasets and cancer cell line databases[4] to confirm target druggability and expression patterns, demonstrating reproducibility beyond our study (Figure 5D, S5A-F).

4, We have adopted a statistical method more suitable for comparative analysis with small sample size, also considering the clinical factors.

In revised SI:

"...Given the limited sample size for each cancer type, inherent tumor heterogeneity, and potential confounding from technical batch effects, we applied a linear mixed model for the comparative analysis between paired T and NT samples for each cancer type. Batch effects and clinical factors (sex, age, subtype) were explicitly included as covariates in the differential expression analyses to ensure robustness, with standardized effects calculated following Hedges' small-sample correction[40]. This dual strategy enhances the reliability of biological signal detection while minimizing false positives that may arise from technical variation[41-43]..."

7. Some proteins and protein signatures are put in biological/clinical context by the authors. However, none is experimentally validated leaving the present study purely descriptive.

Reply: Thank you for the insightful concern. Given the systemic and large-scale nature of our study—spanning dozens of tissue types and tissue states—comprehensive experimental validation of all findings is inherently challenging, and mechanistic studies of individual proteins are beyond the scope. However, we have conducted extensive targeted and cross-study validation.

1)We performed large-scale PRM assays across over 1,000 samples, validating tumor-specific proteins and tumor-enriched proteins in both normal tissues and 25 matched tumor-adjacent pairs (Figure 6, Extended Data Figure 12). This represents one of the most comprehensive targeted proteomic validations in a single study.

Relevant excerpts from revised Results section in main text.

"…Targeted MS-based quantification validated the cancer-specific upregulation of CPT1C and FXYD6 in GIST…"

"…Targeted proteomic analysis confirmed PAX5 as tumor-specific and tumor-enriched in DLBCL (Figure 6C, 6F) ..."

In revised SI:

"...To validate cancer-specific protein dysregulations, we performed parallel reaction monitoring (PRM) targeting 20 candidate proteins across a subset of paired tumor and non-tumor samples collected before year 2022, along with most normal tissue samples. Among these, six showed consistent dysregulation patterns with DIA-based quantification (Figure 6B–C, Extended Data Figure 12). ADAMDEC1 (ADAM-like decysin-1) was found to be specifically upregulated in DLBCL both in our DIA and PRM data (Extended Data Figure 12). Consistently, Zhu et al. found that ADAMDEC1 was upregulated in DLBCL, and its expression levels correlated with tumor progression and poor patient survival[29]. EZH2, a critical "gatekeeper" in the lymph node that allows B cells to mature[30], was also validated as a cancer-specific DEP for DLBCL (Extended Data Figure 12). In DLBCL, this gatekeeper is mutated (Y641) or overexpressed, permanently locking cells in a growth phase and hiding them from the immune system... "

2) We also cross-validated our pan-cancer dysregulations with CPTAC and TCGA (Figure S6A-B).

In revised Results:

"…We cross-validated the DEPs with TCGA transcriptomic and CPTAC proteomic datasets (Figure S6A, S6B). Despite differences in platforms and cohorts, we identified 4263 upregulated and 1716 downregulated DEPs consistent across our data, CPTAC and TCGA. We further integrated our DEP list with prognostic data from the Human Pathology Atlas[42], identifying 7336 proteins associated with clinical outcomes across 16 cancer types (Figure S6C, Table S6). Notably, PLOD2 was upregulated in 13 of 25 tumors (Table S6). Previous pan-cancer study has also reported common upregulation of PLOD2 in

T compared to adjacent NT, and associated the upregulation with poor prognosis in renal carcinoma (RC), lung adenocarcinoma, and pancreatic ductal adenocarcinoma[43], representing a high-confidence candidate for biomarker development and therapeutic targeting…"

3) We integrated cell line drug sensitivity profiles, and CRISPR-Cas9 functional genomics data (Figure 5).

"…we integrated our dataset with drug sensitivity and CRISPR gene essentiality data to prioritize candidate protein targets and drugs. Specifically, we mapped upregulated DEPs to the ProCan-DepMapSanger proteomic dataset, which links protein abundance with both IC50 values (drug sensitivity) and CRISPR-Cas9 gene essentiality in pan-cancer cell lines. This integration identified a total of 35 drugs targeting nine DEPs, predominantly RTKs (Figure 5B, Table S8). Given the frequent occurrence of RTK alterations at both the genetic and protein level (Figure 5A, S5D), our focus narrowed to RTK proteins. We prioritized those RTKs that were simultaneously: 1) significantly upregulated in tumors (Hedges' $g \geq 0.5$, B-H adjusted $p < 0.05$) and 2) predictive of a higher drug potency at higher protein intensity ($\beta < -0.1$, FDR < 0.01, Figure 5B). We observed that high MET and BCL2L1 expression correlated with increased potency of Savolitinib/Tepotinib (MET inhibitors) and Navitoclax (Bcl-2 family inhibitor) in colorectal carcinoma cell lines. In RECA, the therapeutic viability of targeting MET was further supported by the correlation between MET protein levels and CRISPR gene essentiality, where higher abundance correlated with greater dependency ($\beta < -0.1$, FDR < 0.01, Figure 5C) …."

5) Like previous pan-cancer and human proteome studies[7,11,25-28], rather than focusing on experimental validation of a limited set of arbitrarily selected candidates to uncover underlying mechanisms—a process inherently constrained by scale and context—we have designed this resource as a publicly accessible knowledge base (available at https://db.prottalks.com/) to serve the broader research community. This platform enables researchers to explore, apply, and validate our findings across diverse biological systems and clinical settings according to their specific interests and experimental models.

8. The authors should analyze interpatient variability within the individual cancer entitites and should investigate whether they can identify patterns of biological/ clinical relevance beyond known ones.

Reply: We thank the reviewer for this suggestion and have performed new data analysis to address these issues. Acknowledging sample size limitations (average 20 patients/entity), we increased the pan-cancer sample size (average 40 patients/entity). While identifying novel within-entity patterns was beyond our study scope, we discovered previously uncharacterized pan-cancer molecular subtypes through pan-cancer unsupervised analysis (Extended Data Figure 11).

In revised SI:
"...Pan-cancer proteome consensus clustering revealed five distinct tumor subtypes (Extended Data Figure 11). Cluster 1 was enriched in squamous carcinomas (CESC, LARCA, PECA, TOCA, ESCA) and was characterized by elevated interferon response, TNF-α, and p53 signaling. Cluster 2 primarily comprised pancreatic adenocarcinoma (PACA) and gallbladder carcinoma (GBCA), characterized by active protein secretion. Cluster 3, dominated by hepatocellular carcinoma (HCC), renal carcinoma (RC), glioblastoma (GBM), and gastrointestinal stromal tumor (GIST), represented the metabolism-activated subtype and was enriched for metabolic pathways. Cluster 4 primarily contained testicular germ cell tumors (TGCT) and diffuse large B-cell lymphoma (DLBCL), characterized by enhanced proliferative signatures. Cluster 5 primarily included muscle tumor (MUT) and prostate carcinoma (PRCA), characterized by active myogenesis and upregulation of UV response pathways."

Bioinformatics:

The authors employ a state-of-the-art computational pipeline for facilitating a proteomic mapping across many tissue types and cancer samples. However, I have some concerns and several aspects of the bioinformatics workflow—ranging from data pre-processing to downstream statistical analysis — should be more rigorously explained and clearly justified.

1. My main concern regards the inconsistent and sometimes arbitrary choice of parameters in different parts of the pipeline. Please justify choice and use consistent values throughout the analysis in the following aspects: Different imputation methods are used in distinct parts of the analysis. For example, the authors mention filling missing values with 0.5 times the minimal value in the whole matrix in the tissue specific context while also using random numbers drawn from a normal distribution scaled by a minimal value in the matrix in the pan-cancer analysis. How robust are the downstream analyses when instead using state-of-the-art imputation algorithms such as the dreamAI algorithm? (MA, Weiping, et al. DreamAI: algorithm for the imputation of proteomics data. Biorxiv, 2020, S. 2020.07. 21.214205.)

Reply: We sincerely appreciate this technical suggestion. The differential imputation strategies between tissue-specific (0.5×min matrix value) and pan-cancer analyses (normal distribution scaled by 0.5×min value) were intentionally designed to accommodate distinct statistical requirements. In pan-cancer studies where linear models are employed, the introduction of normally distributed noise ($\mu=0$, $\sigma=0.5\times\text{min}$) prevents artificial inflation of significance from identical imputed values, while maintaining biologically plausible value ranges. This technical nuance is less critical in tissue-specific analyses where significance testing is not performed.

Per the reviewer's recommendation, we have conducted supplementary comparative analyses using the state-of-the-art dreamAI[29] algorithm. However, due to the high dimensionality and sparsity of our proteomic matrix—where over 50% of protein intensity values are missing across all samples—the algorithm encountered significant computational challenges. Despite using high-performance computing resources, the execution required several weeks and ultimately terminated with unresolved computational errors. We made multiple attempts to troubleshoot the issue, including the imputation submatrix, but the program failed to complete for all the submatrix. Furthermore, we reached out to the development team via the official GitHub repository for technical guidance, but have not yet received a response (https://github.com/WangLab-MSSM/DreamAI/issues/15). While the integration with DreamAI was not successful, we note that our dataset demonstrates robust technical quality, as evidenced by high reproducibility in replicate measurements (Figure S2C, S2D) and minimal batch effects (Figure S2E)—despite the use of simple imputation strategies.

2. Similarly, inconsistent thresholds for fold change were chosen, with 1.5 for the paired tumor/normal analysis and 2 for the drug analysis.

Reply: We thank the reviewer for highlighting this methodological consideration. Our selection of fold-change thresholds was strategically tailored to the specific goals of each analysis. For the initial pan-cancer Tumor/Normal analysis, we employed a 1.5-fold change threshold, aligning with TCGA pan-cancer RNA-seq thresholds[30]. A higher 2.0-fold change threshold was utilized for the subsequent drug analysis to prioritize and identify high-confidence therapeutic targets. In this version, we applied effect size analysis and a uniform threshold of B-H adjusted p value less than 0.05, and adjusted |Hedges's g| more than 0.5.

In Materials and Methods section of the revised SI:

"...The p-values were adjusted for multiple testing within each cancer type using the Benjamini–Hochberg procedure, and proteins were considered significantly differentially expressed proteins (DEPs) if they satisfied adjusted $p < 0.05$ and $|\text{Hedges' } g| \geq 0.5$..."

3. The choice of perplexity for t-SNE as (n-1)/3-1 seems unconventional - could you motivate this? How robust was the analysis wrt to this choice?

Reply: Thank you for this comment on technical details. We selected the perplexity value of (n-1)/3-1, where n represents the number of samples used for clustering, based on established computational constraints and visualization objectives. Specifically, t-SNE requires that perplexity × 3 < n-1 to ensure mathematical validity[31]. Our formula approaches this upper bound to maximize the number of nearest neighbors considered, thereby promoting more global structure preservation while maintaining local relationships. This higher perplexity value facilitates better clustering coherence by allowing each point to consider more neighbors during the optimization process, which is particularly important for identifying biologically meaningful cell populations in our high-dimensional dataset.

To address the reviewer's concern regarding robustness, we re-evaluated the analysis using an alternative perplexity value. The F-T-NT-N trend remains consistent, supporting the stability of our findings (Figure 2A).

Revised text in SI:

"...The perplexity parameter was set to 10."

4. For the hierarchical clustering for pancancer analysis I wonder how robust the clustering is – in proteomics analyses, correlation-based metrics can sometimes capture relationships more robustly. In addition, does the structure hold when performing a consensus clustering?

Reply: We appreciate the reviewer's insightful comment. While hierarchical clustering was only performed on normal samples in the previous version, we have now performed consensus clustering across all cancer samples in this revision to evaluate the stability and robustness of the clustering structure (Extended Data Figure 11).

5. For the batch-effect correction, more quantitative evaluations of the batch-effect removal (e.g., before–after metrics/plots, clustering statistics) would be helpful; also, in the downstream analysis, were batch variables included in limma?

Reply: Thank you for this considerate comment. We have evaluated the batch effect by Principal Variance Component Analysis (PVCA), as detailed in the revised SI.

"...As shown in Figure S2E, biological factors account for ~66% of total variance, with tissue type being the largest contributor (50.94%). Technical factors collectively explain ~4% in all, with batch plus major tissue type contributing 1.49% and instrument plus major tissue type contributing 1.33%. Other technical variables were below 1% each. This demonstrates that the observed proteomic structure is primarily driven by biology rather than technical noise..."

The reviewer raises an important point about covariate inclusion. We used uncorrected data to avoid overcorrection and batch variables are systematically included as covariates in all linear mixed models for differential expression analysis between tumor and non-tumor samples. Model specifications are detailed in revised Methods in SI.

"...we performed differential expression analysis for each protein by fitting a linear mixed model using the lmerTest57 package, in which the log-scaled intensity was the outcome. The model included sample type (tumor vs. non-tumor) as the primary fixed effect and a patient-specific random intercept to account

for the paired-sample structure, while additional fixed covariates (cancer subtype, sex, age, and dataset) ..."

6. For the integration with external data (mapping to drug targets), could you clarify how mismatches in protein identifiers, isoforms, or quantification differences were handled?

Reply: Thank you for this detailed and thoughtful question. We added more technical details in the session titled "Comparison with previous human transcriptome and proteome draft" in SI.

"...For comparison with previous human proteome drafts, we employed Ensembl stable gene IDs (version GRCh37.p13) as our primary mapping strategy, ensuring consistency across data versions and minimizing gene symbol ambiguity. We exclusively utilized canonical reviewed proteins from UniProt, mapped to stable gene IDs via BioMart, thereby eliminating isoform mismatch issues. To address quantification differences, we implemented two complementary strategies: (i) z-score normalization of overlapped proteins prior to Pearson correlation analysis for cross-study expression comparisons, and (ii) comparison of tissue specificity labels derived from independent multi-tissue proteome analyses within each study, following established methodology[11].

For DrugBank integration, we directly utilized UniProt IDs documented in DrugBank for drug target mapping, maintaining database-native identifiers to minimize conversion errors. These methodological details have been incorporated into the revised Methods section in Supplementary Information.

"...The list of cancer dysregulated proteins was mapped to the DrugBank online database[32] to find out potential targeted drugs by UniProt IDs documented in DrugBank..."

Finally, for reproducibility, the entire computational workflow should be made accessible through open code and well-annotated analysis pipelines.

Reply: We thank the reviewer for this important suggestion regarding computational reproducibility. We have addressed this concern by organizing and documenting all computational scripts used in this study. The complete analysis pipeline, including data preprocessing, statistical analyses, and figure generation scripts, is now publicly available on GitHub (https://github.com/Wenhhao/TPHP).

Minor comments:

• The abbreviations introduced in Figure1 are difficult to track in the following figures. It may be beneficial to use the full descriptions instead.

Reply: We have replaced all abbreviations for tissue in the main figures with their full descriptions to improve clarity and consistency.

• In Figure 2B the color-coded tissue class does not appear in the actual graph.

Reply: We apologize for the confusion. In the previous version, the legend was grouped centrally, which made it difficult to associate color codes with corresponding tissue classes in Figure 2B. This has now been corrected in revised Figure 2.

• The section about brain development requires references (line 149 and following).

Reply: Thank you for the comment. In the previous version, the brain development analysis was based on Aguet et al.[32]. However, in the current revision, we have removed specific developmental claims.

• The tissue classification metrics should be explained more in detail (line 169 and following).

Reply: we have refined the details about the metrics in SI.

"...The tissue specificity of quantified proteins of normal tissue samples with only autopsy specimens, excluding body fluids, hair, nail, umbilical cord, and aborted fetus, was estimated using the modified Human Proteome Atlas (HPA) classification method[54]. The protein abundances of each tissue type were aggregated by median of all samples after imputing missing values to 0.5 times the minimal value in the whole matrix. After filtering, we retained 10,169 proteins and 466 normal adult samples across 64 major tissue types...."

More details about the tissue specificity metrics could be found in Table S2.

• In FigureS1 the legend does not fit to the actual illustrations.

Reply: we have refined the legend as excerpted below.

"Figure S1. A view for quality of the spectral library. A view of the quality of the spectral library. A, *Workflow of construction of DDA-based spectral library.* ***B-C,*** *Results from DIALib-QC showing the percentage of precursor m/z (**B**), precursor charge (**C**).* ***D,*** *Pearson correlation of iRT values between +2 and +3 precursors with the same peptide sequence.* ***E,*** *Percentage of peptide length.* ***F-G,*** *Number of modifications (**F**), peptides per protein (**G**).* ***H-L,*** *Percentage of fragments per precursor (**H**), fragment ion (**I**), fragment ion charge (**J**), protein coverage (**K**) and missed cleavage ratio (**L**).* ***M-N,*** *Overlapped peptides (**M**) and proteins (**N**) between our data, PeptideAtlas, and ProteomicsDB datasets."*

• In FigureS2B the color-coding does not fit, which complicates access to the illustration. Overall, the figure looks quite busy for the actual content.

Reply: We have verified the color-coding in Figure S2B is correct and have improved clarity by repositioning the legend, enhancing contrast, and reducing visual clutter.

• For FigureS4A the shown graph does not fit to the main text (line 192). Furthermore, the scope of the shown analysis requires more detailed explanation as well as suitable citations.

Reply: Thank you for the feedback. In response to the concern regarding Figure S4A, we have revised the figure from the original protein interaction network to a more intuitive tissue-enriched protein pathway enrichment dot plot, which clearly displays the top enriched biological pathways across key tissues (Figure S5A, Supplementary table, e.g., synaptic signaling in brain, metabolism in liver).

• The comparison opened in line 214 should be explained more in detail including citations.

Reply: Thank you for the feedback. In response, we have revised the text starting at line 214 to provide a more precise and biologically grounded interpretation, supported by functional evidence rather than general statements. The updated sentence now reads:

"Proteins associated with metabolism, synaptic function, meiotic cell cycle, cardiac chamber morphogenesis, and lens development were uniquely enriched in liver, brain, testis, heart, and iris, respectively...brain enrichment has been widely reported due to its distinct biological functions[8]*..."*

• In line 229 and following the correct protein name would be “BGLAP”.

Reply: Thank you for pointing out the error. We have corrected the protein name from the incorrect designation to "BGLAP" throughout the manuscript and SI.

Referee #2 (Remarks on code availability):

The entire computational workflow should be made accessible through open code and well-annotated analysis pipelines.

Reply: We thank the reviewer for this important suggestion regarding computational reproducibility. We have addressed this concern by organizing and documenting all computational scripts used in this study. The complete analysis pipeline, including data preprocessing, statistical analyses, and figure generation scripts, is now publicly available on GitHub (https://github.com/Wenhhao/TPHP).

Referee #3 (Remarks to the Author):

The manuscript, "Spatial distribution of the proteome in human body and cancers", presents a valuable and comprehensive proteomic resource, profiling 251 human tissues and 25 carcinoma types. The data have strong potential to support research in tissue specificity, cancer biomarker discovery, and drug target investigation. However, while the dataset itself is impressive in scope, the current version of the manuscript falls short in terms of data interpretation, clarity, and depth of analysis.

Major Comments

1. Data visualization and interpretability

Several figures are difficult to interpret, particularly in terms of understanding the quality and structure of the data. For instance, in Figure 2a (t-SNE), the brain grouping is emphasized, but other apparent groupings are not annotated or explored. Adding additional coloring schemes (e.g., by tissue type or anatomical group) could enhance the readability and interpretability of these dimensionality reduction plots. Similarly, Figure 3g does not clearly convey clear data patterns in its current form.

Reply: We sincerely thank the reviewer for the helpful feedback. We have made several improvements to enhance clarity and biological interpretability.

1, To improve the interpretability of the dimensionality reduction plot previously shown as t-SNE (now updated to UMAP for better global structure preservation), we have added color annotation by tissue type (Figure 2B).

2, To quantitatively assess tissue state transitions beyond visual inspection, we conducted pseudotime trajectory analysis (SI, Extended Data Figure 8). This analysis assigns a tissue differentiation score based

on the distance from the fetal state (set as the starting point). The results confirm that brain tissues follow a distinct and divergent trajectory, which reflects their unique developmental and pathological protein-level remodeling. This approach also allows for systematic comparison with other tissue types presented in the Supplementary Information (SI). Furthermore, we also observed that brain and liver exhibited a distinct trajectory. We also performed tissue-specific unsupervised clustering of protein variations across the four tissue states, which is detailed in the Extended Data Figure 8 and SI.

3, Regarding the concern about "Figure 3g", we assume this refers to Figure S3G, as no Figure 3G exists. We recognize that the original unsupervised clustering of tumor samples shown in Figure S3G did not clearly reveal distinct, interpretable patterns in the t-SNE plot. To rectify this, we have substituted this approach with a more robust and biologically informative consensus clustering framework, which is now presented in Extended Data Figure 11.

2. Limited discussion on data quality and deviations

While the authors acknowledge some heterogeneity or unexpected patterns (e.g. in tissues such as vagina, eye, cartilage, fallopian tube, and pancreas, as well as clustering involving peripheral nerve & fat, uterus & ureter, throat & salivary gland), these are not sufficiently discussed. Providing deeper insights into these deviations would increase the transparency and credibility of the dataset.

Reply: Thank you for careful consideration of this issue. We have now substantially expanded our analysis and provided detailed explanations for the observed deviations, supported by histological validation and quantitative distance metrics.

1, We have added a new section in the SI analyzing the tissue heterogeneity addressing tissue heterogeneity, and added H&E staining images for key samples showing deviations.

"...Intra-type heterogeneity was detected across all normal tissue categories. By the original major tissue type classification, all normal samples were classified into 59 major tissue types (separate blood cell and plasma as two categories). Tissue types with the highest median distance within tissue type were listed below (Table E1).

Table E1. Top 6 intra-major tissue type distance

Major tissue type	Is within tissue	Median distance	Sd distance	Min distance	Max distance
Eye	TRUE	389.02	140.20	89.95	567.30
Blood cell	TRUE	359.47	113.75	77.82	401.16
Fallopian tube	TRUE	344.18	80.20	206.11	345.87
Vagina	TRUE	333.45	NA	333.45	333.45
Pancreas	TRUE	327.63	28.05	304.97	377.02
Throat	TRUE	317.13	78.56	124.82	375.70

This high intra-heterogeneity could be due to different subtypes within the same major tissue type have distinct proteomes. For example, eye samples exhibited high internal complexity: distinct anatomical components (crystalline lens, cornea, iris, sclera) displayed markedly different proteomic profiles, and the distance between the crystalline lens and to iris and cornea is among the top 10 distances of detailed tissues within the same major tissue type (median distance: 538.9 and 395.22, Table E2), supporting their treatment as separate tissue entities for specificity analysis (Figure 3A; Supplementary Table 3). Cartilage demonstrated substantial subtype heterogeneity, particularly for epiglottis (Table E2). H&E staining showed histological features of epiglottis distinct from hyaline and fibrocartilage (Extended

Data Figure 7B-C), so we also took it as a separate tissue for specificity analysis. Sampling heterogeneity contributed additional variance. For example, thyroid cartilage from donor DL3 contained mixed tissue types, whereas DL4 was predominantly cartilaginous (Extended Data Figure 7C; Supplementary Table 3). The nasopharynx was observed to be enriched in glandular cells, while the oropharynx had fewer glandular cells but more connective tissues (Extended Data Figure 7D).

Within the vagina group, sample DIA_554 showed an anomalous proteomic profile most similar to testis based on median Euclidean distance (172.21) and Pearson correlation (0.92); because it was processed adjacent to a testis sample (DIA_555), cross-contamination is a plausible explanation. Accordingly, DIA_554 was classified into the tissue heterogeneity abnormal group (Supplementary Table 1) and excluded from the current analysis. In the fallopian tube, H&E staining indicated that epithelial thickness varied across patients, reflecting inter-individual structural differences within this tissue type (Extended Data Figure 7E). In pancreatic tissue, H&E examination showed complete autolytic necrosis in the sample from donor DL4, contrasting sharply with donor DL3, and this sample-specific degradation likely accounts for the corresponding proteomic deviation (Extended Data Figure 7F). Thus, pancreas samples from DL4 were removed before downstream analysis. Similarly, we checked other high heterogeneity categories and labeled abnormal samples or separated major categories to make the intra-tissue samples exhibit significantly higher correlation and lower proteomic distances than inter-tissue comparisons ($p < 0.001$; Figure S4A-F, Table S3) …"

Table E2. Top 10 intra-major and inter-detailed tissue type distance

**Detailed Tissue type 1**	**Detailed Tissue type 2**	**Is within detailed tissue**	**Is within major tissue**	**Median distance**	**Sd distance**	**Min distance**	**Max distance**
Crystalline lens	iris	FALSE	TRUE	538.90	18.60	514.33	567.30
Epiglottis	meniscus	FALSE	TRUE	432.14	23.05	413.17	455.77
Epiglottis	thyroid cartilage	FALSE	TRUE	420.81	82.71	324.26	502.85
Platelet	red blood cell	FALSE	TRUE	398.19	2.06	396.23	401.16
Corneal	iris	FALSE	TRUE	395.22	14.27	378.38	418.55
Costi-cartilage	epiglottis	FALSE	TRUE	391.75	24.96	356.34	416.1712
Achilles tendon	smooth muscle (pyloric region)	FALSE	TRUE	374.54	19.53	355.16	391.21
Common flexor tendon	smooth muscle (pyloric region)	FALSE	TRUE	373.14	15.855	358.91	387.40
Naso-pharynx	oropharynx	FALSE	TRUE	368.27	18.13	328.29	375.70
Red blood cell	white blood cell	FALSE	TRUE	365.16	5.44	359.47	370.29

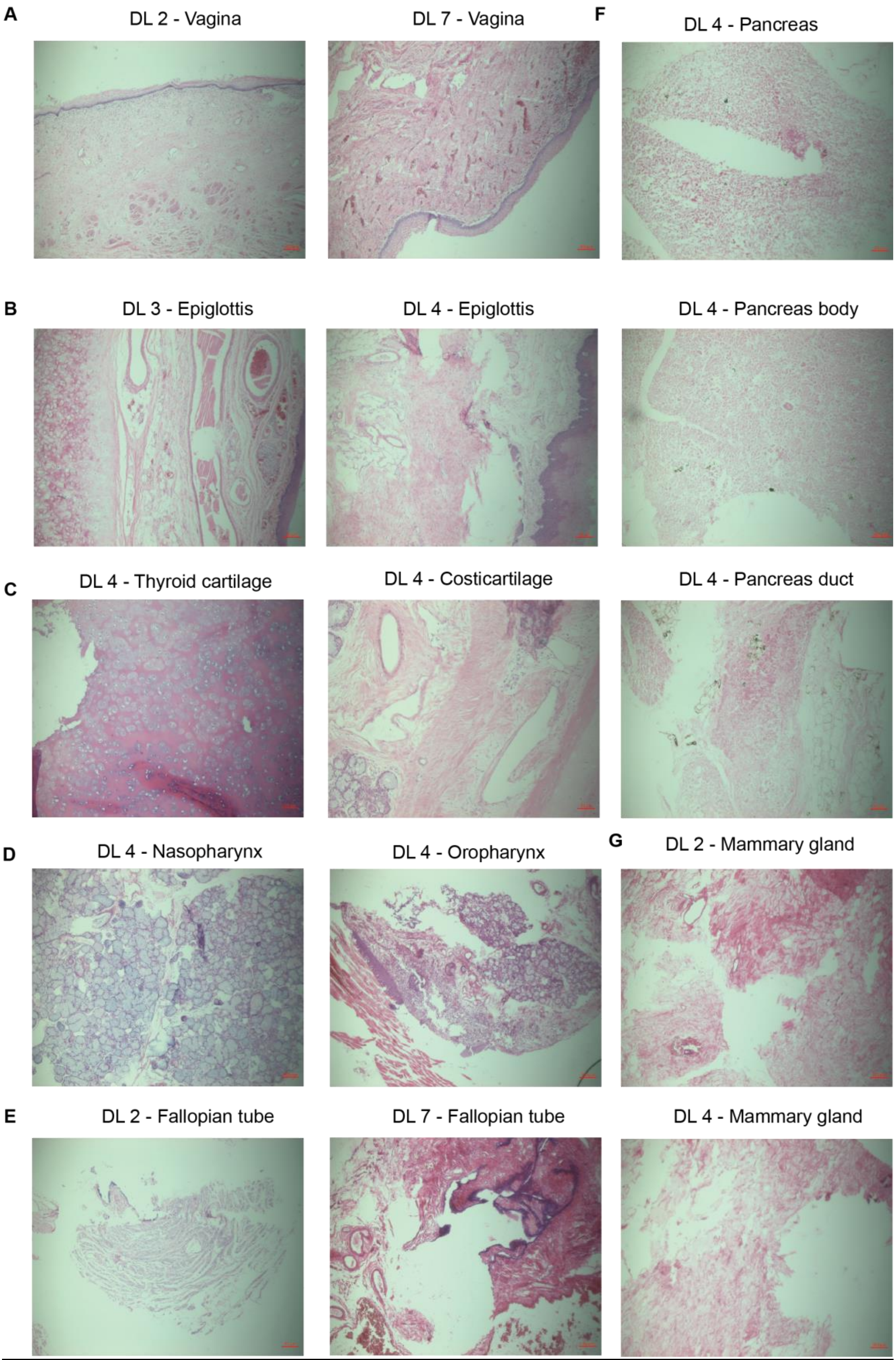
A
DL 2 - Vagina
DL 7 - Vagina
F
DL 4 - Pancreas
B
DL 3 - Epiglottis
DL 4 - Epiglottis
DL 4 - Pancreas body
C
DL 4 - Thyroid cartilage
DL 4 - Costicartilage
DL 4 - Pancreas duct
D
DL 4 - Nasopharynx
DL 4 - Oropharynx
G
DL 2 - Mammary gland
E
DL 2 - Fallopian tube
DL 7 - Fallopian tube
DL 4 - Mammary gland

Extended Data Figure 7. HE staining of tissue heterogeneity in abnormal samples. Representative Hematoxylin and Eosin (H&E) stained sections are shown at 40X magnification. (A) Vagina of DL 2 and DL 7 participants. (B) Epiglottis from DL 3 and DL 4. (C) Thyroid cartilage and costicartilage from DL 4. (D) Nasopharynx and oropharynx from DL 4. (E) Fallopian tube from DL 2 and DL 7. (F) Pancreas, pancreas duct, pancreas body from DL 4. (G) Mammary gland from DL2 and DL4.

2, For unexpected clustering, we have also checked the proteomic and histologic profiles.

1) Peripheral nerve & fat. H&E staining showed substantial adipose tissue surround in nerve samples. (Figure below, only for reviewers).

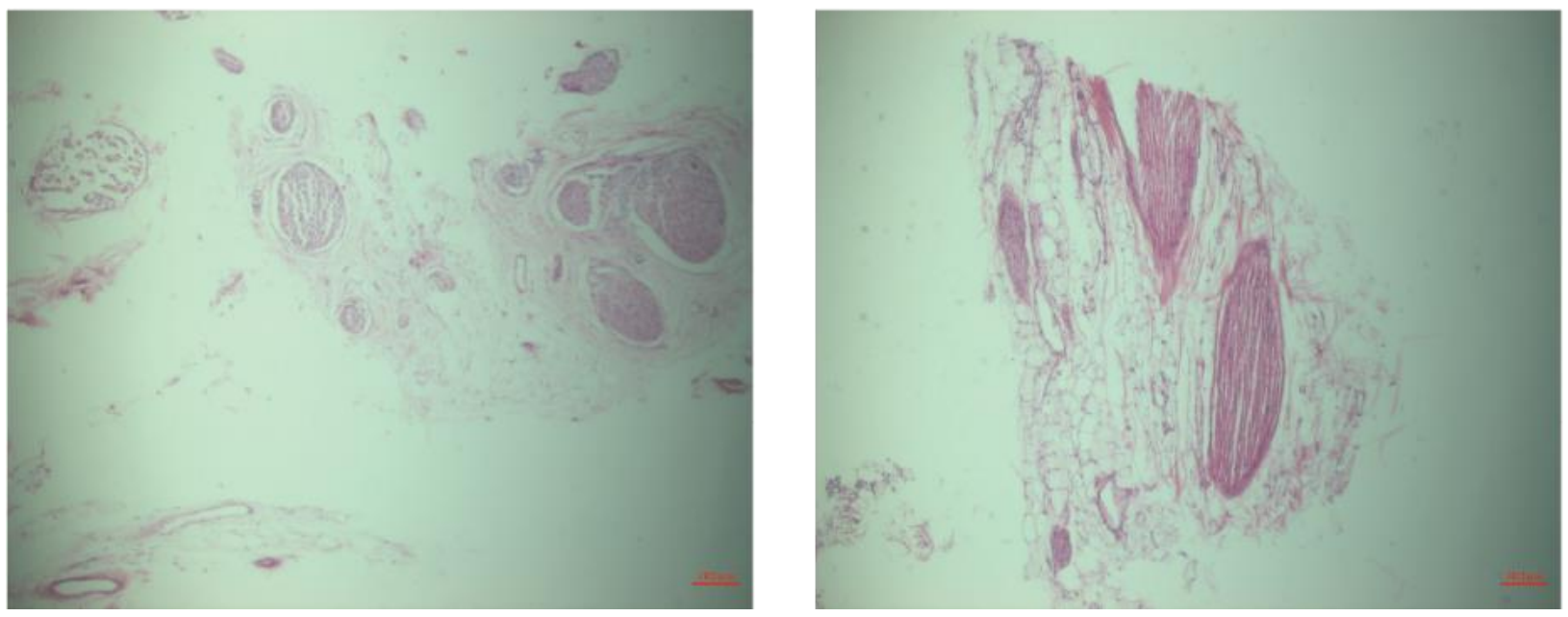

2) Uterus & ureter. This similarity is more plausibly explained by shared tissue architecture than by contamination, as uterus and ureter share core structural features as tubular/hollow organs with prominent smooth muscle layers and broadly similar mucosal architecture.

3) Throat & salivary gland. H&E staining of throat samples showed a predominance of serous/mucous glandular tissue (Extended Data Figure 7D), providing a direct explanation for the proteomic similarity to salivary glands.

In this version, we also discussed some unexpected clustering pattern in the revised Results:

"...Notable exceptions include clustering of the mammary gland with connective tissue-rich tissues, such as bone, tendon, and cartilage, potentially reflecting age-related involution, which is consistent with the paucity of mammary alveoli observed in H&E staining (Extended Data Figure 7G). Similarly, the trachea co-clustered with the salivary gland, likely due to the prominent presence of glandular epithelial cells in the sampled region of the trachea..."

3. Validation strategy

The rationale and outcome of the PRM validation (14 out of 315 cancer-specific proteins) are unclear. The authors should clarify the selection criteria for these proteins, the success rate of validation, and how these results reflect the overall reliability of the cancer-specific findings.

Reply: Thank you for noting this point. We have clarified these technical details in the SI.

In the first version, a total of fourteen proteins in the PRM assay represent the overlap between the initial 137-protein list and the final 315-protein list. Six out of 20 overlapping proteins showed concordantly significant dysregulation patterns between DIA-MS and PRM analyses, so the success rate of PRM assay development was 30%.

In the revised version, we now report 2878 cancer-specific proteins. The SI has been updated to reflect the overlap between these 2878 proteins and the PRM assay targets.

"...A total of 137 cancer-specific dysregulated proteins were selected from pan-cancer data acquired before the year 2022 for PRM validation. Each selected precursor has no modification and no missed cleavage, with the peptide length ranging from 8 to 20 using Skyline (version 21.1). In total, 57 peptide precursors from 48 proteins and 9 iRT peptide precursors were analyzed. Among the 48 cancer-specific proteins, 20 were still classified as cancer-specific.…"

We acknowledge that comprehensive validation of all 2878 proteins is beyond the scope of this study and will be addressed in a dedicated follow-up investigation focusing on targeted proteomics method development.

4. Transcriptomic vs. proteomic specificity

It would be valuable to discuss how the observed protein specificity categories compare to transcriptomic data for overlapping tissues.
Reply: We appreciate the reviewer's careful consideration of this issue. We have now expanded our analysis and discussion accordingly.

We compared proteome-based tissue specificity categories with matched transcriptome-based categories from HPA across 23 overlapping tissues. Results are summarized in revised Supplementary Table 4 and revised Results section.

"...Of the 1716 tissue-enriched proteins identified, 749 were previously reported as enriched in the corresponding tissue types in published human proteome[13,14] or transcriptome datasets[8]. Of these, 666 were supported at the protein level and 426 showed concordant enrichment in HPA RNA-seq data. Across 36 tissues overlapping with the HPA, we identified 831 proteins uniquely enriched in our dataset and 122 exclusively enriched in the HPA RNA data. Notably, 480 tissue-enriched proteins were identified in 24 tissue types underrepresented in prior studies, underscoring the expanded tissue coverage. Among these, we identified PANX3 as the top cochlea-enriched protein (Table S3), a finding of particular significance given that PANX3 is classified as a "missing protein" in neXtProt[27] (PE2, Extended Data Figure 2B). We further synthesized two unique peptides (LVQHMLK and YFEFPLLER) from PANX3 to confirm its presence and cochlear-specific expression (Extended Data Figure 2C) ..."

In revised SI:

*"...Besides the tissue enriched proteins mentioned in the main text, group enriched proteins also varies due to the tissues only in our dataset. For example, we found a protein grouped enriched in bone, bone marrow and osteocalcin named osteocalcin (BGLAP), which was classified into tissue enhanced proteins in choroid plexus and intestine in HPA (**Table S3**). BGALP, a 10.96 kDa protein of 100 amino acids, is mainly produced by osteoblasts[33]. The misclassification by HPA likely stems from the lack of bone-derived samples in their dataset, where BGALP shows low transcription across tissues (<10 transcripts per million), with relatively higher levels in the choroid plexus and intestine. Our database offers a resource for further exploration of bone's multifaceted biological roles..."*

The low concordance of tissue specificity annotations between our data and HPA RNA-seq is likely attributable to the low correlation observed, as described in SI.

…Multiple cross-tissue studies have shown that RNA–protein correlations across tissues are only moderate[6], and our data align with this trend, showing median Pearson correlations of 0.25 (HPA[7]) and

0.24 (GTEx[8]). Poorly RNA-correlated proteins mainly enriched in post-transcriptional regulation, including translational efficiency, protein complex and RNA splicing (SI, Table S3), which is consistent with previous study[5] ...

5. Biological insights and cancer findings

Many of the most compelling biological findings, particularly those from the paired carcinoma-adjacent analyses, are included in the supplementary materials. These results could significantly enhance the manuscript and deserve to be highlighted in the main figures. In contrast, the drug target investigation section receives considerable attention but remains largely conceptual or anecdotal. Additionally, mapping the cancer-specific proteins to the protein specificity categories would be valuable for further biological insights.

Reply: We appreciate the reviewer's insightful observation. We agree that the paired carcinoma-adjacent analyses provide more compelling and concrete insights into cancer biology. Specifically, we have added new analyses and reorganized the following results into the main figure panel (revised Figures 6).

"... Out of the DEPs across 25 tumor types, 2878 were tumor-specific, meaning they were significantly up- or down-regulated in only one specific tumor type (Figure 6A, Table S7). Hepatocellular carcinoma (HCC) had the most tumor-specific DEPs, followed by DLBCL and GIST, while esophageal carcinoma had the fewest. Most of the HCC-specific DEPs were downregulated and enriched in metabolic pathways (Table S7). Interestingly, synaptic signaling (GO: 0099536, $q = 3.72 \times 10^{-4}$) was enriched among the GIST-specific upregulated DEPs, consistent with the origin of GIST from interstitial cells of Cajal (ICCs), gut pacemaker cells that possess neuronal properties. Furthermore, KIT and ANO1, established makers for ICC[39,40], were identified as GIST-specific upregulated DEPs (Table S7), reflecting the specific cell-of-origin. Targeted MS-based quantification validated the cancer-specific upregulation of CPT1C and FXYD6 in GIST, both predominantly expressed in neurons (Figure 6B, Extended Data Figure 12), confirming the enhanced neuronal properties in GIST. Among these tumor-specific DEPs, 131 were tissue-enriched in the corresponding normal tissue, representing locally enriched DEPs (LEDEPs). Downregulated tumor-specific LEDEPs reflected the loss of specialized tissue functions, consistent with dedifferentiation in T inferred from the F-T-NT-N trend (Figure 2A). For example, RBP2 and PLS1 were only suppressed in the small intestine tumor (GIST); LIPF and GKN1 were specifically reduced in gastric carcinoma; and exocrine markers (e.g., CELA2A, CPA1, PNLIP) were uniquely downregulated in the pancreatic carcinoma (Figure 6G). Conversely, upregulated LEDEPs were predominantly observed in TGCT (Figure 6A), including TSPY2[41], suggesting the exploitation of intrinsic germ cell proliferative programs..."

"...Third, we aimed to minimize adverse effects by prioritizing tumor-enriched proteins that were significantly upregulated in tumors relative to both paired non-tumor tissues and all the normal tissues. We identified 41 unique tumor-enriched proteins located at the plasma membrane (Table S9, SI). Several are already targeted by clinically approved ADCs (e.g., CD79B[47]), supporting this prioritization strategy. The remaining candidates, particularly unexplored transmembrane proteins, represent novel therapeutic targets. TYROBP, a transmembrane signaling adaptor identified as tumor-enriched in ten tumor types (e.g., colorectal, gastric, renal and pancreas carcinoma, Figure 6D, Table S7), is a potential shared ADC target, though its myeloid expression requires toxicity evaluation. KIT, the established pacemaker of ICC, was identified as both GIST-specific and GIST-enriched (Figure 6E, Table S7), indicating the therapeutic potential of targeting aberrant ICC-like signaling in GIST. Targeted proteomic analysis confirmed PAX5 as tumor-specific and tumor-enriched in DLBCL (Figure 6C, 6F). PAX5 maintains B-cell identity, prevents terminal differentiation, and contributes to lymphomagenesis through sustained transcriptional

activation[48]*. Recent evidence further shows that PAX5 inhibition enhances the efficacy of BTK blockade in DLBCL preclinical models*[49]*, highlighting its potential as a therapeutic target in this malignancy."*

References

1 Chang, K. *et al.* The Cancer Genome Atlas Pan-Cancer analysis project. *Nature genetics* **45**, 1113-1120, doi:10.1038/ng.2764 (2013).

2 Ellis, M. J. *et al.* Connecting genomic alterations to cancer biology with proteomics: the NCI Clinical Proteomic Tumor Analysis Consortium. *Cancer discovery* **3**, 1108-1112, doi:10.1158/2159-8290.Cd-13-0219 (2013).

3 Heath, A. P. *et al.* The NCI Genomic Data Commons. *Nat Genet* **53**, 257-262, doi:10.1038/s41588-021-00791-5 (2021).

4 Gonçalves, E. *et al.* Pan-cancer proteomic map of 949 human cell lines. *Cancer Cell* **40**, 835-849.e838, doi:10.1016/j.ccell.2022.06.010 (2022).

5 Nusinow, D. P. *et al.* Quantitative Proteomics of the Cancer Cell Line Encyclopedia. *Cell* **180**, 387-402.e316, doi:10.1016/j.cell.2019.12.023 (2020).

6 Liu, Y., Beyer, A. & Aebersold, R. On the Dependency of Cellular Protein Levels on mRNA Abundance. *Cell* **165**, 535-550, doi:10.1016/j.cell.2016.03.014 (2016).

7 Uhlén, M. *et al.* Proteomics. Tissue-based map of the human proteome. *Science* **347**, 1260419, doi:10.1126/science.1260419 (2015).

8 The GTEx Consortium atlas of genetic regulatory effects across human tissues. *Science* **369**, 1318-1330, doi:10.1126/science.aaz1776 (2020).

9 Zhu, Y. *et al.* High-throughput proteomic analysis of FFPE tissue samples facilitates tumor stratification. *Molecular Oncology* **13**, 2305-2328, doi:https://doi.org/10.1002/1878-0261.12570 (2019).

10 Achter, J. S. *et al.* Quantitative proteomics of formalin-fixed, paraffin-embedded cardiac specimens uncovers protein signatures of specialized regions and patient groups. *Nature Cardiovascular Research* **4**, 1409-1423, doi:10.1038/s44161-025-00721-2 (2025).

11 Jiang, L. *et al.* A Quantitative Proteome Map of the Human Body. *Cell* **183**, 269-283.e219, doi:10.1016/j.cell.2020.08.036 (2020).

12 Ding, Y. *et al.* Comprehensive human proteome profiles across a 50-year lifespan reveal aging trajectories and signatures. *Cell* **188**, 5763-5784.e5726, doi:10.1016/j.cell.2025.06.047 (2025).

13 Wang, D. *et al.* A deep proteome and transcriptome abundance atlas of 29 healthy human tissues. *Molecular systems biology* **15**, e8503, doi:10.15252/msb.20188503 (2019).

14 Wen, B. *et al.* Assessment of false discovery rate control in tandem mass spectrometry analysis using entrapment. *Nat Methods* **22**, 1454-1463, doi:10.1038/s41592-025-02719-x (2025).

15 Liang, S. *et al.* Population-based metaproteomics reveals functional associations between gut microbiota and phenotypes. *bioRxiv*, 2024.2011.2026.625569, doi:10.1101/2024.11.26.625569 (2024).

16 Gillet, L. C. *et al.* Targeted data extraction of the MS/MS spectra generated by data-independent acquisition: a new concept for consistent and accurate proteome analysis. *Molecular & cellular proteomics : MCP* **11**, O111.016717, doi:10.1074/mcp.O111.016717 (2012).

17 Demichev, V., Messner, C. B., Vernardis, S. I., Lilley, K. S. & Ralser, M. DIA-NN: neural networks and interference correction enable deep proteome coverage in high throughput. *Nature Methods* **17**, 41-44, doi:10.1038/s41592-019-0638-x (2020).

18 Laatsch, C. N. *et al.* Human hair shaft proteomic profiling: individual differences, site specificity and cuticle analysis. *PeerJ* **2**, e506, doi:10.7717/peerj.506 (2014).

19 Parker, G. J. *et al.* Demonstration of Protein-Based Human Identification Using the Hair Shaft Proteome. *PLoS One* **11**, e0160653, doi:10.1371/journal.pone.0160653 (2016).

20 Goecker, Z. C. *et al.* Optimal processing for proteomic genotyping of single human hairs. *Forensic science international. Genetics* **47**, 102314, doi:10.1016/j.fsigen.2020.102314 (2020).

21 Fröhlich, K. *et al.* Benchmarking of analysis strategies for data-independent acquisition proteomics using a large-scale dataset comprising inter-patient heterogeneity. *Nat Commun* **13**, 2622, doi:10.1038/s41467-022-30094-0 (2022).

22 Lou, R. *et al.* Benchmarking commonly used software suites and analysis workflows for DIA proteomics and phosphoproteomics. *Nat Commun* **14**, 94, doi:10.1038/s41467-022-35740-1 (2023).

23 Staes, A. *et al.* Benefit of In Silico Predicted Spectral Libraries in Data-Independent Acquisition Data Analysis Workflows. *Journal of proteome research* **23**, 2078-2089, doi:10.1021/acs.jproteome.4c00048 (2024).

24 Zhu, Y., Aebersold, R., Mann, M. & Guo, T. SnapShot: Clinical proteomics. *Cell* **184**, 4840-4840.e4841, doi:10.1016/j.cell.2021.08.015 (2021).

25 Geffen, Y. *et al.* Pan-cancer analysis of post-translational modifications reveals shared patterns of protein regulation. *Cell* **186**, 3945-3967.e3926, doi:10.1016/j.cell.2023.07.013 (2023).

26 Li, Y. *et al.* Pan-cancer proteogenomics connects oncogenic drivers to functional states. *Cell* **186**, 3921-3944.e3925, doi:10.1016/j.cell.2023.07.014 (2023).

27 Li, Y. *et al.* Proteogenomic data and resources for pan-cancer analysis. *Cancer Cell* **41**, 1397-1406, doi:10.1016/j.ccell.2023.06.009 (2023).

28 Wilhelm, M. *et al.* Mass-spectrometry-based draft of the human proteome. *Nature* **509**, 582-587, doi:10.1038/nature13319 (2014).

29 Ma, W. *et al.* DreamAI: algorithm for the imputation of proteomics data. *bioRxiv*, 2020.2007.2021.214205, doi:10.1101/2020.07.21.214205 (2021).

30 Sanchez-Vega, F. *et al.* Oncogenic Signaling Pathways in The Cancer Genome Atlas. *Cell* **173**, 321-337.e310, doi:10.1016/j.cell.2018.03.035 (2018).

31 Jesse, K. Rtsne: T-Distributed Stochastic Neighbor Embedding using a Barnes-Hut Implementation. *CRAN: Contributed Packages*, doi:10.32614/cran.package.rtsne (2014).

32 Battle, A., Brown, C. D., Engelhardt, B. E. & Montgomery, S. B. Genetic effects on gene expression across human tissues. *Nature* **550**, 204-213, doi:10.1038/nature24277 (2017).

33 Zoch, M. L., Clemens, T. L. & Riddle, R. C. New insights into the biology of osteocalcin. *Bone* **82**, 42-49, doi:10.1016/j.bone.2015.05.046 (2016).

Referee #1
Remarks to the Author:

This revised version of the manuscript addresses adequately the major points mentioned in my previous review. As key points, the methodology and the uniqueness of this study (when compared with previous ones) are much clearer. Overall, the information that the manuscript contains is extensive, and naturally, the authors can only show some highlights from all the generated data.

For this reason, I think the authors should improve the reusability of such valuable dataset for the community, since I anticipate that this dataset will be heavily reused in the future. For this purpose, I suggest that the authors provide an accurate metadata annotation of the dataset, by showing the links between the raw files and the samples (for instance, by creating a SDRF-Proteomics file, or several ones, and making it available in the dataset submitted to ProteomeXchange/PRIDE).

Reply: We thank the reviewer for this insightful suggestion. In response, we have generated a comprehensive metadata annotation file following the Sample and Data Relationship Format (SDRF-Proteomics) standard. This file explicitly maps each raw mass spectrometry file to its corresponding biological sample, including clinical parameters and experimental conditions. We have uploaded the SDRF file (sdrf_validated.txt) to the ProteomeXchange/PRIDE repository (Project ID: PXD063370).

We have updated the "Data Availability" section in the revised manuscript to reflect these additions.

"Raw data as well as corresponding metadata annotations following the Sample and Data Relationship Format (SDRF-Proteomics) standard have been deposited to the ProteomeXchange Consortium (https://proteomecentral.proteomexchange.org) via the iProX[96,97] (PXD077178, IPX0003578000) and the PRIDE[98,99] repository (PXD063370)."

Also, the tissues under study do not seem to be properly annotated in the dataset (as everything is annotated as 'whole body', which is not correct). Furthermore, the submitted dataset does not seem to contain (at least as far as I could see) the spectral library used for the analysis and the corresponding DDA generated data for constructing the PASEF spectral library. I only could see DIA and PRM related data. These data should be provided e.g. in a different linked dataset, and would enable the reproducibility of the reported results. Just for clarification, I accessed the dataset via PRIDE as a reviewer.

Reply: We thank the reviewer for this careful comment. In the PRIDE repository, "whole body" was selected as the 'TISSUE' label of this project to provide a concise overarching summary of the comprehensive sample collection across the human body. Since the 'TISSUE' tag often requires few representative terms for the entire project, "whole body" was considered more appropriate than an exhaustive list of individual tissues. This is not ideal but this is the limitation of the current PRIDE annotation system. To ensure granular transparency

and reproducibility, we have provided a comprehensive SDRF file. This file contains precise annotations for each raw data file, including specific tissue types, biological replicates, and technical parameters.

We have ensured that all raw DDA data files used for library construction, along with the generated PASEF spectral library (provided as library.tsv), are fully accessible via the PRIDE/iProX repositories.

An extra detail is that neXtProt is mentioned as an active database, but it is no longer active, and the latest release was done in September 2023 (see https://www.expasy.org/archives/nextprot). UniProtKB/SwissProt is the best alternative, and any claims on missing proteins should be confirmed and attributed to UniProt.

Reply: We thank the reviewer for this critical correction regarding the database status. We acknowledge that UniProtKB/Swiss-Prot is the gold standard for defining protein existence (PE) levels. Following the reviewer's suggestion, we have replaced neXtProt with the latest version of UniProtKB/Swiss-Prot to define our "missing protein" candidates, specifically focusing on human-reviewed proteins with PE levels 2–4. However, due to the updated classification in UniProt, PANX3 is no longer categorized as a missing protein. Consequently, we have removed all claims regarding the "discovery of a missing protein" for PANX3 from the Results section. However, PANX3 remains an under-studied protein with significant biological interest. While the Human Protein Atlas (HPA) labels it as "not detected" and Bgee lists its highest expression in the tibia (notably, the cochlea is absent from the Bgee database, https://www.bgee.org/gene/ENSG00000154143), our study provides robust evidence of its existence and high tissue-specificity in the cochlea. We believe these findings still offer valuable insights into the understudied proteins, even as the protein’s existence status has evolved.

“Among these, we identified PANX3 as the top cochlea-enriched protein (Table S4), which is currently documented as 'not detected' in the HPA[8] dataset. We further synthesized two unique peptides (LVQHMLK and YFEFPLLER) from PANX3 to confirm its presence and cochlear-specific expression (SI, Table S2).”

Besides, GPR21 and GPR22, which were previously classified as missing proteins in neXtProt and were identified and validated using synthetic peptides in our study, are no longer characterized as “missing proteins”, either. In light of this, we have streamlined the manuscript by removing the text and figures related to ‘missing protein’ (previously in the SI and Methods). We have confirmed that this removal does not impact the primary conclusions or the integrity of our core datasets. We are currently planning a separate, more comprehensive study specifically dedicated to the deep mining and experimental validation of the remaining under-studied and missing protein candidates.
