# Supplementary material for: Spatial distribution of the proteome in the human body and in cancers
Source: Nature. 2026 Jun 17;656(8126):227–36. doi: 10.1038/s41586-026-10660-y (PMC13442039; doi:10.1038/s41586-026-10660-y)
Supplement: Supplementary file 2 [file 41586_2026_10660_MOESM1_ESM.pdf]

nature portfolio

https://doi.org/10.1038/s41586-026-10660-y

Supplementary information

Spatial distribution of the proteome in the human body and in cancers

In the format provided by the
authors and unedited

Supplementary information

Supplementary Table Legends

Supplementary Table 1. Sample origin information.

A. Information of non-carcinoma originated samples. The identifier, age, gender and disease of patients or volunteers, and the storage method of biological specimens have been listed. **B.** Information of cancer patient originated samples. The identifier, age, gender, tissue name, anatomical classification and corresponding tissue specific library name, and the origin center of patients have been listed. **C.** Statistical results of cancer patients. We are including patient number, gender ratio, mean and standard deviation of ages for each kind of cancers. **D.** Sample information including both biological and technical variates for each MS run.

Supplementary Table 2. DDA data identifications

A. Identification of proteins in tissue-specific libraries. **B**. Contaminant library. **C.** Protein groups specifically detected in the DDA-based spectral library of the cochlea compared with other normal tissues.

Supplementary Table 3. Proteome transformation in tissue development and oncogenesis.

A. Dimensional reduction of proteins across fetal (F), tumor (T), non-tumor (NT) and normal (N) samples using t-SNE. **B.** Co-expression modules of trajectory-associated proteins.

Supplementary Table 4. Tissue specificity.

A-B. Correlation, coefficient of variation (CV) and Euclidean distance within each of the major tissue types (**A**), and between a specific tissue type and other tissue types within the same major tissue (**B**) before adjustment of tissue types and deletion of heterogeneity abnormal samples. Ranked by a decreasing order of median distance. **C-D,** CV and Euclidean distance within each of the major tissue types (**C**) and detailed tissue types (**D**) after adjustment of tissue types and deletion of heterogeneity abnormal samples. **E.** Median protein intensities in various tissue types. **F.** Classification of tissue specificity for each protein. **G**. Distribution of protein-RNA correlation values. **H**. GOBP enrichment of negatively correlated protein-RNA (B-H adjusted $p < 0.1$, correlation < 0) between this study and HPA. **I.** Pearson's *r* of overlapped proteins for each overlapped tissue among our data and the two previously published human proteome drafts[1,2].

Supplementary Table 5. Druggable tissue-enriched proteins information.

A. Intensity matrix of tissue-enriched proteins (Z-score). **B.** Comparison of tissue-enriched proteins with external tissue specificity databases. **C.** Mapping of tissue-enriched proteins to

the DrugBank drug target database. **D.** Integrated results organized by proteins. **E.** Statistics of drug approval status. **F.** Statistics of drug mechanism actions. **G.** Intensity matrix of the top 5 enriched proteins associated with at least three drugs. **H-K.** Subsets of data from **B**, **D**, **E**, and **F** corresponding to the proteins selected in **G**. **L.** Comparative functional profiling of tissue types using tissue-enriched proteins based on GOBP enrichment. **M.** Filtered GOBP terms from **L**, annotated with their generality (number of tissue types represented in each enriched term). **N.** Network relationships of the selected GOBP terms.

Supplementary Table 6. Differential expression analysis.

A. Results of differential expression analysis between paired tumor and non-tumor samples for each cancer type. **B.** Differentially expressed proteins filtered by |Hedges' g| not less than 0.5 and B-H adjusted $p < 0.05$.

Supplementary Table 7. Cross-reference to external cancer datasets.

A. Overlap of our data, TCGA, and CPTAC dysregulated proteins datasets. B. Differentially expressed proteins matched to the HPA pathology dataset. **C**. Overlap of **A** and **B**. D. Statistics of **B**. **E.** Drug-DEP matches between DrugBank-recorded biotechnology drugs and cancer DEPs. **F.** Biotechnology drug-cancer-DEP associations linked to relevant clinical trials.

Supplementary Table 8. Cancer-specific proteins.

A. Tumor-specific dysregulated proteins. **B.** Locally enriched dysregulated proteins with their enriched tissues, median expression intensity, and results of differential expression analysis. **C.** Tumor-enriched DEPs. **D.** Tumor-enriched DEPs localized to the plasma membrane. **E**. Enrichment results of HCC specifically downregulated DEPs by Metascape. **F**. Enrichment results of GIST specifically upregulated DEPs by Metascape.

Supplementary Table 9. Cancer DEPs associated pathways and drugs.

A. DEPs detected in TCGA curated pathways. B. DEPs validated in ProCan-DepMapSanger proteomic map drug response dataset. C. DEPs validated in ProCan-DepMapSanger proteomic map CRISPR-Cas9 gene essentiality dataset.

Supplementary results

Anatomically dissectible resolution of human sample collection

In the sample repository, we collected histological healthy tissues mainly from a male and a female participant (**Supplementary Fig. 1**) at an anatomical resolution. In total, we collected 58 roughly partitioned tissue types, among which 24 were newly added compared with HPA[3] and TSomics[1] (**Supplementary Fig. 1**), and 251 detailed tissue types, of which 212 were newly added (**Supplementary Fig. 1**). For in-situ tumor and its paired non-tumor samples, we recruited 22-90 patients for each of the 25 cancer types (**Table S1**). To handle the various tissue types in our collection,

we applied the pressure cycling technology (PCT)-assisted sample preparation[4,5] along with in-solution digestion and in-gel digestion method to extract proteins and digested them into peptides for the following data-dependent acquisition (DDA) and data-independent acquisition (DIA) analysis.

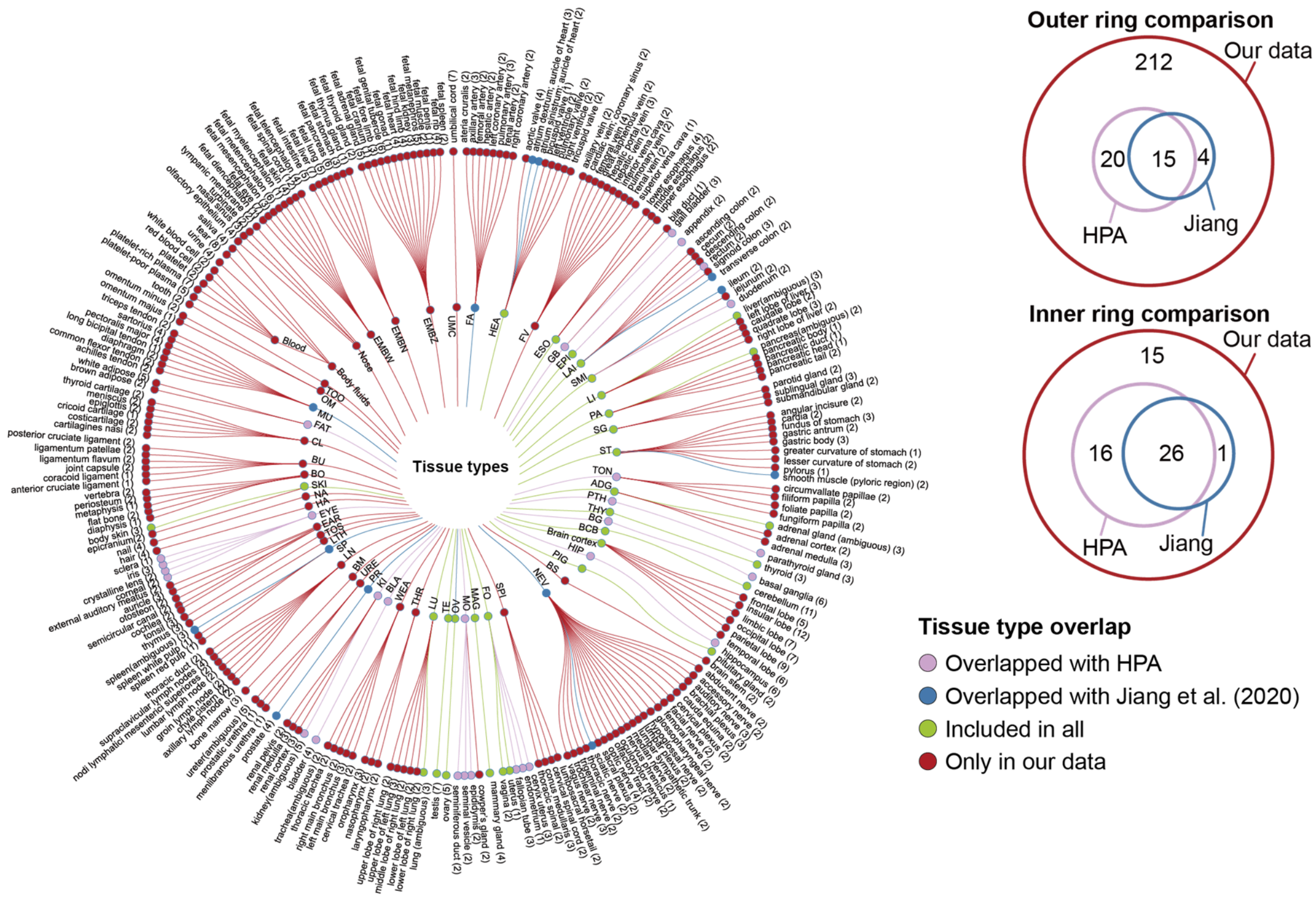

Supplementary Figure 1. Overview of sample collection. Tissue type overlap between this study and other published datasets. Numbers in the brackets represent the number of files in each detailed tissue type.

Construction of the comprehensive spectral library

To comprehensively quantify the proteome of human samples, we first built a “dictionary” — the MS spectral library on timsTOF Pro MS using the DDA method, by analyzing pre-fractionated peptide sample pools per tissue type (**Extended Data Fig. 1a, Table S2**). Altogether, we analyzed 1028 DDA files from 65 types of human specimens and generated 65 tissue-specific sub-libraries using FragPipe[6-9]. As shown in **Extended Data Fig. 1c**, the testis tissue-specific library contains the highest number of proteins (8929 proteins), while the tooth library has the fewest (1316 proteins). By leveraging all the DDA files, the generated library contains 22,539,157 peptide spectrum matches (PSMs), leading to the identification of 15,332 proteins from 484,615 peptides. To ensure the quality of the spectral library, we evaluated the characteristics of the comprehensive library as shown in **Supplementary Fig. 2A to 2K**. The majority of precursor m/z values were settled between 300-1000 Th, with 2+ and 3+ precursors accounting for over 90% of the total. Meanwhile, these precursors exhibited a strong correlation in their chromatographic characteristics. Almost all the proteins have over six razor or unique peptides, and almost every precursor has over six fragments. Most proteins in the dataset have protein coverage exceeding 20%. Our results showed a higher median protein coverage compared to the proteome map[10] (33.43% versus 28%).

We also compared the comprehensive library with two of the largest human peptide-based resources, PeptideAtlas[11] and ProteomicsDB[12]. PeptideAtlas contains curated peptide information collected from the proteomics community over the past decade, while ProteomicsDB achieved 92% proteome coverage by integrating about 17,000 human proteomic profiles. Notably, almost half of the peptides we identified were not deposited in ProteomicsDB and still discovered over 70,000 novel peptides and 449 novel proteins not recorded either in the PeptideAtlas or ProteomicsDB (**Supplementary Fig. 2L, 2M**). It is reasonable to attribute the expansion in protein identification to the breadth and depth of our proteomic analysis because we expanded the diversity of tissue types compared to previous studies.

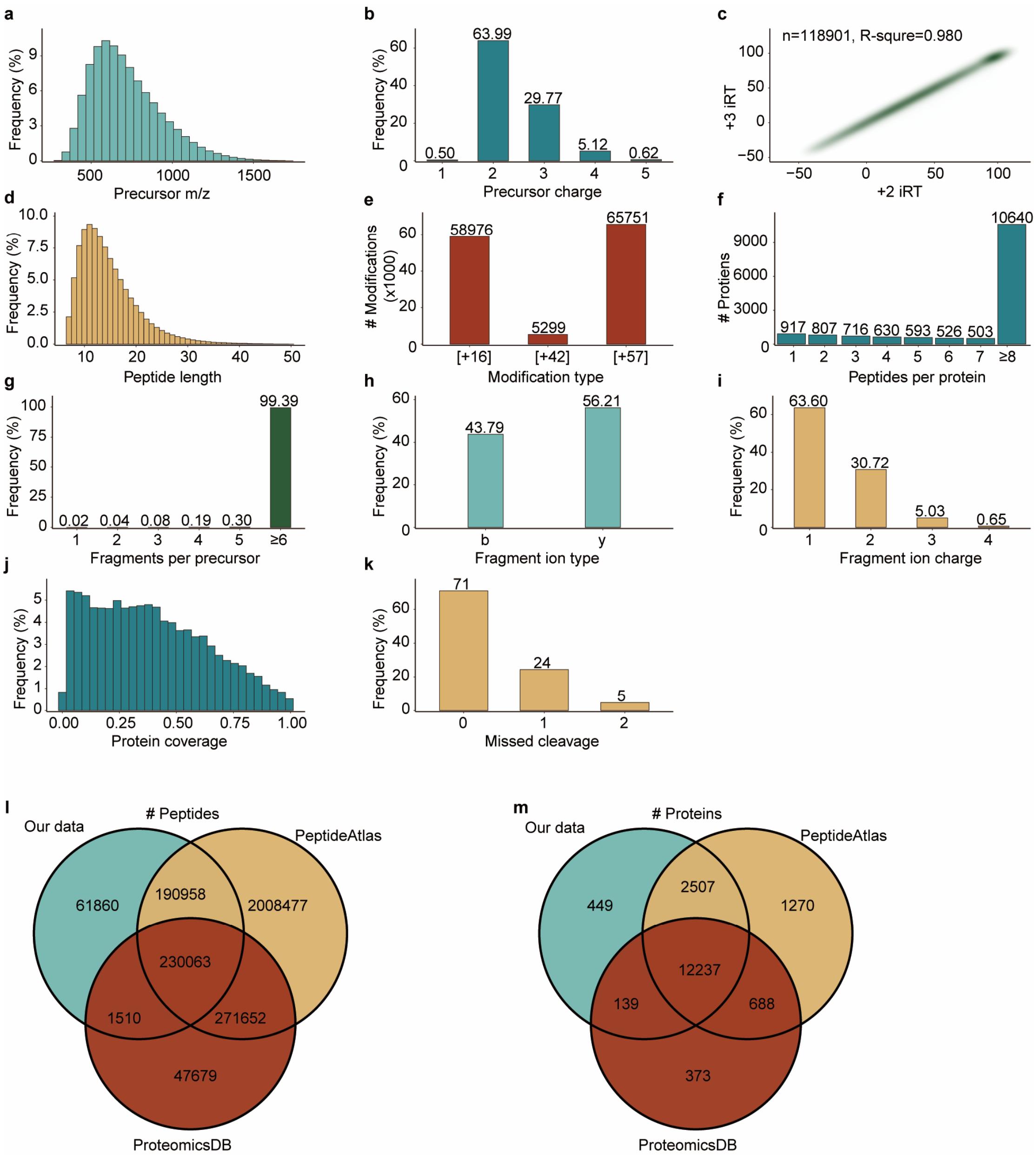

Supplementary Figure 2. Overview of the DDA-based spectral library. Histograms showing the distribution of precursor m/z (**a**) and charge (**b**) of the spectral library. **c**, Pearson correlation of iRT values between 2+ and 3+ precursor ions with the same peptide sequence. Histograms showing the

frequency percentages of peptide length (**d**), number of modifications (**e**), peptides per protein (**f**), fragments per precursor (**g**), fragment ion type (**h**), fragment ion charge (**i**), protein coverage (**j**) and number of missed cleavages per peptide (**k**). Overlapped peptides (**l**) and proteins (**m**) Amongour data, PeptideAtlas, and ProteomicsDB datasets.

Assessment of false discovery rate (FDR) control in DIA-MS using entrapment

To rigorously assess and control false discoveries, we implemented a combined entrapment strategy by constructing a hybrid spectral library that contained empirical human-derived spectra from the project-specific DDA library together with decoy spectra generated from non-human species[13]. This design enabled a direct evaluation of identification specificity at both the precursor and protein group levels. Entrapment PSM and precursor were detected at a low level, representing less than 1% of the False Discovery Proportion (FDP) at a 1% *q* value cutoff (**Supplementary Fig. 3**). However, the FDP exceeded 2% at the protein level. To control the protein FDR to less than 2%, we subsequently adjusted the *q* cutoff to 0.1% (**Supplementary Fig. 3, Methods**).

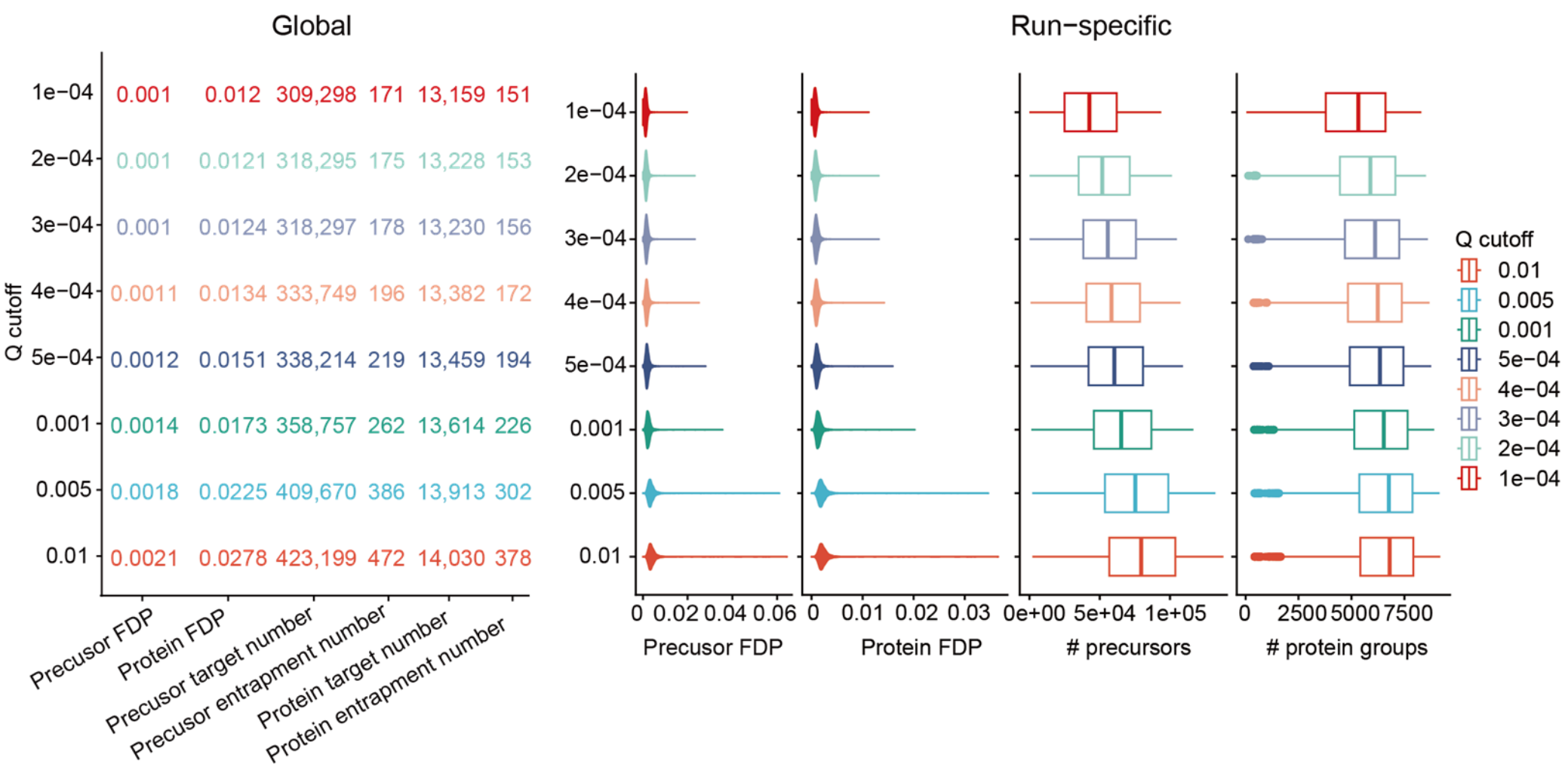

Supplementary Figure 3. Effect of DIA-NN q-value cutoffs on entrapment-library search performance. Global panels (left) summarize, for each q-value cutoff, precursor- and protein-group-level false discovery proportions and corresponding global identification counts for precursor targets, precursor entrapments, protein-group targets, and protein-group entrapments. Run-specific panels (right) display, across individual runs and q-value cutoffs, the distributions of Precursor FDP, Protein FDP, the number of identified precursors (# precursors), and the number of identified protein groups (# protein groups); violin plots depict density distributions, and box plots (with colors denoting q-value cutoff) indicate median, first quartile (Q1), third quartile (Q3), and lower outliers defined as Q1 − 1.5×IQR. For distribution plots (FDP and identification counts), n = 3262 independent MS files were analyzed for each q-value cutoff, except for q = 1e-04 where n = 3261 (one run yielded no identifications at this stringent threshold).

Data quality of the quantitative proteomic analysis

In summary, we used PCT to ensure consistency in sample preparation for most sample types except

for urine, saliva, and plasma, which were prepared by a two-step overnight enzyme digestion method (**Extended Data Fig. 1b**). During the sample preparation process, a subset of 2555 samples was randomly selected as biological replicates (n = 33), resulting in a total of 2588 peptide samples for DIA analysis. During the data acquisition process, a subset of samples was randomly selected for multiple injections (n = 235), and a unified pooled peptide sample was multiple-injected before each batch (n = 149). In total, 3005 DIA mass spectrometry files were generated. We used the comprehensive spectral library and DIA-NN to analyze these 3005 raw DIA files, resulting in a quantitative matrix of 13,609 proteins (**Fig 1A**，**Extended Data Fig. 1b**).

Protein and peptide identification showed significant differences in different tissue types (**Extended Data Fig. 1c**). The number of proteins and peptide segments also varied within the same tissue type, possibly due to the structural and functional heterogeneity within the tissue type and the presence of cancer involvement. Generally, the number of protein identifications in Tumor (T) and Non-tumor (NT) samples is higher than that in Normal (N) samples within the same tissue type (**Extended Data Fig. 1c**). Among all samples, the maximum protein identification of a single-shot DIA was found in an ovarian cancer sample (8900 proteins), while the minimum protein identifications were found in plasma (a mean of 715 ± 134 proteins, n = 13) and lens samples (777 and 875 proteins, n = 2).

Next, we performed quality control (QC) analysis on the quantification results of the obtained protein matrix. First, files with low protein identifications were removed. Specifically, a file was excluded if its protein ID count was below the lower outlier (1.5 times the interquartile range below the first quartile) for its specific combination of major and detailed tissue type, and sample type. A file was filtered out only if it met this lower outlier criterion for both its major and detailed tissue type thresholds. This strict filtering excluded 48 files. Second, we removed the proteins not detected in over half of the samples in any combination of sample type and detailed tissue type, leading to a reduction of protein identification to 12,797 in all samples.

Then, we performed batch correction and evaluated the batch effect by Principal Variance Component Analysis (PVCA). As shown in Extended Data Fig. 1f, biological factors account for ~66% of total variance, with tissue type being the largest contributor (50.94%). Technical factors collectively explain ~4% in all, with batch plus major tissue type contributing 1.49% and sample collection batch plus major tissue type contributing 1.33%. Other technical variables were below 1% each. Compared with the uncorrected matrix, the ratio of variance attributed to independent biological effects relative to mixed technical effects increased from ~13.7 to ~15.6 (**Extended Data Fig. 1f**, **Supplementary Fig. 4**). This demonstrates that the observed proteomic structure is primarily driven by biology rather than technical noise.

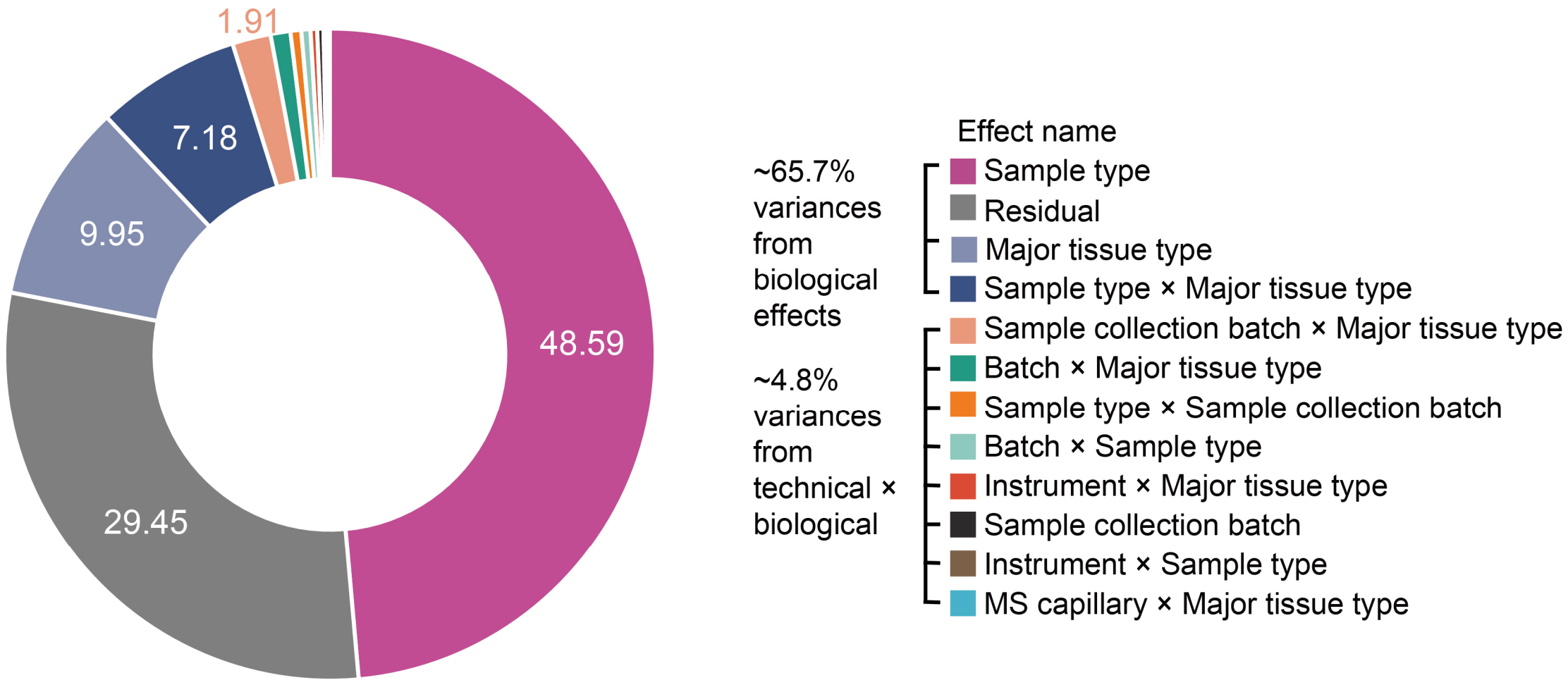

Supplementary Figure 4. Batch effect estimation. PVCA results for the uncorrected proteomic matrix. The pie chart displays the proportion of variance attributed to major experimental, biological, and interaction effects analyzed by PVCA, including batch, tissue type, and their interactions.

Before calculating the CVs and the correlation of the pooled sample files and replicates, we removed low identification pools and replicates first. One pool and 15 technical replicates were removed. This removed pool sample had a supplementary injection in the same batch. We found that most CVs of the protein abundances in the pooled peptide samples and replicates were low, with median values of 0.18 in pools (n = 148), 0.05 in technical replicate pairs (n = 218), and 0.06 in biological replicate pairs (n = 33), respectively (**Extended Data Fig. 1d**). Most of the correlation coefficients of protein abundances among the pooled samples and the replicates were high, with a median Pearson's correlation coefficient of 0.85 in pools (n = 148), 0.92 in technical replicates (n = 218), and 0.90 in biological replicates (n = 33), respectively (**Extended Data Fig. 1e**). Besides, we filtered a total of 2239 tumor and non-tumor clinical samples, as well as companion pooled peptide samples (**Extended Data Fig. 2a**). We evaluated the reproducibility and batch effect in the pan-cancer proteomic matrix. We found that most CVs of the protein abundances in the 92 pooled peptide samples, 163 pairs of technical replicates, and 13 pairs of biological replicates were low, with median values of 0.18, 0.06, and 0.07, respectively (**Extended Data Fig. 2c**). Most of the correlation coefficients of protein abundances among pooled peptide samples and replicates were high, with a median Pearson's correlation coefficient of 0.86 for pools, 0.92 for technical replicates, and 0.91 for biological replicates (**Extended Data Fig. 2d**).

In summary, QC analysis indicated excellent reproducibility and minimal batch effects within our dataset, assuring subsequent data analysis.

Contaminant protein library

We observed over 2000 proteins identified in four hair samples. Overall, the hair proteome is dominated by highly abundant keratin and filament proteins (**Supplementary Fig. 5A**). However, most hair proteomics studies have identified only a few hundred proteins[14,15]. A recent large-scale study[16] (130 samples) identified ~5000 proteins in all samples, likely reflecting both sample heterogeneity and improved detection methods (**Supplementary Fig. 5B**). Hair proteins identified by this study were also

enriched in blood-related pathways, suggesting possible blood contamination (**Supplementary Fig. 5C,5D**).

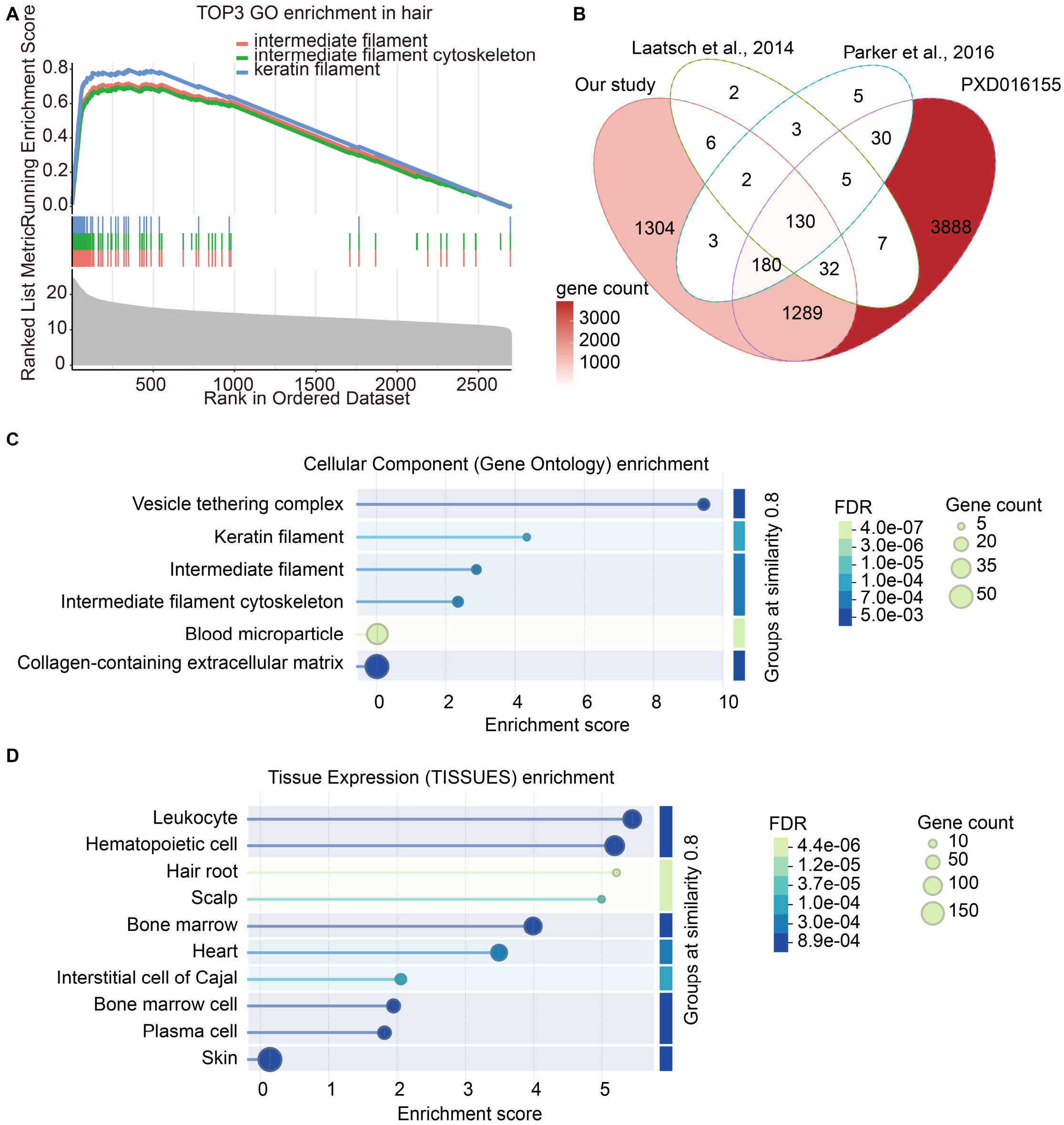

Supplementary Figure 5. Hair proteome composition. A, Gene set enrichment analysis of the average intensity of hair proteome, highlighting the top three enriched Gene Ontology Cellular Component terms. **B,** Venn diagram showing the overlap of identified hair proteins between our study and published datasets. Numbers indicate protein counts for each subset. **C-D,** Over-representation analysis of proteins uniquely identified in our study using Gene Ontology Cellular Component (**C**) and Tissue Expression (TISSUES) database (**D**). Enrichment analysis was performed using the 'Proteins with Values/Ranks' module on STRING (https://cn.string-db.org/). In the dot plots, the x-axis represents Enrichment Score, dot size scales with the number of genes associated with each term (gene count), and dot color represents the FDR as indicated in the color scale. Statistical significance was determined using hypergeometric or Fisher's exact test with Benjamini-Hochberg (B-H) correction; all

shown terms meet a threshold of FDR < 0.05.

Analysis of blank controls indicated residual high-abundance blood and intracellular proteins in the LC-MS system, as hemoglobin, actin, and GAPDH were consistently detected (**Supplementary Fig. 6A, Table S2**). These proteins can be regarded as background contaminants, and we are expanding a contamination library to further mitigate their impact on pre-test tissue-specific analyses. Contaminant proteins were predominantly detected in body fluid, hair, and nail samples (**Supplementary Fig. 6B**). To ensure robust tissue-specific profiling, these tissue types were excluded from downstream tissue specificity analyses. Additionally, contaminant proteins identified in other tissue types will be filtered out using the updated contamination library.

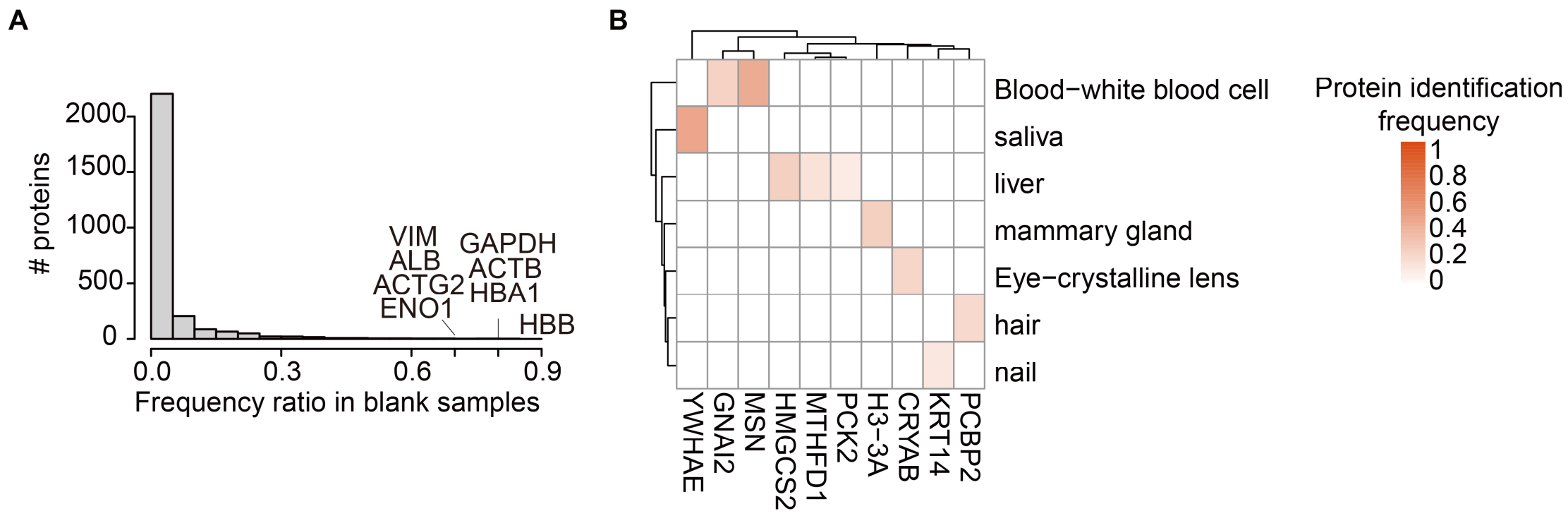

Supplementary Figure 6. Background contaminants detected in blank samples. A, Histogram of protein detection frequency in LC–MS/MS blank controls, proteins repeatedly detected across blanks. **B,** Heatmap showing tissue-enriched representative contaminant proteins across sample types. Color indicates the percentage frequency of detection in blanks for each protein displayed.

Tissue heterogeneity analysis for normal samples

Intra-type heterogeneity was detected across all normal tissue categories. By the original major tissue type classification, all normal samples were classified into 59 major tissue types (further separating blood into blood cell and plasma, versus the 58 tissue types shown in Supplementary Fig.1). Tissue types with the highest median distance within tissue type were eye, blood cell, fallopian tube, vagina, pancreas and throat (**Table S4**). This high intra-heterogeneity could be due to different subtypes within the same major tissue type having distinct proteomes. For example, eye samples exhibited high internal complexity: distinct anatomical components (crystalline lens, cornea, iris, sclera) displayed markedly different proteomic profiles, and the distance between the crystalline lens and to iris and cornea is among the top 10 distances of detailed tissues within the same major tissue type (median distance: 538.9 and 395.22, **Table S4**), supporting their treatment as separate tissue entities for specificity analysis. Cartilage demonstrated substantial subtype heterogeneity, particularly for epiglottis (**Table S4**). Hematoxylin and Eosin (H&E) staining showed histological features of epiglottis distinct from hyaline and fibrocartilage (**Supplementary Figure 7B, 7C**), so we also took it as a separate tissue for specificity analysis. Sampling heterogeneity contributed additional variance. For example, thyroid cartilage from donor DL3 contained mixed tissue types, whereas DL4 was predominantly cartilaginous (**Supplementary Fig. 7C; Table S4**). The nasopharynx was observed to be enriched in glandular cells,

while the oropharynx had fewer glandular cells but more connective tissues (**Supplementary Fig. 7D**). Although vaginal tissue exhibits substantial histological heterogeneity, examination of H&E-stained sections from the sampled regions confirmed that the collected specimens were morphologically intact and representative (**Supplementary Fig. 7A).** Within the vagina group, sample DIA_554 showed an anomalous proteomic profile most similar to testis based on median Euclidean distance（172.21) and Pearson correlation (0.92); because it was processed adjacent to a testis sample (DIA_555), cross-contamination is a plausible explanation. Accordingly, DIA_554 was classified into the tissue heterogeneity abnormal group (**Table S1)** and excluded from the tissue specificity analysis. In the fallopian tube, H&E staining indicated that epithelial thickness varied across patients, reflecting inter-individual structural differences within this tissue type (**Supplementary Fig. 7E**). In pancreatic tissue, H&E examination showed complete autolytic necrosis in the sample from donor DL4, contrasting sharply with donor DL3, and this sample-specific degradation likely accounts for the corresponding proteomic deviation (**Supplementary Fig. 7F**). Thus, pancreas samples from DL4 were removed before downstream analysis. Similarly, we checked other high heterogeneity categories and labeled abnormal samples or separated major categories to make the intra-tissue samples exhibit significantly higher correlation and lower proteomic distances than inter-tissue comparisons ($p < 0.001$; **Extended Data Fig. 3b-e**, **Table S4**).

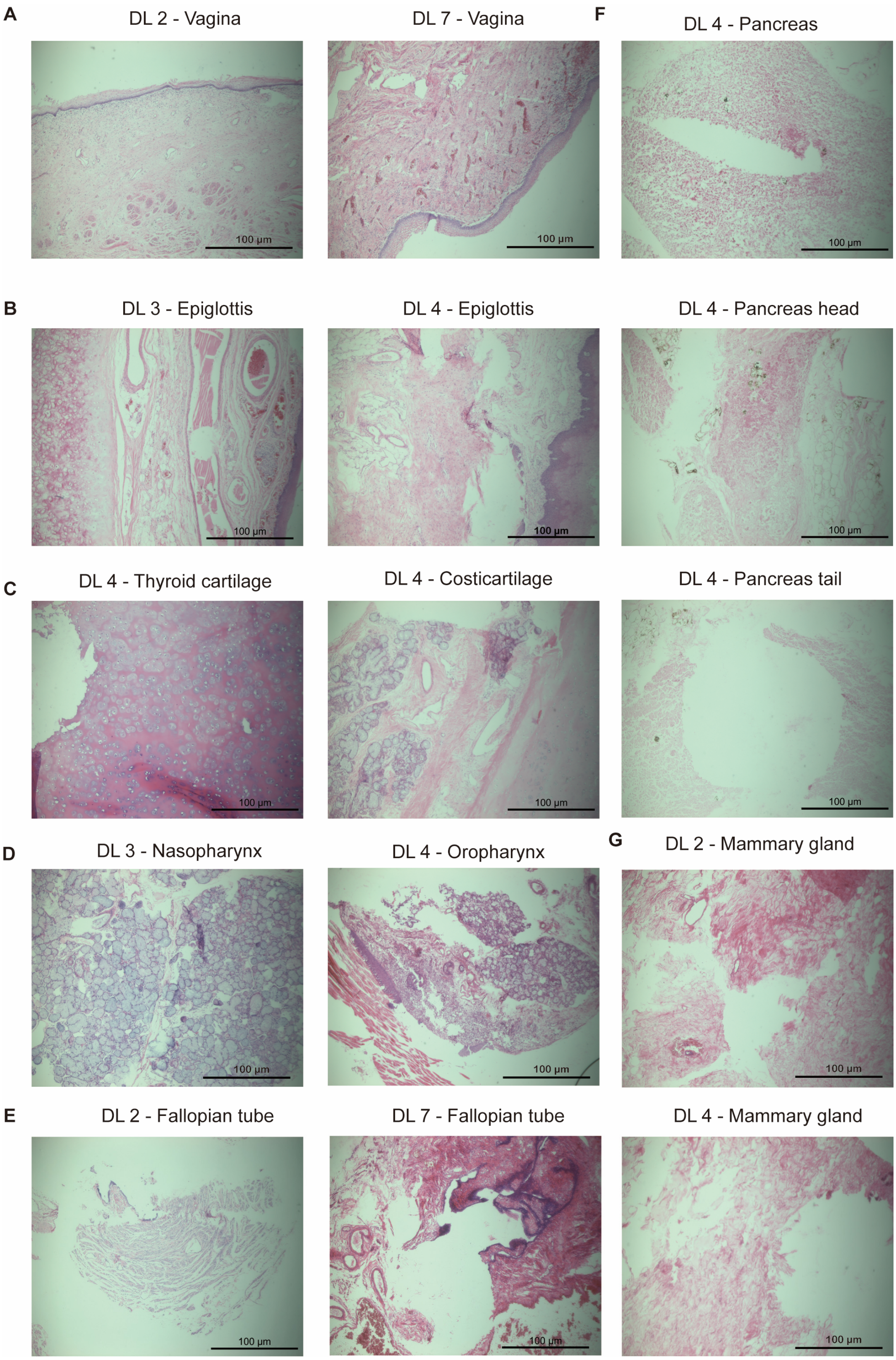

Supplementary Figure 7. HE staining of tissue heterogeneity in abnormal samples. Hematoxylin and Eosin (H&E) stained sections surrounding the tissue punch used for proteomic analysis were shown for tissues with top ranked median Euclidean distance and mammary gland. **(A)** Vagina of DL 2

and DL 7 participants. **(B)** Epiglottis from DL 3 and DL 4. **(C)** Thyroid cartilage and costicartilage from DL3 and DL 4. **(D)** Nasopharynx and oropharynx from DL 4. **(E)** Fallopian tube from DL 2 and DL 7. **(F)** Pancreas, pancreas head, pancreas tail from DL 4. **(G)** Mammary gland from DL 2 and DL 4. Scale bars are indicated in the bottom right corner of each image.

Pan-tissue and tissue-specific remodeling during development and tumorigenesis

To characterize the dynamic landscape of proteomic remodeling across developmental and pathological states, we performed trajectory inference on fetal (F), tumor (T), paired non-tumor (NT), and normal (N) tissues using uniform manifold approximation and projection (UMAP) and pseudotime modeling. Analysis of 2847 human samples exhibited a continuous, ordered distribution of pseudotime—spanning F, N, NT, to T states—mirroring development and tumorigenesis (**Extended Data Fig. 3a**). This global trajectory was accompanied by distinct tissue-specific topologies: brain samples clustered closely, indicative of minimal proteomic divergence, whereas other tissues formed discrete subclusters (**Extended Data Fig. 3a**). Notably, brain tissues exhibited exceptional proteomic stability during malignant transformation and stayed at a low level of pseudotime, with low variance across F-N–NT–T axes (**Extended Data Fig. 3a**). These results highlight the unique resilience of the brain proteome and delineate tissue-specific trajectories of proteomic reorganization in tumorigenesis.

Unsupervised clustering analysis by clusterGIVs[17] exhibited both conserved and organ-specific molecular changes across the F–T–NT–N transition. Across all tissues, this transition consistently involved the progressive downregulation of RNA splicing and ribosome biogenesis, along with increased immune activation and metabolic reprogramming. However, substantial regulatory differences were also observed. In the liver, Ras signaling pathways peaked in clusters corresponding to T states. The brain, on the other hand, showed enrichment of adaptive immune-related pathways in similar clusters. Both organs exhibited increasing enrichment in metabolism as the trajectory progressed (F–T–NT–N). Notably, the brain exhibited a marked upregulation of synaptic transmission-related pathways along the trajectory (**Supplementary Fig. 8)**, reflecting ongoing neuronal maturation and circuit refinement in postnatal development[18,19], processes typically suppressed in tumorigenesis[20]. This sustained activation suggests a tissue-intrinsic differentiation program that remains partially preserved even in the transformed state.

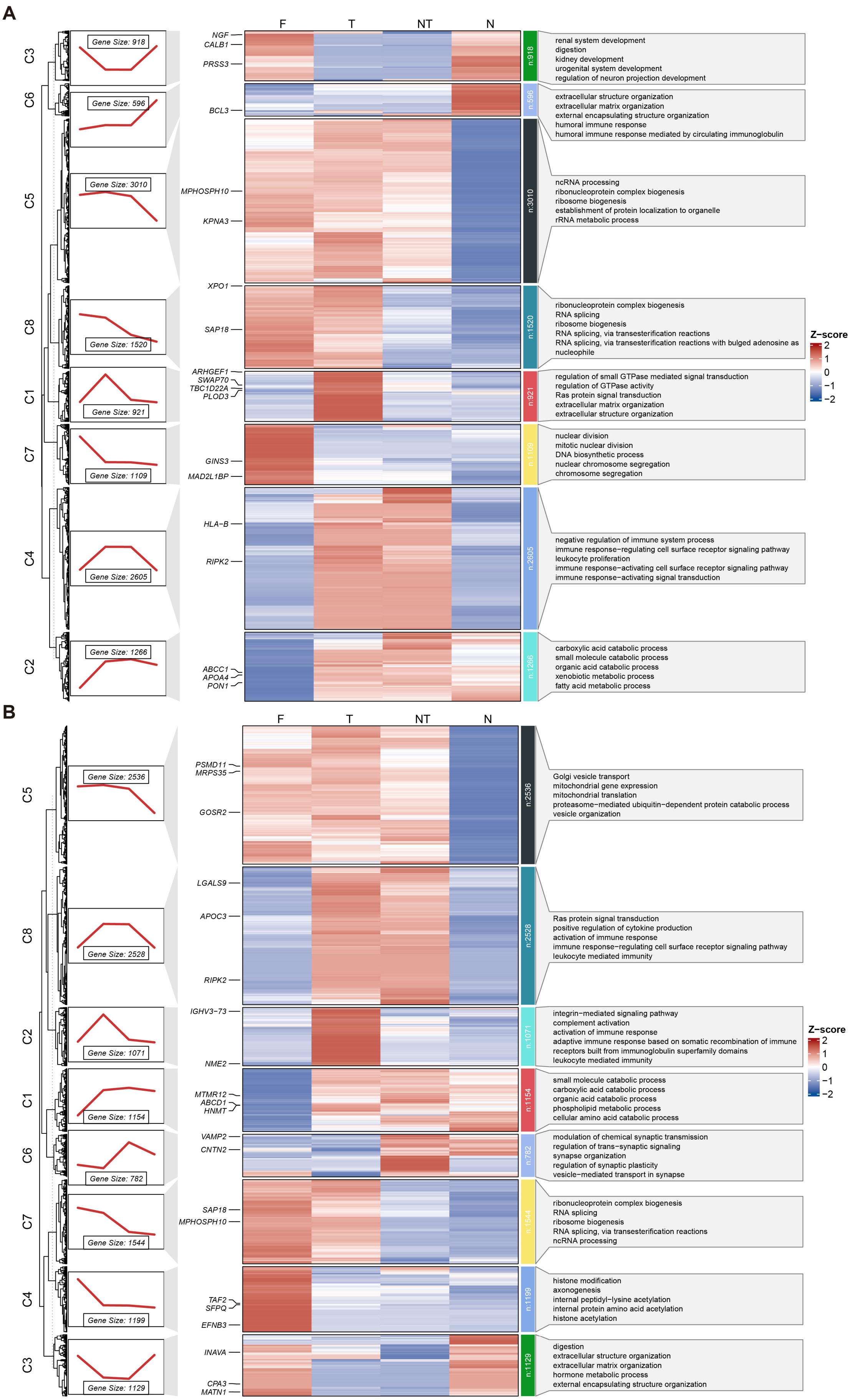
A
F
T
NT
N
C3
Gene Size: 918
NGF
CALB1
PRSS3
n:918
renal system development
digestion
kidney development
urogenital system development
regulation of neuron projection development
C6
Gene Size: 596
BCL3
n:596
extracellular structure organization
extracellular matrix organization
external encapsulating structure organization
humoral immune response
humoral immune response mediated by circulating immunoglobulin
C5
Gene Size: 3010
MPHOSPH10
KPNA3
n:3010
ncRNA processing
ribonucleoprotein complex biogenesis
ribosome biogenesis
establishment of protein localization to organelle
rRNA metabolic process
C8
Gene Size: 1520
XPO1
SAP18
n:1520
ribonucleoprotein complex biogenesis
RNA splicing
ribosome biogenesis
RNA splicing, via transesterification reactions
RNA splicing, via transesterification reactions with bulged adenosine as nucleophile
C1
Gene Size: 921
ARHGEF1
SWAP70
TBC1D22A
PLOD3
n:921
regulation of small GTPase mediated signal transduction
regulation of GTPase activity
Ras protein signal transduction
extracellular matrix organization
extracellular structure organization
C7
Gene Size: 1109
GINS3
MAD2L1BP
n:1109
nuclear division
mitotic nuclear division
DNA biosynthetic process
nuclear chromosome segregation
chromosome segregation
C4
Gene Size: 2605
HLA−B
RIPK2
n:2605
negative regulation of immune system process
immune response−regulating cell surface receptor signaling pathway
leukocyte proliferation
immune response−activating cell surface receptor signaling pathway
immune response−activating signal transduction
C2
Gene Size: 1266
ABCC1
APOA4
PON1
n:1266
carboxylic acid catabolic process
small molecule catabolic process
organic acid catabolic process
xenobiotic metabolic process
fatty acid metabolic process
Z−score
2
1
0
−1
−2
B
F
T
NT
N
C5
Gene Size: 2536
PSMD11
MRPS35
GOSR2
n:2536
Golgi vesicle transport
mitochondrial gene expression
mitochondrial translation
proteasome−mediated ubiquitin−dependent protein catabolic process
vesicle organization
C8
Gene Size: 2528
LGALS9
APOC3
RIPK2
n:2528
Ras protein signal transduction
positive regulation of cytokine production
activation of immune response
immune response−regulating cell surface receptor signaling pathway
leukocyte mediated immunity
C2
Gene Size: 1071
IGHV3−73
NME2
n:1071
integrin−mediated signaling pathway
complement activation
activation of immune response
adaptive immune response based on somatic recombination of immune receptors built from immunoglobulin superfamily domains
leukocyte mediated immunity
C1
Gene Size: 1154
MTMR12
ABCD1
HNMT
n:1154
small molecule catabolic process
carboxylic acid catabolic process
organic acid catabolic process
phospholipid metabolic process
cellular amino acid catabolic process
C6
Gene Size: 782
VAMP2
CNTN2
n:782
modulation of chemical synaptic transmission
regulation of trans−synaptic signaling
synapse organization
regulation of synaptic plasticity
vesicle−mediated transport in synapse
C7
Gene Size: 1544
SAP18
MPHOSPH10
n:1544
ribonucleoprotein complex biogenesis
RNA splicing
ribosome biogenesis
RNA splicing, via transesterification reactions
ncRNA processing
C4
Gene Size: 1199
TAF2
SFPQ
EFNB3
n:1199
histone modification
axonogenesis
internal peptidyl−lysine acetylation
internal protein amino acid acetylation
histone acetylation
C3
Gene Size: 1129
INAVA
CPA3
MATN1
n:1129
digestion
extracellular structure organization
extracellular matrix organization
hormone metabolic process
external encapsulating structure organization
Z−score
2
1
0
−1
−2

Supplementary Figure 8. Heatmap highlighting trajectory-associated proteins across biological states in liver (**A**) and brain (**B**) samples. The protein matrix was pre-filtered to exclude contaminant proteins followed by differential expression analysis using one-way ANOVA across sample types. Proteins with significance (B-H adjusted $P < 0.05$) were clustered into eight co-expression modules (M1–M8) based on expression in F, T, NT, N, with gene size indicating the number of proteins within each module. Z-scores reflect relative protein abundance across the columns (F, T, NT, and N). On the right, each module is annotated with its top five enriched Gene Ontology Biological Process (GOBP) terms, ranked by $-\log_{10}$(B-H adjusted P) for pathway enrichment. Representative proteins belonging to the top-ranked GOBP term of each module are labeled on the left.

Comparison with the previous human transcriptome and proteome draft

Multiple cross-tissue studies have shown that RNA–protein correlations across tissues are only moderate[21], and our data align with this trend, showing median Pearson correlations of 0.25 (HPA[22]) and 0.24 (GTEx[23]) (**Table S4**). Poorly RNA-correlated proteins are mainly enriched in post-transcriptional regulation, including translational efficiency, protein complex, and RNA splicing (**Table S4**), which is consistent with a previous study[24].

Besides the differences in tissue-enriched proteins between our and previous studies mentioned in the main text, group-enriched proteins also vary due to the tissues only in our dataset. For example, we found a protein grouped enriched in bone, bone marrow, and auditory ossicles named osteocalcin (BGLAP), which was classified into tissue-enhanced proteins in choroid plexus and intestine in HPA (**Table S5**). BGALP, a 10.96 kDa protein of 100 amino acids, is mainly produced by osteoblasts[25]. The misclassification by HPA likely stems from the lack of bone-derived samples in their dataset, where BGALP shows low transcription across tissues (<10 transcripts per million), with relatively higher levels in the choroid plexus and intestine. Our database offers a resource for further exploration of bone's multifaceted biological roles.

Dysregulated proteins as the drug targets in clinical trials

To further explore the potential therapeutic use of the DEPs, we mapped the 7427 DEPs found in all cancer types to the protein targets of biotechnical and large molecule drugs in DrugBank[26,27] and ongoing clinical trials. A total of 163 DEPs were identified as drug targets corresponding to 237 drug candidates (**Table S7**). These findings underscore the vast potential of this dataset for the discovery of novel drug targets.

The dysregulations of certain protein targets suggested viable and approved therapy strategies for cancers. Usually, there are some well-studied or approved drugs for certain protein targets in cancer treatment, but their efficacy in other cancer types remains understudied. Actually, it is rational that a verified upregulated protein across different cancer types might also be a potential drug target for the approved drug, even if in other types of cancers.

To discover potential cancer therapies under investigation, we searched 1266 pairs of cancer indications-drug entries constituted by the 237 drug candidates and their targeted cancers on the

ClinicalTrials.gov database (version as of June 2, 2023). We obtained 2084 clinical trials related to 36 drugs and 77 up-regulated proteins in 18 cancers with the last update posted from 2005 to 2023 (**Extended Data Fig. 8a**). The drugs were classified into the following kinds according to their mechanism: immunotherapy (41.67%), receptor tyrosine kinase (RTK) inhibitor (27.78%), antibody (11.11%), antibody-drug conjugate (ADC) (8.33%), hormonal agent (8.33%), and angiogenesis inhibitor (2.78%). Excluding 203 trials with unavailable phases, 16.37% (308) are in phase 1 or early phase 1, 63.85% (1201) are in phase 2, and 18.08% (340) are in phase 3. Only 1.70% (32) of the trials were in phase 4 and matched to 8 drugs, twelve of which were related to the use of Bevacizumab as a vascular endothelial growth factor A (VEGFA) inhibitor for the treatment of ovarian carcinoma (OC) and endometrial carcinoma (ENCA)[28]. Bevacizumab was also used for non-small cell lung cancer, colorectal cancer, renal cell carcinoma, and cervical cancer, supported by phase 3 clinical trials, but these indications were not mapped because VEGFA was not identified as being dysregulated in these types. Besides, few ADC drugs were found. Enfortumab vedotin, an ADC drug targeting Nectin-4, is used in combination with pembrolizumab to treat advanced urothelial cancer in a phase 3 clinical trial[29]. Although not yet approved by the Food and Drug Administration, Enfortumab vedotin is expected to be approved in subsequent trials. More annotations of clinical trials for DEPs could be found in **Table S7**. Beyond these protein targets mapped with DrugBank and clinical trials, we believe that this database would further facilitate the development of new therapies or biomarkers, as targeting dysregulated proteins or related pathways has been frequently applied in biomedical research.

References

1 Jiang, L. *et al.* A Quantitative Proteome Map of the Human Body. *Cell* **183**, 269-283.e219 (2020). https://doi.org:10.1016/j.cell.2020.08.036

2 Ding, Y. *et al.* Comprehensive human proteome profiles across a 50-year lifespan reveal aging trajectories and signatures. *Cell* **188**, 5763-5784.e5726 (2025). https://doi.org:10.1016/j.cell.2025.06.047

3 Uhlen, M. *et al.* A pathology atlas of the human cancer transcriptome. *Science* **357** (2017). https://doi.org:10.1126/science.aan2507

4 Gao, H. *et al.* Accelerated Lysis and Proteolytic Digestion of Biopsy-Level Fresh-Frozen and FFPE Tissue Samples Using Pressure Cycling Technology. *J Proteome Res* **19**, 1982-1990 (2020). https://doi.org:10.1021/acs.jproteome.9b00790

5 Guo, T. *et al.* Rapid mass spectrometric conversion of tissue biopsy samples into permanent quantitative digital proteome maps. *Nat Med* **21**, 407-413 (2015). https://doi.org:10.1038/nm.3807

6 Yu, F. *et al.* Analysis of DIA proteomics data using MSFragger-DIA and FragPipe computational platform. *Nature Communications* **14**, 4154 (2023). https://doi.org:10.1038/s41467-023-39869-5

7 da Veiga Leprevost, F. *et al.* Philosopher: a versatile toolkit for shotgun proteomics data analysis. *Nature Methods* **17**, 869-870 (2020). https://doi.org:10.1038/s41592-020-0912-y

8 Yu, F. *et al.* Fast Quantitative Analysis of timsTOF PASEF Data with MSFragger and IonQuant. *Molecular & cellular proteomics : MCP* **19**, 1575-1585 (2020).

https://doi.org:10.1074/mcp.TIR120.002048

9 Kong, A. T., Leprevost, F. V., Avtonomov, D. M., Mellacheruvu, D. & Nesvizhskii, A. I. MSFragger: ultrafast and comprehensive peptide identification in mass spectrometry-based proteomics. *Nat Methods* **14**, 513-520 (2017). https://doi.org:10.1038/nmeth.4256

10 Kim, M. S. *et al.* A draft map of the human proteome. *Nature* **509**, 575-581 (2014). https://doi.org:10.1038/nature13302

11 Desiere, F. *et al.* The PeptideAtlas project. *Nucleic Acids Res* **34**, D655-658 (2006). https://doi.org:10.1093/nar/gkj040

12 Wilhelm, M. *et al.* Mass-spectrometry-based draft of the human proteome. *Nature* **509**, 582-587 (2014). https://doi.org:10.1038/nature13319

13 Liang, S. *et al.* Large-scale metaproteomics of human gut microbiota reveals microbial functions in metabolic diseases and aging. *Cell Metabolism* **38**, 995-1011.e1016 (2026). https://doi.org:10.1016/j.cmet.2026.02.012

14 Laatsch, C. N. *et al.* Human hair shaft proteomic profiling: individual differences, site specificity and cuticle analysis. *PeerJ* **2**, e506 (2014). https://doi.org:10.7717/peerj.506

15 Parker, G. J. *et al.* Demonstration of Protein-Based Human Identification Using the Hair Shaft Proteome. *PLoS One* **11**, e0160653 (2016). https://doi.org:10.1371/journal.pone.0160653

16 Goecker, Z. C. *et al.* Optimal processing for proteomic genotyping of single human hairs. *Forensic science international. Genetics* **47**, 102314 (2020). https://doi.org:10.1016/j.fsigen.2020.102314

17 Zhang, J. ClusterGVis: one-step to cluster and visualize gene expression matrix. *GitHub. https://github. com/junjunlab/ClusterGVis* (2022).

18 Velmeshev, D. *et al.* Single-cell analysis of prenatal and postnatal human cortical development. *Science* **382**, eadf0834 (2023). https://doi.org:10.1126/science.adf0834

19 Stiles, J. & Jernigan, T. L. The basics of brain development. *Neuropsychology review* **20**, 327-348 (2010). https://doi.org:10.1007/s11065-010-9148-4

20 Shen, L. *et al.* Mechanistic insight into glioma through spatially multidimensional proteomics. *Sci Adv* **10**, eadk1721 (2024). https://doi.org:10.1126/sciadv.adk1721

21 Liu, Y., Beyer, A. & Aebersold, R. On the Dependency of Cellular Protein Levels on mRNA Abundance. *Cell* **165**, 535-550 (2016). https://doi.org:10.1016/j.cell.2016.03.014

22 Uhlén, M. *et al.* Proteomics. Tissue-based map of the human proteome. *Science* **347**, 1260419 (2015). https://doi.org:10.1126/science.1260419

23 The, G. C. *et al.* The GTEx Consortium atlas of genetic regulatory effects across human tissues. *Science* **369**, 1318-1330 (2020). https://doi.org:10.1126/science.aaz1776

24 Nusinow, D. P. *et al.* Quantitative Proteomics of the Cancer Cell Line Encyclopedia. *Cell* **180**, 387-402.e316 (2020). https://doi.org:10.1016/j.cell.2019.12.023

25 Zoch, M. L., Clemens, T. L. & Riddle, R. C. New insights into the biology of osteocalcin. *Bone* **82**, 42-49 (2016). https://doi.org:10.1016/j.bone.2015.05.046

26 Wishart, D. S. *et al.* DrugBank 5.0: a major update to the DrugBank database for 2018. *Nucleic Acids Res* **46**, D1074-D1082 (2018). https://doi.org:10.1093/nar/gkx1037

27 Wishart, D. S. *et al.* DrugBank: a comprehensive resource for in silico drug discovery and exploration. *Nucleic Acids Res* **34**, D668-672 (2006). https://doi.org:10.1093/nar/gkj067

28 Stewart, J. *Avastin FDA Approval History*, <https://www.drugs.com/history/avastin.html> (2021).

29 Hoimes, C. J. *et al.* Enfortumab Vedotin Plus Pembrolizumab in Previously Untreated Advanced Urothelial Cancer. *J Clin Oncol* **41**, 22-31 (2023). https://doi.org:10.1200/JCO.22.01643
