# Supplementary material for: Spatial distribution of the proteome in the human body and in cancers
Source: Nature. 2026 Jun 17;656(8126):227–36. doi: 10.1038/s41586-026-10660-y (PMC13442039; doi:10.1038/s41586-026-10660-y)
Supplement: Supplementary file 3 [file 41586_2026_10660_MOESM2_ESM.pdf]

nature portfolio

Corresponding author(s): Tiannan Guo, Yi Zhu, Yan Li, Yong Ji

Last updated by author(s): Apr 11, 2026

Reporting Summary

Nature Portfolio wishes to improve the reproducibility of the work that we publish. This form provides structure for consistency and transparency in reporting. For further information on Nature Portfolio policies, see our Editorial Policies and the Editorial Policy Checklist.

Statistics

For all statistical analyses, confirm that the following items are present in the figure legend, table legend, main text, or Methods section.

n/a	Confirmed	
☐	☒	The exact sample size (*n*) for each experimental group/condition, given as a discrete number and unit of measurement
☐	☒	A statement on whether measurements were taken from distinct samples or whether the same sample was measured repeatedly
☐	☒	The statistical test(s) used AND whether they are one- or two-sided<br>*Only common tests should be described solely by name; describe more complex techniques in the Methods section.*
☐	☒	A description of all covariates tested
☐	☒	A description of any assumptions or corrections, such as tests of normality and adjustment for multiple comparisons
☐	☒	A full description of the statistical parameters including central tendency (e.g. means) or other basic estimates (e.g. regression coefficient) AND variation (e.g. standard deviation) or associated estimates of uncertainty (e.g. confidence intervals)
☐	☒	For null hypothesis testing, the test statistic (e.g. *F*, *t*, *r*) with confidence intervals, effect sizes, degrees of freedom and *P* value noted<br>*Give P values as exact values whenever suitable.*
☐	☒	For Bayesian analysis, information on the choice of priors and Markov chain Monte Carlo settings
☐	☒	For hierarchical and complex designs, identification of the appropriate level for tests and full reporting of outcomes
☐	☒	Estimates of effect sizes (e.g. Cohen's *d*, Pearson's *r*), indicating how they were calculated

Our web collection on statistics for biologists contains articles on many of the points above.

Software and code

Policy information about availability of computer code

Data collection	For generating consensus PASEF spectral libraries, we used FragPipe computational platform (version 18) with MSFragger (version 3.5), Philosopher (version 4.2.2) components, and EasyPQP (https://github.com/grosenberger/easypqp/) (version 0.1.29) Python package. And our workflow was based on the FragPipe DIA_SpecLib_Quant workflow. The DIA quantitative matrix were generated by DIA-NN (version 2.2.0 Academia). The PRM quantitative matrix were generated by Skyline (version 21.1). HE staining images were captured by Zeiss Axio Observer 3 and processed by ZEN blue (version 3.4).
Data analysis	Analyses were performed in R (v4.4.0) using arrow (v23.0.1.2), data.table (v1.14.10), dplyr (v1.1.4), magrittr (v2.0.3), plyr (v1.8.9), readr (v2.1.4), readxl (v1.4.3), reshape2 (v1.4.4), rio (v1.3.0), stringr (v1.6.0), tidyr (v1.3.0), tidyverse (v2.0.0), writexl (v1.5.4), openxlsx (v4.2.5.2) and rstudioapi (v0.18.0) for data handling; preprocessCore (v1.65.0), sva (v3.51.0) and imputeLCMD (v2.1) for preprocessing and normalization; biocParallel (v1.38.0), doParallel (v1.0.17), foreach (v1.5.2), parallel (v4.4.1), stats (v4.4.1), limma (v3.59.1), lme4 (v1.1.35.1), lmerTest (v3.1-3) and broom (v1.0.12) for statistical analysis; cluster (v2.1.4), ConsensusClusterPlus (v1.67.0), Rtsne (v0.16), umap (v0.2.10.0) and RSpectra (v0.16.2) for clustering and dimensionality reduction; randomForest (v4.7.1.2) for machine learning; AnnotationDbi (v1.65.2), org.Hs.eg.db (v3.18.0), clusterProfiler (v4.12.6), GSEABase (v1.65.1), GSVA (v1.51.1), msigdbr (v7.5.1), ReactomePA (v1.47.0) and seqinr (v4.2.36) for annotation and enrichment analyses; and ggplot2 (v4.0.1), ggpubr (v0.6.0), pheatmap (v1.0.12), circlize (v0.4.15), corrplot (v0.92), ggalluvial (v0.12.5), ggraph (v2.1.0), igraph (v1.6.0), VennDiagram (v1.7.3), cowplot (v1.1.1), patchwork (v1.1.2), ggrepel (v0.9.4), viridis (v0.6.4), RColorBrewer (v1.1-3), scales (v1.4.0), grid (v4.4.1), ggsci (v4.2.0), ggstatsplot (v0.13.6), ggridges (v0.5.5) and ClusterGVis (v0.1.4) for visualization; palmerpenguins (v0.1.1) was used for dataset demonstration.<br>Custom code developed for statistical analysis is publicly available at GitHub: https://github.com/guomics-lab/TPHP.

For manuscripts utilizing custom algorithms or software that are central to the research but not yet described in published literature, software must be made available to editors and reviewers. We strongly encourage code deposition in a community repository (e.g. GitHub). See the Nature Portfolio guidelines for submitting code & software for further information.

Data

Policy information about availability of data

All manuscripts must include a data availability statement. This statement should provide the following information, where applicable:

- Accession codes, unique identifiers, or web links for publicly available datasets
- A description of any restrictions on data availability
- For clinical datasets or third party data, please ensure that the statement adheres to our policy

All the qualitative and quantitative data utilized for analysis in this paper are openly accessible and fully available. Due to the limited publication length, the proteome data of some specific tissues or carcinomas were shown and discussed. To better visualize and present the entire database, we built a website (https://db.prottalks.com/), supporting both protein-centric and tissue-centric queries. Specifically, pairs of interested tissues can undergo differential expression analysis online, and the DEPs are shown in an instant heatmap. Raw data as well as corresponding metadata annotations following the Sample and Data Relationship Format (SDRF-Proteomics) standard are available for download at https://www.iprox.org/ (IPX0003578000) and https://www.proteomexchange.org/ (PXD063370, Token: 6ShvG10D5X4i).

Research involving human participants, their data, or biological material

Policy information about studies with human participants or human data. See also policy information about sex, gender (identity/presentation), and sexual orientation and race, ethnicity and racism.

Reporting on sex and gender	Participant demographics (sex and age) and diagnosis are reported in Table S1.
Reporting on race, ethnicity, or other socially relevant groupings	Data on race, ethnicity, or other socially relevant characteristics were not collected in this study
Population characteristics	Participant demographics (sex and age) and diagnosis are reported in Table S1.
Recruitment	We confirm that no self-selection bias was introduced during the recruitment process. Autopsy donors were enrolled using a non-selective recruitment strategy, while ensure the gender balance.<br>Pancancer patient recruitment was restricted by the collection period and adhered to strict clinical criteria to minimize confounding variables:<br>Treatment-naïve status: Only patients who had not received prior chemotherapy, radiotherapy, or targeted therapies were included to ensure the baseline molecular profile of the primary tumor.<br>Comorbidity control: Priority was given to patients without complex systemic comorbidities, such as hypertension or diabetes mellitus, to reduce metabolic or systemic noise in the data.
Ethics oversight	The autopsy samples involved in this study were collected from the body donation center of Dalian Medical University and Shanghai Jiao Tong University. The project has been approved by the Ethics Committee of Dalian Medical University (IRB: Dalian Medical 2019-05) and Shanghai Jiao Tong University (IRB: 2022-A-01, 2021-03). Written informed consent was obtained from the Legally Authorized Representatives for all donors.<br>Paired tumors and non-tumor samples from surgery were collected from Harbin Medical University Cancer Hospital and Huashan Hospital. The project has been approved by the Ethics Committee of Harbin Medical University Cancer Hospital (IRB: KY2019-08, KY2023-03, KY2019-16) and Huashan Hospital (KY2021-064). Written informed consent for each patient was collected.<br>Autopsy specimens of fetuses were collected from Shenzhen Baoan District Maternal and Child Health Hospital (during 20200616 to 20200916), who died of abortion. The project has been approved by Ethics Committee of Shenzhen Baoan District Maternal and Child Health Hospital (IRB: LLSCHY 2019-10-36). Written informed consent was obtained from all donors of tissues.

Note that full information on the approval of the study protocol must also be provided in the manuscript.

Field-specific reporting

Please select the one below that is the best fit for your research. If you are not sure, read the appropriate sections before making your selection.

☒ Life sciences ☐ Behavioural & social sciences ☐ Ecological, evolutionary & environmental sciences

For a reference copy of the document with all sections, see nature.com/documents/nr-reporting-summary-flat.pdf

Life sciences study design

All studies must disclose on these points even when the disclosure is negative.

Sample size	No statistical methods were used to predetermine sample sizes. The sample size for post-mortem (autopsy) tissues was set at a minimum of two donors (including one males and one female), with at least two biological replicates per tissue type to capture fundamental inter-individual biological variation. After multiple rounds of data refinement and sample supplementation, we have 9 donors of autopsy samples (5

females and 4 males). For the pan-cancer cohort, the sample size was primarily determined by the availability of clinical specimens within the project's predefined timeframe and the constraints of sequencing throughput. Based on previously published proteomic studies in cancer cohort [Coscia et al., Cell, 2018; Hengeveld et al., HemaSphere, 2023], a cohort size of approximately 40 patients per cancer type was deemed sufficient to identify pathological features and achieve representative biological insights. Detailed cohort size information for each cancer type is available in Table S1.

Data exclusions

A total of 3005 DIA files (2856 sample runs including replicates, and pooled sample runs) were acquired and searched to generate protein matrix. Next, we performed quality control (QC) analysis on the quantification results of the obtained protein matrix. First, files with low protein identifications were removed. Specifically, a file was excluded if its protein ID count was below the lower outlier (1.5 times the interquartile range below the first quartile) for its specific combination of major and detailed tissue type, and sample type. A file was filtered out only if it met this lower outlier criterion for both its major and detailed tissue type thresholds. This strict filtering excluded 48 files. After QC, we removed 149 pool samples before the downstream data analysis. Before calculating the coefficient of variations (CVs) and the correlation of the pooled sample files and replicates, we removed low identification pools and replicates first. One pool and 15 technical replicates were removed. This removed pool sample had a supplementary injection in the same batch. Before tissue specificity analysis, we identified and removed 35 files of abnormal intra- or inter-tissue similarity and HE staining, which might be due to autolytic necrosis (pancreas, tissue contamination (vagina), or unproper punching area (nerve,cartilage) as inferred by HE staining.

Replication

We employed both biological replicates (independent processing of aliquots from the same tissue sample) and technical replicates (repeated injections of identical peptide samples), with random assignment to minimize bias. Quality control utilized a dual approach: a project-specific pool prepared by mixing equal amounts of peptides from early-collected samples, and a universal standard (mouse liver digest) monitored by specialized MS administrators. Both quality control samples were injected before each batch to monitor instrument stability. All samples, including project-specific pool samples, were analyzed by DIA-NN and pre-processed together (3005 raw files in total).We found that most CVs of the protein abundances in the pooled peptide samples and replicates were low, with median values of 0.17 in pools (n = 148), 0.05 in technical replicates (n = 218), and 0.06 in biological replicates (n = 33), respectively (Figure S2C). Most of the correlation coefficients of protein abundances among the pooled samples and the replicates were high, with a median Pearson's correlation coefficient of 0.90 in pools (n = 148), 0.92 in technical replicates (n = 218), and 0.90 in biological replicates (n = 33), respectively (Figure S2D). Besides, we filtered a total of 2239 tumor and non-tumor clinical samples, as well as companion pooled peptide samples (Figure S3A). We evaluated the reproducibility and batch effect in the pan-cancer proteomic matrix. We found that most CVs of the protein abundances in the 92 pooled peptide samples, 163 technical replicates, and 13 biological replicates were low, with median values of 0.18, 0.06, and 0.07, respectively (Figure S3B). Most of the correlation coefficients of protein abundances among pooled peptide samples and replicates were high, with a median Pearson's correlation coefficient of 0.86 for pools, 0.92 for technical replicates, and 0.91 for biological replicates (Figure S3C).

Randomization

Samples were randomized by reshuffling each collection batch, and clinical metadata were blinded during processing.

Blinding

Clinical samples were re-coded with anonymous identifiers during processing, and laboratory personnel were blinded to all clinical information.

Reporting for specific materials, systems and methods

We require information from authors about some types of materials, experimental systems and methods used in many studies. Here, indicate whether each material, system or method listed is relevant to your study. If you are not sure if a list item applies to your research, read the appropriate section before selecting a response.

Materials & experimental systems

n/a	Involved in the study
☒	☐ Antibodies
☒	☐ Eukaryotic cell lines
☒	☐ Palaeontology and archaeology
☒	☐ Animals and other organisms
☒	☐ Clinical data
☒	☐ Dual use research of concern
☒	☐ Plants

Methods

n/a	Involved in the study
☒	☐ ChIP-seq
☒	☐ Flow cytometry
☒	☐ MRI-based neuroimaging

Plants

Seed stocks

NA

Novel plant genotypes

NA

Authentication

NA
